# Epirubicin and gait apraxia: a real-world data analysis of the FDA Adverse Event Reporting System database

**DOI:** 10.3389/fphar.2023.1249845

**Published:** 2023-09-14

**Authors:** Wei Wang, Xin Guan, Shuang Wang, Lipeng Shi, Yanfei Zhu, Pengbo Hua, Qiang Guo, Jingqi Wang

**Affiliations:** ^1^ Department of Urology, Second Hospital of Shanxi Medical University, Taiyuan, China; ^2^ Department of Cardiology, Shanxi Bethune Hospital, Shanxi Academy of Medical Sciences, Tongji Shanxi Hospital, Third Hospital of Shanxi Medical University, Taiyuan, China; ^3^ Department of Gastroenterology, Shanxi Bethune Hospital, Shanxi Academy of Medical Sciences, Tongji Shanxi Hospital, Third Hospital of Shanxi Medical University, Taiyuan, China; ^4^ Department of General Surgery, Second Hospital of Shanxi Medical University, Taiyuan, China

**Keywords:** epirubicin, FDA Adverse Event Reporting System database, safety, reporting odds ratio (ROR), proportional reporting ratio (PRR), bayesian confidence propagation neural network (BCPNN), empirical Bayes geometric mean (EBGM)

## Abstract

**Introduction:** Epirubicin is widely used in many malignancies with good efficacy and tolerability. However, investigations about adverse events (AEs) using real-world information are still insufficient.

**Methods:** We extracted Epirubicin-related reports submitted between the first quarter of 2014 and first quarter of 2023 from FAERS database. Four algorithms were utilized to evaluate whether there was a significant correlation between Epirubicin and AEs.

**Results:** After de-duplicating, a total of 3919 cases were extracted. Among the 3919 cases, we identified 1472 AEs, 253 of which were found to be adverse drug reactions (ADRs) associated with Epirubicin. We analysed the occurrence of Epirubicin-induced ADRs and found several unexpected significant ADRs, such as hepatic artery stenosis, hepatic artery occlusion, intestinal atresia and so on. Interestingly, we found gait apraxia, a neurological condition, was also significantly associated with Epirubicin. To our knowledge, there haven't studies that have reported an association between gait disorders and the usage of epirubicin.

**Discussion:** Our study identified new unexpected significant ADRs related to Epirubicin, providing new perspectives to the clinical use of Epirubicin.

## Introduction

Epirubicin, the 4′-epimer of doxorubicin, was introduced into clinical use more than 40 years ago and soon established a vital place in cancer chemotherapy (Mouridsen et al., 1990; Pavone-MacalusoTripi et al., 1993). Epirubicin and doxorubicin have different properties due to the different spatial orientation of the hydroxyl group at 4'. Compared to doxorubicin, epirubicin has been shown to cause less myelosuppression, fewer cardiac toxicities, fewer non-hematologic toxicities, and less nausea and vomiting (Henderson et al., 2003; Burnell et al., 2010). The anti-tumor mechanisms of epirubicin appear to intercalate with DNA, inhibit the activity of topoisomerase II, generate free radicals and oxygen, and thus disturb the synthesis of DNA, RNA, and proteins, resulting in its anti-tumor function (Khasraw et al., 2012).

Since its approval by the U.S. Food and Drug Administration (FDA), epirubicin has been widely used to treat many malignancies, including bladder cancer, breast cancer, liver cancer, and non-small cell lung cancer (NSCLC) (Plosker and Faulds, 1993; de Azambuja et al., 2009). Bladder cancer accounts for approximately 90%–95% of urothelial cancers and 4% of malignant cancers in adults (Richters et al., 2020). Since 1985, many studies have shown that epirubicin has good therapeutic effects and high efficacy in preventing bladder cancer recurrence (Tripi et al., 1986; Oosterlinck et al., 1993). Previous clinical trials have shown that, in patients with bladder cancer, the main adverse events of epirubicin are hematuria, cystitis, urinary tract infection, and systemic side effects, which include fever, tiredness, nausea, abdominal pain, and headache—listed on the instruction label (Onrust et al., 1999; Hendricksen et al., 2008). Hepatocellular carcinoma (HCC) accounts for approximately 90% of liver cancer (Forner et al., 2018). Transarterial chemoembolization using doxorubicin (TACE-DOX) is recommended for the therapy of hepatocellular carcinoma (Laface et al., 2021). According to Ellis et al. (1995), epirubicin is well tolerated by patients with hepatocellular carcinoma and has a good curative effect. A study published in 2015 suggested that, compared to patients with hepatocellular carcinoma treated with miriplatin, patients treated with miriplatin-plus-epirubicin were more likely to develop adverse events such as neutropenia and anorexia (Tawada et al., 2015). These adverse events (AEs) have been marked on the instruction label; however, it is worth noting that the majority of safety-related information comes from premarket randomized controlled trials (RCTs) and a few case reports on AEs; these lack extensive real-world data.

A thorough understanding of their possible side effects is essential for the safe use of drugs. Given the limitations of clinical trials and case reports, public databases containing extensive information on drug use, such as the FDA Adverse Event Reporting System (FAERS), have emerged. FAERS contains large-scale information submitted by healthcare professionals, consumers, and manufacturers; therefore, it can help identify AEs that have not been marked on the instruction label (Sakaeda et al., 2013). In this study, the AEs of epirubicin were identified and analyzed using four rigorous algorithms based on the FAERS database. AEs not specified on the medicine labels were observed when the safety of epirubicin was reviewed and analyzed. The findings of this study offer suggestions for the therapeutic application of epirubicin.

## Methods

### Data source and preprocessing

The FAERS database, containing various reports of spontaneous adverse events and side effects submitted by consumers, healthcare professionals, and manufacturers, has worldwide coverage and large-scale information, making it suitable for the early detection of safety signals and timely characterization of the safety profile (Ji et al., 2019).

This study aimed to evaluate the safety of epirubicin. AE data related to epirubicin submitted between the first quarter of 2014 and the first quarter of 2023 from the FAERS database were extracted and subsequently preprocessed using SAS and Navicat for MySQL software. After cleaning and standardization, it was mapped to MedDRA to identify adverse drug reactions (ADRs) (Ma et al., 2005). Significant ADRs were calculated and mapped to preferred terms (PTs) and system organ class (SOC), which represent the different levels of MedDRA (Brown, 2004). The specific information extracted for this study included age, sex, and country. Serious clinical outcomes were classified as disability, death, hospitalization, and life-threatening events.

### Statistical analysis

Multiple algorithms, including ROR, PRR, MHRA, and BCPNN, were used to assess whether epirubicin was significantly associated with AEs. In the formula, the value a represents the total number of AEs for epirubicin, b represents the total number of cases for this specific AE for all other drugs, c represents the total number of other AEs for epirubicin, and d represents the total number of other AEs for all other drugs (Almenoff et al., 2006). The specific formulae are as follows.

#### ROR

$$\begin{array}{c} \text{ROR}=\frac{\text{ad}}{\text{bc}},\\ {95\%\;\text{CI}=e}^{\ln\left(ROR\right)\pm 1.96\sqrt{\left(\frac{1}{a}+\frac{1}{b}+\frac{1}{c}+\frac{1}{d}\right)}}. \end{array}$$

If the lower limit of 95% CI > 1 and a≥3, the ROR should be considered a signal.

#### PRR

$$\begin{array}{c} PRR=\frac{a/\left(a+b\right)}{c/\left(c+d\right)},\\ {95\%\;\text{CI}=e}^{\ln\left(PRR\right)\pm 1.96\sqrt{\left(\frac{1}{a}-\frac{1}{a+b}+\frac{1}{c}-\frac{1}{c+d}\right)}}. \end{array}$$

If a≥3 and the lower limit of 95% CI > 1, the PRR should be considered a signal.

#### BCPNN

$$\begin{array}{c} \text{IC}=\log_{2}\frac{a\left(a+b+c+d\right)}{\left(a+b\right)\left(a+c\right)},\\ \text{IC}-2\text{SD}=E\left(\text{IC}\right)-2\sqrt{V\left(\text{IC}\right)}. \end{array}$$

If IC-2SD ≤ 0, it should be identified as no signal (−); if 0< IC-2SD ≤ 1.5, it should be identified as a weak signal (+); if 1.5< IC-2SD ≤ 3, it should be identified as a medium signal (++); if IC-2SD > 3, it should be identified as a strong signal (+++). IC-2SD > 0 is identified as the cutoff value for a positive signal.

#### EBGM

$$\begin{array}{c} \text{EBGM}=\frac{a\left(a+b+c+d\right)}{\left(a+c\right)\left(a+b\right)},\\ 95\%\text{ CI}=e^{\ln\left(\text{EBGM}\right)\pm 1.96\sqrt{\left(\frac{1}{a}+\frac{1}{b}+\frac{1}{c}+\frac{1}{d}\right)}}. \end{array}$$

If EBGM05 > 2, it should be considered a signal.

## Results

### Descriptive analysis

A total of 13,703,053 reports submitted between the first quarters of 2014 and 2023 were extracted from the FAERS database. After deduplicating, 3,919 cases were used for subsequent analyses. Among these, we identified 1,472 AEs, of which 253 were found to be ADRs associated with epirubicin. The clinical characteristics of the patients are summarized in Table 1.

**TABLE 1:** The characters of case reports associated with Epirubicin as primary suspected drug from 2014 Q1 to 2023 Q1.

	Epirubicin	
	Count	Percentage
Number of events	3919	
Gender		
Male	468	11.94%
Female	2560	65.32%
Unknown	891	22.74%
Age		
<20	87	2.22%
20-29	68	1.74%
30-39	250	6.38%
40-49	632	16.13%
50-59	773	19.72%
60-69	617	15.74%
70-79	298	7.60%
≥80	46-1	1.17%
Unknown	1148	29.29%
Reported Countries (the top ranked)		
CN (China)	1002	25.27%
IT (Italy)	620	15.82%
FR (France)	463	11.81%
GB (Great Britain)	459	11.71%
DE (Germany)	280	7.14%
Serious Outcomes		
Death	221	5.64%
Disability	111	2.83%
Hospitalization	1311	33.45%
Life-threatening	221	5.64%

Of the 3,919 cases, 2,560 were females (65.32%) and 468 were males (11.94%); 891 were unspecified. The analysis showed that individuals aged 50–59 were more likely to experience AEs, accounting for 773 (19.72%) cases. The five countries with the highest frequency of epirubicin use were China (1,002, 25.27%), Italy (620, 15.82%), France (463, 11.81%), Great Britain (459, 11.71%), and Germany (280, 7.14%). Our results showed that the top five severe outcomes were hospitalization (1,311, 33.45%), life-threatening events (221, 5.64%), death (221, 5.64%), and disability (111, 2.83%).

### Signal detections at the system organ class level

As shown in Table 2, the top three ADRs ranked by case numbers at the SOC level were blood and lymphatic system disorders [case numbers: 1011, ROR (95% CI) = 7.00 (6.56–7.47), PRR (95% CI) = 6.47 (6.10–6.87), IC (IC025) = 2.69 (2.59), and EBGM (EBGM05) = 6.46 (6.06)], cardiac disorders [case numbers: 731, ROR (95% CI) = 3.29 (3.05–3.54), PRR (95% CI) = 3.14 (2.93–3.37), IC (IC025) = 1.65 (1.54), and EBGM (EBGM05) = 3.14 (2.91)], and neoplasms benign, malignant, and unspecified (including cysts and polyps) [case numbers: 564, ROR (95% CI) = 3.58 (3.29–3.90), PRR (95% CI) = 3.46 (3.19–3.75), IC (IC025) = 1.79 (1.66), and EBGM (EBGM05) = 3.46 (3.17)]. The epirubicin label includes a list of these items. However, the medication label for epirubicin does not include all SOC keywords, such as hepatobiliary illnesses, pregnancy, puerperium, perinatal ailments, and congenital, familial, and genetic disorders.

**TABLE 2:** The signal strength of AEs of Epirubicin at the SOC level in the FAERS database.

SOC Name	SOC Code	Case Numbers	ROR (95% CI)	PRR (95% CI)	Chi square	IC (IC025)	EBGM (EBGM05)
Blood and lymphatic system disorders	10005329	1011	7.00 (6.56-7.47)	6.47 (6.10-6.87)	4732.73	2.69 (2.59)	6.46 (6.06)
Cardiac disorders*	10007541	731	3.29 (3.05-3.54)	3.14 (2.93-3.37)	1089.17	1.65 (1.54)	3.14 (2.91)
Neoplasms benign, malignant and unspecified (incl cysts and polyps)	10029104	564	3.58 (3.29-3.90)	3.46 (3.19-3.75)	998.42	1.79 (1.66)	3.46 (3.17)
Hepatobiliary disorders	10019805	506	7.27 (6.65-7.95)	7.00 (6.42-7.62)	2610.84	2.80 (2.66)	6.98 (6.39)
Pregnancy, puerperium and perinatal conditions	10036585	87	4.71 (3.81-5.82)	4.68 (3.80-5.77)	252.03	2.23 (1.86)	4.68 (3.79)
Congenital, familial and genetic disorders	10010331	33	6.37 (4.53-8.97)	6.36 (4.52-8.94)	148.68	2.67 (1.96)	6.34 (4.51)

### Signal detections ranked by the EBGM at the prefer term level

In our study, four algorithms were used to analyze drug reactions and evaluate their compliance with various screening criteria. The signals identified by the four algorithms are listed in Table 3 and Supplementary Table S1. At the PT level, 253 ADRs were associated with 25 SOCs. As shown in Table 3, the top five strongest signal ADRs, ranked by the EBGM algorithm at the PT level—the most stringent algorithm—were hepatic artery stenosis [case numbers: 8, EBGM: 468.01 (219.99)], endocardial fibrosis [case numbers: 3, EBGM: 426.22 (125.53)], gait apraxia [case numbers: 3, EBGM: 426.22 (125.53)], cardiac perfusion defect [case numbers: 3, EBGM: 331.50 (99.81)], and hepatic artery occlusion [Case numbers: 3, EBGM: 308.64 (93.40)].

**TABLE 3:** The top 30 signal strength of AEs of Epirubicin ranked by EBGM at the PTs level in the FAERS database.

PTs	SOC	Case Numbers	EBGM (EBGM05)
Hepatic artery stenosis	Hepatobiliary disorders	8	468.01 (219.99)
Endocardial fibrosis	Cardiac disorders	3	426.22 (125.53)
Gait apraxia	Nervous system disorders	3	426.22 (125.53)
Cardiac perfusion defect	Cardiac disorders	3	331.5 (99.81)
Hepatic artery occlusion	Hepatobiliary disorders	3	308.64 (93.4)
Administration site oedema	General disorders and administration site conditions	4	284.15 (101.4)
Post embolisation syndrome	Injury, poisoning and procedural complications	4	195.64 (70.97)
Menopausal disorder	Reproductive system and breast disorders	3	142.07 (44.55)
Granulocyte count decreased	Investigations	26	138.27 (93.24)
Intestinal atresia	Congenital, familial and genetic disorders	3	127.87 (40.21)
Biliary fistula	Hepatobiliary disorders	3	119.34 (37.6)
Acute cutaneous lupus erythematosus	Skin and subcutaneous tissue disorders	4	110.5 (40.7)
Miller Fisher syndrome	Nervous system disorders	4	102 (37.62)
Right atrial dilatation	Cardiac disorders	7	97.59 (45.94)
Administration site extravasation	General disorders and administration site conditions	19	86.02 (54.49)
Xerophthalmia	Eye disorders	8	83.16 (41.17)
Placental disorder	Pregnancy, puerperium and perinatal conditions	10	76.11 (40.61)
Neutrophil percentage decreased	Investigations	4	70.2 (26.04)
Cardiac dysfunction	Cardiac disorders	49	68.67 (51.7)
Bladder irritation	Renal and urinary disorders	8	62.81 (31.17)
Refractory cancer	Neoplasms benign, malignant and unspecified (incl cysts and polyps)	3	62.59 (19.94)
Metastases to thorax	Neoplasms benign, malignant and unspecified (incl cysts and polyps)	3	59.28 (18.9)
Pseudocirrhosis	Hepatobiliary disorders	4	53.52 (19.9)
Merycism	Psychiatric disorders	3	52.96 (16.91)
Dilated cardiomyopathy	Cardiac disorders	43	50.79 (37.55)
Catheter site related reaction	General disorders and administration site conditions	5	49.4 (20.41)
Metastases to the mediastinum	Neoplasms benign, malignant and unspecified (incl cysts and polyps)	4	48.71 (18.13)
Subclavian vein thrombosis	Vascular disorders	8	44.45 (22.11)
Carbohydrate antigen 15-3 increased	Investigations	6	43.03 (19.22)
Psychotic behaviour	Psychiatric disorders	7	42.45 (20.12)

Hepatic artery stenosis and occlusion are associated with hepatobiliary disorders, whereas gait apraxia is associated with nervous system disorders. To our knowledge, neither hepatobiliary disorders nor nervous system disorders are included in the epirubicin label; therefore, they could be identified as potential new ADRs of epirubicin. Moreover, several newly detected ADRs have been found to be associated with other SOCs such as intestinal atresia, Miller Fisher syndrome, and merycism. Please refer to Supplementary Table S1 for the EBGM rankings of other PTs. Additionally, 390 ADRs were identified by ROR and PRR, and 348 ADRs were identified by ROR, PRR, and BCPNN (Supplementary Tables S1–S5).

### Other drugs and gait apraxia

The occurrence of gait apraxia, a neurological disorder, suggests that epirubicin may damage the nervous system; this attracted our attention. To further explain the conclusions of our study, we reviewed the FAERS database to identify other drugs that may cause gait apraxia. Medical records submitted between the first quarter of 2014 and the first quarter of 2023 of patients who developed adverse events of gait apraxia were collected. The medications used in these patients were also collected. We found that 17 drugs, including epirubicin, gabapentin, and imatinib, were reported to be associated with gait apraxia (Supplementary Table S6). Carbidopa\levodopa contains two ingredients, so it was excluded. We further conducted ADR analyses of the 16 drug types and found that gabapentin could cause gait apraxia [case numbers: 3, ROR: 35.32 (10.54), PRR: 35.22 (10.54), BCPNN: 4.50 (3.41), and EBGM: 162.03 (38.72)].

## Discussion

Despite being approved by the FDA, serious but less-common safety problems can emerge once the drug is post-marketed (Garashi et al., 2022; Kong et al., 2023). This phenomenon may be partly caused by: i) a larger patient population, making the detection of rare AEs more feasible; ii) a wider range of patients with underlying diseases and co-medications, making it easier to detect interactions between drugs and diseases, as well as drugs and drugs; iii) drug abuse; and iv) drug misuse (Schick et al., 2017). Given these problems, the public FAERS database could help investigation into less common adverse drug reactions and provide guidance for clinical drug use. To our knowledge, this is the first study to investigate epirubicin-related AEs using real-world data from the FAERS database.

Our study analyzed the signal strength of AEs ranked by case number, the three most frequent of which were blood and lymphatic system disorders, cardiac disorders, and neoplasms benign, malignant, and unspecified (including cysts and polyps) consistent with the drug instructions (Schneeweiss et al., 2018). In addition, the number of AEs associated with hepatobiliary disorders and the signal intensity were both high, indicating that these disorders were closely related to epirubicin.

Among the top five results generated from all four algorithms and ranked by the value of the EBGM, which is a stringent algorithm, we found that hepatic artery stenosis and hepatic artery occlusion were associated with hepatobiliary disorders, and gait apraxia was associated with nervous system disorders. Neither type of disorder was listed on the epirubicin label.

Hepatic artery stenosis and occlusion may be partly due to transarterial chemotherapy (Matsui et al., 2017a; Matsui et al., 2017b). Hepatocellular carcinoma (HCC), one of the most common cancers worldwide, has left many people annually facing the threat of death (Okuda et al., 1985; Bosch et al., 2004). Hepatic artery damage (HAD) associated with transcatheter arterial chemoembolization (TACE) (Idée and Guiu, 2013) may affect clinical treatment outcomes. A previous study comprehensively analyzed the occurrence of HAD in 33 patients treated with TACE alone using a mixture of epirubicin, iodized oil, and gelatin sponge (Maeda et al., 2008). The results of this study indicated that accumulated epirubicin dose was significantly associated with HAD (*p* = 0.001). Our study also suggests that hepatic artery stenosis and occlusion are potential side effects of epirubicin. Therefore, during the course of clinical use, more attention should be paid to liver artery damage caused by epirubicin.

Other drugs associated with gait apraxia were also investigated. Gabapentin, an anticonvulsant, has been widely reported to be associated with gait apraxia (Meng et al., 2014; Wiffen et al., 2017). Based on the four algorithms used in our study, we also identified that gabapentin can cause gait apraxia, which is consistent with previous studies, indicating the high robustness and reliability of the four algorithms. We also found that the patient IDs reported for other drugs were inconsistent with those reported for epirubicin, indicating that gait apraxia caused by epirubicin was not caused by other drug ingredients.

Gait apraxia has been widely used to describe gait disturbances accompanied by normal-pressure hydrocephalus, cerebral small vessel disease, and other frontal disorders (Dale et al., 2018). Gait apraxia, a nervous system disease, may indicate that epirubicin has damaged the nervous system. Gait apraxia is also referred to as a gait disturbance and is associated with many signs, including locomotor abnormalities, disequilibrium, loss of gait ignition, and inappropriate postural responses (Della Sala et al., 2002). It is widely accepted that injury to the frontal lobe and cerebellum and damage to the connections between the frontal lobe, basal ganglia, cerebellum, and brainstem may be the cause of gait apraxia (Thompson, 2012). Since its approval for cancer treatment, epirubicin-induced cerebral lesions have attracted increasing attention. Although the blood–brain barrier restricts many therapeutic molecules from entering the brain, chemotherapy, including epirubicin therapy, can damage the microscopic structure of the cerebral tissue through direct neurotoxicity (Meyers, 2008). The following reports support this hypothesis. Chen et al. (2023) found that, compared with breast cancer survivors who did not receive chemotherapy (docetaxel and epirubicin), survivors who received chemotherapy had reduced white matter integrity in the middle frontal gyrus (MFG). Deprez et al. (2012) found that, compared with controls, patients with breast cancer who were exposed to chemotherapy (epirubicin, fluorouracil, and cyclophosphamide) had significant decreases in fractional anisotropy (FA) in the frontal WM tracts after treatment, suggesting poor connectivity of the brain microstructure. Considering the important role of the frontal lobe and the connections between it, the basal ganglia, and the cerebellum, it is reasonable to conclude that epirubicin may cause gait apraxia.

To date, we have not found studies that have shown that gait apraxia is associated with the use of epirubicin, which seems to indicate that there is only a correlation, not a causation. However, we still pay more attention to the occurrence of gait apraxia associated with the clinical use of epirubicin.

It is worth emphasizing that we found many ADRs that have not been listed on the drug label, such as intestinal atresia and Miller Fisher syndrome, which should be paid closer attention in future clinical drug use.

This study has some limitations that should be considered. First, although ROR and PRR have high stability and specificity, BCPNN has higher sensitivity, stability, and specificity than ROR and PRR (Zhang et al., 2023). To make our results more robust, we used four algorithms to filter the AEs associated with the use of epirubicin: PRR, ROR, BCPNN, and EBGM. AEs were considered to have occurred only when all four algorithms met simultaneously, thereby guaranteeing the accuracy of their detection. Second, owing to the small number of patients treated with epirubicin, we could not determine the incidence of adverse reactions. Third, because the sources of reports in the FAERS are diverse, there are differences in the quality of data submitted by reporters, leading to omissions and errors. Fourth, we could not completely avoid the occurrence of false-positive signals, although four algorithms were utilized. Moreover, each patient’s additional prescription drugs may not be fully disclosed in their medical records. It is therefore difficult to determine whether an adverse reaction was caused by the target drug, a companion drug, or a combination of the two. Therefore, more effort should be made to address these limitations and investigate the safety of epirubicin.

## Conclusion

The findings of this study provide several insights into the clinical applications of epirubicin. Several unexpected AEs such as hepatic artery stenosis, gait apraxia, and intestinal atresia are associated with the use of epirubicin. Clinicians should be careful when prescribing epirubicin to patients to prevent the emergence of these AEs. Further clinical studies are required to validate these findings. To better protect patients, the safety of epirubicin must be continuously explored in the real world.

## Data Availability

The data analyzed in this study were obtained from the U.S. Food and Drug Administration (FDA) Adverse Event Reporting System (FAERS: https://www.fda.gov/drugs/surveillance/questions-and-answers-fdas-adverse-event-reporting-system-faers). The following licenses/restrictions apply. Users of these files need to be familiar with creation of relational databases using applications such as ORACLE\xAE, Microsoft Office Access, MySQL\xAE, and IBM DB2, or the use of ASCII files with SAS\xAE analytic tools. A simple search of FAERS data cannot be performed with these files by those unfamiliar with the creation of relational databases. However, a summary FAERS report for a product can be obtained by sending a Freedom of Information Act (FOIA) request to the FDA. You can also request individual case reports by submitting a FOIA request listing the case report number(s). Requests to access these datasets should be directed to the FDA: https://fis.fda.gov/extensions/FPD-QDE-FAERS/FPD-QDE-FAERS.html.
