# Supplementary material for: Epirubicin and gait apraxia: a real-world data analysis of the FDA Adverse Event Reporting System database
Source: Front Pharmacol. 2023 Sep 14;14:1249845. doi: 10.3389/fphar.2023.1249845 (PMC10536159; doi:10.3389/fphar.2023.1249845)
Supplement: Supplementary file 1 [file Table1.docx]

Supplementary Table S1 The comprehensive detailed information of all AEs at the PTs level identified by all the four algorithms simultaneously, and ranked by the value of EBGM.

| Preferred Terms | SOC | Case Numbers | EBGM (EBGM05) |
| --- | --- | --- | --- |
| Hepatic artery stenosis | Hepatobiliary disorders | 8 | 468.01 (219.99) |
| Endocardial fibrosis | Cardiac disorders | 3 | 426.22 (125.53) |
| Gait apraxia | Nervous system disorders | 3 | 426.22 (125.53) |
| Cardiac perfusion defect | Cardiac disorders | 3 | 331.5 (99.81) |
| Hepatic artery occlusion | Hepatobiliary disorders | 3 | 308.64 (93.4) |
| Administration site oedema | General disorders and administration site conditions | 4 | 284.15 (101.4) |
| Post embolisation syndrome | Injury, poisoning and procedural complications | 4 | 195.64 (70.97) |
| Menopausal disorder | Reproductive system and breast disorders | 3 | 142.07 (44.55) |
| Granulocyte count decreased | Investigations | 26 | 138.27 (93.24) |
| Intestinal atresia | Congenital, familial and genetic disorders | 3 | 127.87 (40.21) |
| Biliary fistula | Hepatobiliary disorders | 3 | 119.34 (37.6) |
| Acute cutaneous lupus erythematosus | Skin and subcutaneous tissue disorders | 4 | 110.5 (40.7) |
| Miller Fisher syndrome | Nervous system disorders | 4 | 102 (37.62) |
| Right atrial dilatation | Cardiac disorders | 7 | 97.59 (45.94) |
| Administration site extravasation | General disorders and administration site conditions | 19 | 86.02 (54.49) |
| Xerophthalmia | Eye disorders | 8 | 83.16 (41.17) |
| Placental disorder | Pregnancy, puerperium and perinatal conditions | 10 | 76.11 (40.61) |
| Neutrophil percentage decreased | Investigations | 4 | 70.2 (26.04) |
| Cardiac dysfunction | Cardiac disorders | 49 | 68.67 (51.7) |
| Bladder irritation | Renal and urinary disorders | 8 | 62.81 (31.17) |
| Refractory cancer | Neoplasms benign, malignant and unspecified (incl cysts and polyps) | 3 | 62.59 (19.94) |
| Metastases to thorax | Neoplasms benign, malignant and unspecified (incl cysts and polyps) | 3 | 59.28 (18.9) |
| Pseudocirrhosis | Hepatobiliary disorders | 4 | 53.52 (19.9) |
| Merycism | Psychiatric disorders | 3 | 52.96 (16.91) |
| Dilated cardiomyopathy | Cardiac disorders | 43 | 50.79 (37.55) |
| Catheter site related reaction | General disorders and administration site conditions | 5 | 49.4 (20.41) |
| Metastases to the mediastinum | Neoplasms benign, malignant and unspecified (incl cysts and polyps) | 4 | 48.71 (18.13) |
| Subclavian vein thrombosis | Vascular disorders | 8 | 44.45 (22.11) |
| Carbohydrate antigen 15-3 increased | Investigations | 6 | 43.03 (19.22) |
| Psychotic behaviour | Psychiatric disorders | 7 | 42.45 (20.12) |
| Neutropenic sepsis | Infections and infestations | 55 | 42.23 (32.34) |
| Breast cancer recurrent | Neoplasms benign, malignant and unspecified (incl cysts and polyps) | 22 | 41.99 (27.56) |
| Myocardial oedema | Cardiac disorders | 3 | 41.83 (13.38) |
| Soft tissue sarcoma | Neoplasms benign, malignant and unspecified (incl cysts and polyps) | 4 | 41.73 (15.55) |
| Allodynia | Nervous system disorders | 5 | 37.86 (15.67) |
| Soft tissue necrosis | Musculoskeletal and connective tissue disorders | 4 | 36.5 (13.61) |
| Myocardial fibrosis | Cardiac disorders | 5 | 35.35 (14.63) |
| Aortic dilatation | Vascular disorders | 6 | 34.9 (15.6) |
| Hypercreatininaemia | Metabolism and nutrition disorders | 3 | 34.83 (11.16) |
| Acute leukaemia | Neoplasms benign, malignant and unspecified (incl cysts and polyps) | 8 | 33.48 (16.67) |
| Amnestic disorder | Nervous system disorders | 3 | 33.03 (10.58) |
| Arterial thrombosis | Vascular disorders | 10 | 32.5 (17.42) |
| Myelosuppression | Blood and lymphatic system disorders | 121 | 31.78 (26.54) |
| Tongue coated | Gastrointestinal disorders | 8 | 31.74 (15.81) |
| Hyperamylasaemia | Metabolism and nutrition disorders | 3 | 31.63 (10.14) |
| Cardiotoxicity | Cardiac disorders | 53 | 31.49 (24.01) |
| Aortitis | Vascular disorders | 4 | 30.37 (11.34) |
| Hyperlipasaemia | Metabolism and nutrition disorders | 3 | 28.41 (9.11) |
| Aortic thrombosis | Vascular disorders | 5 | 27.68 (11.47) |
| Abdominal sepsis | Infections and infestations | 4 | 27.31 (10.2) |
| Hepatic function abnormal | Hepatobiliary disorders | 161 | 26.94 (23.04) |
| Anastomotic leak | Injury, poisoning and procedural complications | 3 | 26.64 (8.55) |
| Iatrogenic injury | Injury, poisoning and procedural complications | 3 | 26.33 (8.45) |
| Dilatation ventricular | Cardiac disorders | 5 | 25.9 (10.74) |
| Febrile bone marrow aplasia | Blood and lymphatic system disorders | 20 | 25.57 (16.46) |
| Subacute cutaneous lupus erythematosus | Skin and subcutaneous tissue disorders | 10 | 24.66 (13.23) |
| Coronary artery thrombosis | Cardiac disorders | 6 | 24.62 (11.02) |
| Bone marrow failure | Blood and lymphatic system disorders | 105 | 24.55 (20.24) |
| Troponin T increased | Investigations | 5 | 23.57 (9.77) |
| Neutropenic infection | Infections and infestations | 3 | 22.89 (7.35) |
| Troponin I increased | Investigations | 6 | 22.49 (10.07) |
| Hydrothorax | Respiratory, thoracic and mediastinal disorders | 4 | 22.35 (8.36) |
| Extravasation | General disorders and administration site conditions | 16 | 22.27 (13.61) |
| Second primary malignancy | Neoplasms benign, malignant and unspecified (incl cysts and polyps) | 43 | 21.9 (16.21) |
| Ventricular hypokinesia | Cardiac disorders | 11 | 21.89 (12.09) |
| N-terminal prohormone brain natriuretic peptide increased | Investigations | 6 | 21.29 (9.53) |
| Agranulocytosis | Blood and lymphatic system disorders | 63 | 20.87 (16.28) |
| Cardiac death | General disorders and administration site conditions | 4 | 20.76 (7.76) |
| Ventricular dysfunction | Cardiac disorders | 5 | 20.63 (8.56) |
| Anal ulcer | Gastrointestinal disorders | 3 | 20.44 (6.56) |
| Bile duct stenosis | Hepatobiliary disorders | 4 | 20.09 (7.51) |
| Acute promyelocytic leukaemia | Neoplasms benign, malignant and unspecified (incl cysts and polyps) | 3 | 19.89 (6.39) |
| Cutaneous symptom | Skin and subcutaneous tissue disorders | 3 | 19.8 (6.36) |
| Hypertransaminasaemia | Hepatobiliary disorders | 21 | 19.67 (12.8) |
| Hepatitis B | Infections and infestations | 19 | 19.04 (12.12) |
| Menopause | Social circumstances | 8 | 18.93 (9.44) |
| Ocular toxicity | Eye disorders | 3 | 18.88 (6.07) |
| Neutropenic colitis | Gastrointestinal disorders | 7 | 18.55 (8.82) |
| Atypical haemolytic uraemic syndrome | Blood and lymphatic system disorders | 3 | 18.45 (5.93) |
| Infusion site discolouration | General disorders and administration site conditions | 3 | 18.42 (5.92) |
| Jugular vein thrombosis | Vascular disorders | 5 | 18.26 (7.58) |
| Kidney enlargement | Renal and urinary disorders | 3 | 17.41 (5.6) |
| Left ventricular dysfunction | Cardiac disorders | 20 | 17.07 (11) |
| Hepatic lesion | Hepatobiliary disorders | 13 | 16.99 (9.85) |
| Liver injury | Hepatobiliary disorders | 61 | 16.48 (12.8) |
| Mental fatigue | Psychiatric disorders | 5 | 16.36 (6.79) |
| Axillary mass | Musculoskeletal and connective tissue disorders | 3 | 15.65 (5.03) |
| Respiratory alkalosis | Respiratory, thoracic and mediastinal disorders | 4 | 15.54 (5.82) |
| Lymphangiosis carcinomatosa | Neoplasms benign, malignant and unspecified (incl cysts and polyps) | 3 | 15.46 (4.97) |
| Biliary tract disorder | Hepatobiliary disorders | 3 | 15.09 (4.85) |
| Acute pulmonary oedema | Respiratory, thoracic and mediastinal disorders | 13 | 15.09 (8.75) |
| Dysentery | Infections and infestations | 6 | 14.84 (6.65) |
| Pancreatitis necrotising | Gastrointestinal disorders | 5 | 14.83 (6.16) |
| Diastolic dysfunction | Cardiac disorders | 7 | 14.74 (7.01) |
| Right ventricular dysfunction | Cardiac disorders | 3 | 14.53 (4.67) |
| Acute lymphocytic leukaemia | Neoplasms benign, malignant and unspecified (incl cysts and polyps) | 8 | 14.53 (7.25) |
| Metastases to lymph nodes | Neoplasms benign, malignant and unspecified (incl cysts and polyps) | 17 | 14.35 (8.91) |
| Adenocarcinoma gastric | Neoplasms benign, malignant and unspecified (incl cysts and polyps) | 3 | 14.12 (4.54) |
| Catheter site pain | General disorders and administration site conditions | 7 | 13.87 (6.6) |
| Cardiomyopathy | Cardiac disorders | 32 | 13.78 (9.73) |
| Metastasis | Neoplasms benign, malignant and unspecified (incl cysts and polyps) | 17 | 13.63 (8.46) |
| Biliary dilatation | Hepatobiliary disorders | 3 | 13.56 (4.36) |
| Dislocation of vertebra | Injury, poisoning and procedural complications | 3 | 13.42 (4.32) |
| Fanconi syndrome acquired | Renal and urinary disorders | 5 | 13.17 (5.47) |
| Hyperammonaemic encephalopathy | Nervous system disorders | 5 | 13.07 (5.43) |
| Menopausal symptoms | Reproductive system and breast disorders | 5 | 13.04 (5.42) |
| Ejection fraction decreased | Investigations | 36 | 12.93 (9.31) |
| Pharyngeal erythema | Respiratory, thoracic and mediastinal disorders | 5 | 12.88 (5.35) |
| Angiopathy | Vascular disorders | 11 | 12.87 (7.12) |
| Drug-induced liver injury | Hepatobiliary disorders | 75 | 12.78 (10.18) |
| Phlebitis | Vascular disorders | 10 | 12.78 (6.86) |
| Breast cancer metastatic | Neoplasms benign, malignant and unspecified (incl cysts and polyps) | 20 | 12.77 (8.23) |
| Skin hypopigmentation | Skin and subcutaneous tissue disorders | 5 | 12.62 (5.24) |
| Myocardial injury | Cardiac disorders | 4 | 12.44 (4.66) |
| Appendicitis perforated | Infections and infestations | 5 | 12.4 (5.15) |
| Skin sensitisation | Skin and subcutaneous tissue disorders | 3 | 12.31 (3.96) |
| Vena cava thrombosis | Vascular disorders | 3 | 12.23 (3.93) |
| Cardiac failure chronic | Cardiac disorders | 11 | 12.22 (6.76) |
| Adenocarcinoma | Neoplasms benign, malignant and unspecified (incl cysts and polyps) | 5 | 12.13 (5.04) |
| Hyperpyrexia | General disorders and administration site conditions | 8 | 12.02 (6) |
| Neutrophil count decreased | Investigations | 86 | 11.96 (9.67) |
| Multi-organ disorder | General disorders and administration site conditions | 3 | 11.9 (3.83) |
| Mucosal inflammation | General disorders and administration site conditions | 54 | 11.77 (9.01) |
| Body temperature abnormal | Investigations | 5 | 11.6 (4.82) |
| Odynophagia | Gastrointestinal disorders | 11 | 11.5 (6.36) |
| Mucosal dryness | General disorders and administration site conditions | 4 | 11.3 (4.23) |
| Catheter site erythema | General disorders and administration site conditions | 5 | 11.22 (4.66) |
| Metastases to bone | Neoplasms benign, malignant and unspecified (incl cysts and polyps) | 33 | 11.17 (7.93) |
| Mucosal disorder | General disorders and administration site conditions | 4 | 11.02 (4.13) |
| Acute myeloid leukaemia | Neoplasms benign, malignant and unspecified (incl cysts and polyps) | 29 | 10.99 (7.63) |
| Tetany | Metabolism and nutrition disorders | 3 | 10.88 (3.5) |
| Peripheral sensory neuropathy | Nervous system disorders | 11 | 10.73 (5.93) |
| Pseudomonal sepsis | Infections and infestations | 3 | 10.71 (3.45) |
| Maternal exposure timing unspecified | Injury, poisoning and procedural complications | 5 | 10.69 (4.44) |
| Cardiac failure acute | Cardiac disorders | 13 | 10.61 (6.15) |
| Nail infection | Infections and infestations | 3 | 10.54 (3.39) |
| Febrile neutropenia | Blood and lymphatic system disorders | 122 | 10.45 (8.74) |
| Neoplasm recurrence | Neoplasms benign, malignant and unspecified (incl cysts and polyps) | 6 | 10.4 (4.66) |
| Liver abscess | Infections and infestations | 6 | 10.36 (4.65) |
| Metastases to meninges | Neoplasms benign, malignant and unspecified (incl cysts and polyps) | 4 | 10.12 (3.79) |
| Malignant pleural effusion | Neoplasms benign, malignant and unspecified (incl cysts and polyps) | 3 | 10.08 (3.24) |
| Livedo reticularis | Skin and subcutaneous tissue disorders | 3 | 9.91 (3.19) |
| Mitral valve incompetence | Cardiac disorders | 14 | 9.82 (5.81) |
| Injection site hypersensitivity | General disorders and administration site conditions | 5 | 9.65 (4.01) |
| Intervertebral discitis | Infections and infestations | 3 | 9.63 (3.1) |
| Periodontitis | Infections and infestations | 3 | 9.61 (3.09) |
| Tricuspid valve incompetence | Cardiac disorders | 9 | 9.6 (4.99) |
| Polyneuropathy | Nervous system disorders | 21 | 9.6 (6.25) |
| Radiotherapy | Surgical and medical procedures | 3 | 9.42 (3.03) |
| Skin toxicity | Skin and subcutaneous tissue disorders | 9 | 9.33 (4.85) |
| Portal vein thrombosis | Hepatobiliary disorders | 5 | 9.24 (3.84) |
| Cerebral venous thrombosis | Nervous system disorders | 3 | 9.03 (2.91) |
| Venous thrombosis | Vascular disorders | 6 | 9.03 (4.05) |
| Lichenoid keratosis | Skin and subcutaneous tissue disorders | 3 | 8.95 (2.88) |
| Jaundice neonatal | Pregnancy, puerperium and perinatal conditions | 3 | 8.93 (2.88) |
| Gastrointestinal necrosis | Gastrointestinal disorders | 4 | 8.86 (3.32) |
| Lymphadenopathy mediastinal | Blood and lymphatic system disorders | 3 | 8.84 (2.85) |
| Sinus headache | Nervous system disorders | 8 | 8.84 (4.42) |
| Metastases to lung | Neoplasms benign, malignant and unspecified (incl cysts and polyps) | 19 | 8.79 (5.6) |
| Thrombocytosis | Blood and lymphatic system disorders | 6 | 8.77 (3.93) |
| Myocardial necrosis marker increased | Investigations | 3 | 8.63 (2.78) |
| Infusion site extravasation | General disorders and administration site conditions | 12 | 8.58 (4.87) |
| Muscle contracture | Musculoskeletal and connective tissue disorders | 3 | 8.52 (2.74) |
| Multiple-drug resistance | General disorders and administration site conditions | 5 | 8.38 (3.48) |
| Gingivitis | Infections and infestations | 8 | 8.34 (4.17) |
| Leukopenia | Blood and lymphatic system disorders | 70 | 8.21 (6.49) |
| Normochromic normocytic anaemia | Blood and lymphatic system disorders | 3 | 8.2 (2.64) |
| Pleuritic pain | Respiratory, thoracic and mediastinal disorders | 3 | 8.15 (2.62) |
| Neoplasm progression | Neoplasms benign, malignant and unspecified (incl cysts and polyps) | 64 | 8.01 (6.27) |
| Skin necrosis | Skin and subcutaneous tissue disorders | 7 | 7.95 (3.78) |
| Palmar-plantar erythrodysaesthesia syndrome | Skin and subcutaneous tissue disorders | 35 | 7.77 (5.58) |
| Aphthous ulcer | Gastrointestinal disorders | 14 | 7.65 (4.52) |
| White blood cell count decreased | Investigations | 158 | 7.64 (6.53) |
| Sputum discoloured | Respiratory, thoracic and mediastinal disorders | 15 | 7.61 (4.59) |
| Cardiac failure | Cardiac disorders | 109 | 7.6 (6.29) |
| Hepatitis B reactivation | Infections and infestations | 6 | 7.56 (3.39) |
| Neutropenia | Blood and lymphatic system disorders | 189 | 7.54 (6.53) |
| Intercepted product administration error | Injury, poisoning and procedural complications | 5 | 7.41 (3.08) |
| Iron deficiency | Metabolism and nutrition disorders | 6 | 7.38 (3.31) |
| Gastrointestinal toxicity | Gastrointestinal disorders | 6 | 7.26 (3.26) |
| Electrolyte imbalance | Metabolism and nutrition disorders | 14 | 7.11 (4.21) |
| Cytopenia | Blood and lymphatic system disorders | 17 | 7.09 (4.4) |
| Dermatomyositis | Skin and subcutaneous tissue disorders | 3 | 7.08 (2.28) |
| Premature labour | Pregnancy, puerperium and perinatal conditions | 6 | 7.05 (3.16) |
| Kounis syndrome | Cardiac disorders | 3 | 7.01 (2.26) |
| Spinal cord compression | Nervous system disorders | 5 | 6.85 (2.85) |
| Aplasia | Congenital, familial and genetic disorders | 3 | 6.84 (2.2) |
| Renal tubular disorder | Renal and urinary disorders | 3 | 6.82 (2.2) |
| Fibrin D dimer increased | Investigations | 4 | 6.81 (2.55) |
| Cachexia | Metabolism and nutrition disorders | 6 | 6.79 (3.05) |
| Haematotoxicity | Blood and lymphatic system disorders | 11 | 6.78 (3.75) |
| Laryngospasm | Respiratory, thoracic and mediastinal disorders | 3 | 6.77 (2.18) |
| Erythema nodosum | Skin and subcutaneous tissue disorders | 4 | 6.75 (2.53) |
| Disease recurrence | General disorders and administration site conditions | 59 | 6.72 (5.2) |
| Oligohydramnios | Pregnancy, puerperium and perinatal conditions | 4 | 6.66 (2.49) |
| Granulocytopenia | Blood and lymphatic system disorders | 5 | 6.65 (2.77) |
| Oral pain | Gastrointestinal disorders | 29 | 6.65 (4.61) |
| Atypical pneumonia | Infections and infestations | 4 | 6.53 (2.45) |
| Appetite disorder | Metabolism and nutrition disorders | 8 | 6.53 (3.26) |
| Radiation pneumonitis | Injury, poisoning and procedural complications | 3 | 6.48 (2.09) |
| Abdominal adhesions | Gastrointestinal disorders | 4 | 6.46 (2.42) |
| Faecaloma | Gastrointestinal disorders | 6 | 6.4 (2.87) |
| Bundle branch block left | Cardiac disorders | 4 | 6.25 (2.34) |
| Oliguria | Renal and urinary disorders | 6 | 6.15 (2.76) |
| Transaminases increased | Investigations | 26 | 6.14 (4.18) |
| Sudden death | General disorders and administration site conditions | 9 | 6.09 (3.17) |
| Retinopathy | Eye disorders | 4 | 6.09 (2.28) |
| Metastases to liver | Neoplasms benign, malignant and unspecified (incl cysts and polyps) | 20 | 6.07 (3.91) |
| Pulmonary function test decreased | Investigations | 6 | 6.01 (2.7) |
| Lymphopenia | Blood and lymphatic system disorders | 16 | 5.96 (3.65) |
| Paranasal sinus discomfort | Respiratory, thoracic and mediastinal disorders | 5 | 5.88 (2.44) |
| Herpes simplex | Infections and infestations | 5 | 5.85 (2.43) |
| Foetal growth restriction | Pregnancy, puerperium and perinatal conditions | 7 | 5.75 (2.74) |
| Blood pressure diastolic increased | Investigations | 5 | 5.63 (2.34) |
| Vein disorder | Vascular disorders | 5 | 5.57 (2.32) |
| Nail disorder | Skin and subcutaneous tissue disorders | 8 | 5.55 (2.77) |
| Neutrophilia | Blood and lymphatic system disorders | 6 | 5.48 (2.46) |
| Interstitial lung disease | Respiratory, thoracic and mediastinal disorders | 46 | 5.46 (4.09) |
| Cystitis haemorrhagic | Renal and urinary disorders | 4 | 5.45 (2.04) |
| Premature baby | Pregnancy, puerperium and perinatal conditions | 32 | 5.4 (3.82) |
| Gamma-glutamyltransferase increased | Investigations | 17 | 5.32 (3.31) |
| Foetal death | Pregnancy, puerperium and perinatal conditions | 6 | 5.28 (2.37) |
| Hepatic failure | Hepatobiliary disorders | 25 | 5.2 (3.51) |
| Pneumocystis jirovecii pneumonia | Infections and infestations | 11 | 5.11 (2.83) |
| Pancreatitis acute | Gastrointestinal disorders | 18 | 5.1 (3.21) |
| Tumour marker increased | Investigations | 5 | 5.07 (2.11) |
| Energy increased | General disorders and administration site conditions | 6 | 5.03 (2.26) |
| Troponin increased | Investigations | 6 | 5.02 (2.25) |
| Incontinence | Renal and urinary disorders | 9 | 4.88 (2.54) |
| Gastroenteritis | Infections and infestations | 12 | 4.87 (2.76) |
| Septic shock | Infections and infestations | 35 | 4.69 (3.36) |
| Hepatocellular injury | Hepatobiliary disorders | 16 | 4.59 (2.81) |
| Neurotoxicity | Nervous system disorders | 15 | 4.55 (2.74) |
| Aspartate aminotransferase increased | Investigations | 33 | 4.54 (3.23) |
| Alanine aminotransferase increased | Investigations | 39 | 4.41 (3.22) |
| Deafness | Ear and labyrinth disorders | 21 | 4.35 (2.84) |
| Disease progression | General disorders and administration site conditions | 87 | 4.19 (3.39) |
| Pancytopenia | Blood and lymphatic system disorders | 38 | 4.17 (3.03) |
| Oral candidiasis | Infections and infestations | 9 | 4.14 (2.15) |
| Metastases to central nervous system | Neoplasms benign, malignant and unspecified (incl cysts and polyps) | 9 | 3.95 (2.05) |
| Neuropathy peripheral | Nervous system disorders | 71 | 3.87 (3.07) |
| Mouth ulceration | Gastrointestinal disorders | 14 | 3.86 (2.28) |
| Hypokalaemia | Metabolism and nutrition disorders | 30 | 3.82 (2.67) |
| Pleural effusion | Respiratory, thoracic and mediastinal disorders | 40 | 3.77 (2.77) |
| Cardiovascular disorder | Cardiac disorders | 10 | 3.77 (2.03) |
| Thrombocytopenia | Blood and lymphatic system disorders | 68 | 3.56 (2.8) |
| Pyrexia | General disorders and administration site conditions | 217 | 3.55 (3.1) |
| Full blood count decreased | Investigations | 15 | 3.49 (2.1) |
| Hyponatraemia | Metabolism and nutrition disorders | 33 | 3.34 (2.37) |
| Asthenia | General disorders and administration site conditions | 222 | 3.24 (2.84) |
| Vomiting | Gastrointestinal disorders | 251 | 3.08 (2.72) |
| Hepatic enzyme increased | Investigations | 34 | 2.94 (2.1) |
| Anaemia | Blood and lymphatic system disorders | 98 | 2.9 (2.38) |
