# Supplementary material for: Epirubicin and gait apraxia: a real-world data analysis of the FDA Adverse Event Reporting System database
Source: Front Pharmacol. 2023 Sep 14;14:1249845. doi: 10.3389/fphar.2023.1249845 (PMC10536159; doi:10.3389/fphar.2023.1249845)
Supplement: Supplementary file 2 [file Table2.docx]

Supplementary Table S2 The comprehensive detailed information of all AEs at the PTs level identified by ROR algorithms.

| Preferred Terms | SOC | Case Number | ROR_95 (95% two side CI) |
| --- | --- | --- | --- |
| Nausea | Gastrointestinal disorders | 300 | 2.11 (1.88-2.36) |
| Fatigue | General disorders and administration site conditions | 184 | 1.21 (1.05-1.4) |
| Diarrhoea | Gastrointestinal disorders | 162 | 1.31 (1.12-1.53) |
| Hepatic artery stenosis | Hepatobiliary disorders | 8 | 555.28 (261.01-1181.29) |
| Endocardial fibrosis | Cardiac disorders | 3 | 497.22 (146.44-1688.24) |
| Gait apraxia | Nervous system disorders | 3 | 497.22 (146.44-1688.24) |
| Alopecia | Skin and subcutaneous tissue disorders | 98 | 2.24 (1.84-2.74) |
| Cardiac perfusion defect | Cardiac disorders | 3 | 372.91 (112.28-1238.6) |
| Decreased appetite | Metabolism and nutrition disorders | 90 | 2.04 (1.65-2.5) |
| Constipation | Gastrointestinal disorders | 76 | 1.9 (1.52-2.39) |
| Hepatic artery occlusion | Hepatobiliary disorders | 3 | 344.23 (104.17-1137.46) |
| Administration site oedema | General disorders and administration site conditions | 4 | 314.06 (112.07-880.1) |
| Paraesthesia | Nervous system disorders | 62 | 2.15 (1.67-2.75) |
| Post embolisation syndrome | Injury, poisoning and procedural complications | 4 | 209.37 (75.96-577.14) |
| Abdominal pain | Gastrointestinal disorders | 58 | 1.41 (1.09-1.83) |
| Chest pain | General disorders and administration site conditions | 51 | 1.67 (1.26-2.19) |
| General physical health deterioration | General disorders and administration site conditions | 49 | 2.52 (1.9-3.33) |
| Myalgia | Musculoskeletal and connective tissue disorders | 47 | 1.57 (1.18-2.09) |
| Malignant neoplasm progression | Neoplasms benign, malignant and unspecified (incl cysts and polyps) | 47 | 2.28 (1.71-3.03) |
| Menopausal disorder | Reproductive system and breast disorders | 3 | 149.17 (46.78-475.68) |
| Maternal exposure during pregnancy | Injury, poisoning and procedural complications | 42 | 2.41 (1.78-3.26) |
| Gastrointestinal disorder | Gastrointestinal disorders | 38 | 2.59 (1.88-3.56) |
| Granulocyte count decreased | Investigations | 26 | 145.27 (97.96-215.43) |
| Chest discomfort | General disorders and administration site conditions | 37 | 2.04 (1.48-2.82) |
| Oropharyngeal pain | Respiratory, thoracic and mediastinal disorders | 37 | 2.05 (1.48-2.83) |
| Intestinal atresia | Congenital, familial and genetic disorders | 3 | 133.58 (42.01-424.76) |
| Breast cancer | Neoplasms benign, malignant and unspecified (incl cysts and polyps) | 35 | 1.62 (1.17-2.26) |
| Biliary fistula | Hepatobiliary disorders | 3 | 124.3 (39.16-394.58) |
| Chills | General disorders and administration site conditions | 35 | 1.68 (1.2-2.34) |
| Sepsis | Infections and infestations | 34 | 1.7 (1.21-2.38) |
| Acute cutaneous lupus erythematosus | Skin and subcutaneous tissue disorders | 4 | 114.75 (42.26-311.57) |
| Palpitations | Cardiac disorders | 33 | 1.59 (1.13-2.24) |
| Dysphagia | Gastrointestinal disorders | 32 | 1.93 (1.36-2.73) |
| Dysgeusia | Nervous system disorders | 31 | 2.31 (1.62-3.29) |
| Platelet count decreased | Investigations | 30 | 1.53 (1.07-2.19) |
| Respiratory failure | Respiratory, thoracic and mediastinal disorders | 30 | 2.44 (1.71-3.5) |
| Miller Fisher syndrome | Nervous system disorders | 4 | 105.61 (38.96-286.33) |
| Right atrial dilatation | Cardiac disorders | 7 | 100.92 (47.51-214.39) |
| Pulmonary embolism | Respiratory, thoracic and mediastinal disorders | 29 | 1.94 (1.35-2.8) |
| Abdominal distension | Gastrointestinal disorders | 28 | 1.5 (1.04-2.17) |
| Atrial fibrillation | Cardiac disorders | 26 | 1.47 (1-2.16) |
| Hot flush | Vascular disorders | 26 | 1.94 (1.32-2.85) |
| Tachycardia | Cardiac disorders | 25 | 1.66 (1.12-2.46) |
| Productive cough | Respiratory, thoracic and mediastinal disorders | 23 | 2.5 (1.66-3.76) |
| Oedema | General disorders and administration site conditions | 23 | 2.51 (1.67-3.78) |
| Disturbance in attention | Nervous system disorders | 22 | 2.26 (1.49-3.44) |
| Discomfort | General disorders and administration site conditions | 22 | 1.93 (1.27-2.94) |
| Deep vein thrombosis | Vascular disorders | 22 | 2.18 (1.43-3.31) |
| Cognitive disorder | Nervous system disorders | 22 | 2.55 (1.68-3.88) |
| Stomatitis | Gastrointestinal disorders | 21 | 1.82 (1.18-2.79) |
| Administration site extravasation | General disorders and administration site conditions | 19 | 88.69 (56.18-140.02) |
| Xerophthalmia | Eye disorders | 8 | 85.58 (42.37-172.87) |
| Cardio-respiratory arrest | Cardiac disorders | 18 | 2.72 (1.71-4.32) |
| Dyspnoea exertional | Respiratory, thoracic and mediastinal disorders | 18 | 2.48 (1.56-3.95) |
| Inflammation | General disorders and administration site conditions | 17 | 1.82 (1.13-2.94) |
| Placental disorder | Pregnancy, puerperium and perinatal conditions | 10 | 78.14 (41.7-146.45) |
| Colitis | Gastrointestinal disorders | 17 | 2.5 (1.55-4.02) |
| Neutrophil percentage decreased | Investigations | 4 | 71.89 (26.66-193.85) |
| Haematuria | Renal and urinary disorders | 16 | 2.42 (1.48-3.95) |
| Metabolic acidosis | Metabolism and nutrition disorders | 16 | 2.86 (1.75-4.67) |
| Cardiac dysfunction | Cardiac disorders | 49 | 70.56 (53.12-93.72) |
| Lower respiratory tract infection | Infections and infestations | 15 | 1.75 (1.05-2.9) |
| Bladder irritation | Renal and urinary disorders | 8 | 64.18 (31.85-129.33) |
| Refractory cancer | Neoplasms benign, malignant and unspecified (incl cysts and polyps) | 3 | 63.93 (20.37-200.65) |
| Blood bilirubin increased | Investigations | 14 | 3.36 (1.99-5.68) |
| Metastases to thorax | Neoplasms benign, malignant and unspecified (incl cysts and polyps) | 3 | 60.47 (19.28-189.68) |
| Hepatotoxicity | Hepatobiliary disorders | 13 | 3.19 (1.85-5.49) |
| Hypophagia | Metabolism and nutrition disorders | 13 | 2.76 (1.6-4.76) |
| Ascites | Gastrointestinal disorders | 13 | 2.5 (1.45-4.3) |
| Glossodynia | Gastrointestinal disorders | 12 | 3.42 (1.94-6.02) |
| C-reactive protein increased | Investigations | 12 | 1.92 (1.09-3.39) |
| Ear pain | Ear and labyrinth disorders | 12 | 3.41 (1.94-6.01) |
| Liver function test abnormal | Investigations | 11 | 2.8 (1.55-5.05) |
| Clostridium difficile infection | Infections and infestations | 11 | 2.3 (1.27-4.16) |
| Acute respiratory failure | Respiratory, thoracic and mediastinal disorders | 11 | 3.1 (1.71-5.59) |
| Red blood cell count decreased | Investigations | 11 | 2.08 (1.15-3.76) |
| Acute respiratory distress syndrome | Respiratory, thoracic and mediastinal disorders | 10 | 3.5 (1.88-6.51) |
| Presyncope | Nervous system disorders | 10 | 2.23 (1.2-4.14) |
| Sensory disturbance | Nervous system disorders | 10 | 3.36 (1.81-6.26) |
| Pseudocirrhosis | Hepatobiliary disorders | 4 | 54.49 (20.27-146.52) |
| Merycism | Psychiatric disorders | 3 | 53.92 (17.21-168.91) |
| Gastritis | Gastrointestinal disorders | 10 | 2.29 (1.23-4.27) |
| Impaired work ability | Social circumstances | 10 | 2.55 (1.37-4.73) |
| Dilated cardiomyopathy | Cardiac disorders | 43 | 51.84 (38.33-70.12) |
| Acute myocardial infarction | Cardiac disorders | 10 | 1.97 (1.06-3.66) |
| Pulmonary hypertension | Respiratory, thoracic and mediastinal disorders | 9 | 2.46 (1.28-4.72) |
| Pulmonary fibrosis | Respiratory, thoracic and mediastinal disorders | 9 | 2.99 (1.55-5.74) |
| Increased appetite | Metabolism and nutrition disorders | 9 | 3.06 (1.59-5.88) |
| Catheter site related reaction | General disorders and administration site conditions | 5 | 50.23 (20.75-121.6) |
| Hepatic steatosis | Hepatobiliary disorders | 9 | 3.09 (1.61-5.95) |
| Photophobia | Eye disorders | 9 | 2.77 (1.44-5.32) |
| Circulatory collapse | Vascular disorders | 9 | 3.28 (1.71-6.31) |
| Haematemesis | Gastrointestinal disorders | 9 | 2.06 (1.07-3.97) |
| Pericardial effusion | Cardiac disorders | 9 | 2.26 (1.17-4.34) |
| Metastases to the mediastinum | Neoplasms benign, malignant and unspecified (incl cysts and polyps) | 4 | 49.52 (18.43-133.04) |
| Jaundice | Hepatobiliary disorders | 9 | 2.17 (1.13-4.17) |
| Subclavian vein thrombosis | Vascular disorders | 8 | 45.14 (22.45-90.75) |
| Carbohydrate antigen 15-3 increased | Investigations | 6 | 43.67 (19.5-97.79) |
| Cardiogenic shock | Cardiac disorders | 8 | 3.44 (1.72-6.89) |
| Joint range of motion decreased | Musculoskeletal and connective tissue disorders | 8 | 3.53 (1.76-7.06) |
| Psychotic behaviour | Psychiatric disorders | 7 | 43.07 (20.42-90.85) |
| Neutropenic sepsis | Infections and infestations | 55 | 43.02 (32.95-56.18) |
| Breast cancer recurrent | Neoplasms benign, malignant and unspecified (incl cysts and polyps) | 22 | 42.66 (27.99-65.01) |
| Myocardial oedema | Cardiac disorders | 3 | 42.42 (13.57-132.6) |
| Soft tissue sarcoma | Neoplasms benign, malignant and unspecified (incl cysts and polyps) | 4 | 42.32 (15.77-113.56) |
| Generalised tonic-clonic seizure | Nervous system disorders | 8 | 2.06 (1.03-4.13) |
| Retching | Gastrointestinal disorders | 8 | 2.09 (1.04-4.17) |
| Quality of life decreased | Investigations | 7 | 3.15 (1.5-6.6) |
| Muscle atrophy | Musculoskeletal and connective tissue disorders | 7 | 3.59 (1.71-7.53) |
| Toxic skin eruption | Skin and subcutaneous tissue disorders | 7 | 4.19 (2-8.8) |
| Paraesthesia oral | Gastrointestinal disorders | 7 | 2.78 (1.32-5.83) |
| Pericarditis | Cardiac disorders | 7 | 3 (1.43-6.3) |
| Allodynia | Nervous system disorders | 5 | 38.35 (15.87-92.68) |
| Acute hepatic failure | Hepatobiliary disorders | 7 | 2.92 (1.39-6.12) |
| Myelodysplastic syndrome | Neoplasms benign, malignant and unspecified (incl cysts and polyps) | 7 | 2.91 (1.39-6.1) |
| Soft tissue necrosis | Musculoskeletal and connective tissue disorders | 4 | 36.95 (13.78-99.06) |
| Blood magnesium decreased | Investigations | 6 | 3.7 (1.66-8.25) |
| Appendicitis | Infections and infestations | 6 | 3.75 (1.68-8.35) |
| Supraventricular tachycardia | Cardiac disorders | 6 | 4 (1.79-8.9) |
| Myocardial fibrosis | Cardiac disorders | 5 | 35.78 (14.81-86.42) |
| Aortic dilatation | Vascular disorders | 6 | 35.31 (15.79-78.99) |
| Hypercreatininaemia | Metabolism and nutrition disorders | 3 | 35.24 (11.29-110) |
| Acute leukaemia | Neoplasms benign, malignant and unspecified (incl cysts and polyps) | 8 | 33.87 (16.87-68) |
| Inappropriate antidiuretic hormone secretion | Endocrine disorders | 6 | 3.56 (1.6-7.92) |
| Infusion site pain | General disorders and administration site conditions | 6 | 2.38 (1.07-5.31) |
| Temperature intolerance | General disorders and administration site conditions | 6 | 3.21 (1.44-7.15) |
| Myocardial ischaemia | Cardiac disorders | 6 | 3.74 (1.68-8.32) |
| Amnestic disorder | Nervous system disorders | 3 | 33.4 (10.7-104.22) |
| Arterial thrombosis | Vascular disorders | 10 | 32.88 (17.62-61.33) |
| Myelosuppression | Blood and lymphatic system disorders | 121 | 32.44 (27.1-38.84) |
| Tongue coated | Gastrointestinal disorders | 8 | 32.09 (15.99-64.43) |
| Hyperbilirubinaemia | Hepatobiliary disorders | 6 | 3.27 (1.47-7.28) |
| Hyperamylasaemia | Metabolism and nutrition disorders | 3 | 31.96 (10.25-99.72) |
| Cardiotoxicity | Cardiac disorders | 53 | 31.95 (24.36-41.91) |
| Tachypnoea | Respiratory, thoracic and mediastinal disorders | 6 | 2.62 (1.17-5.83) |
| Flank pain | Musculoskeletal and connective tissue disorders | 5 | 3.04 (1.26-7.3) |
| Aortitis | Vascular disorders | 4 | 30.68 (11.45-82.17) |
| Intestinal perforation | Gastrointestinal disorders | 5 | 2.58 (1.07-6.19) |
| Tumour lysis syndrome | Metabolism and nutrition disorders | 5 | 3.11 (1.29-7.48) |
| Urosepsis | Infections and infestations | 5 | 2.96 (1.23-7.12) |
| Hyperlipasaemia | Metabolism and nutrition disorders | 3 | 28.69 (9.2-89.44) |
| Sexual dysfunction | Reproductive system and breast disorders | 5 | 2.48 (1.03-5.95) |
| Aortic thrombosis | Vascular disorders | 5 | 27.94 (11.58-67.41) |
| Disseminated intravascular coagulation | Blood and lymphatic system disorders | 5 | 2.44 (1.01-5.86) |
| Oesophagitis | Gastrointestinal disorders | 5 | 3.07 (1.28-7.37) |
| Abdominal sepsis | Infections and infestations | 4 | 27.56 (10.3-73.78) |
| Infusion site erythema | General disorders and administration site conditions | 5 | 3.25 (1.35-7.82) |
| Hepatic function abnormal | Hepatobiliary disorders | 161 | 27.54 (23.56-32.2) |
| Papule | Skin and subcutaneous tissue disorders | 5 | 3.81 (1.58-9.15) |
| Initial insomnia | Psychiatric disorders | 5 | 3.29 (1.37-7.91) |
| Acute coronary syndrome | Cardiac disorders | 5 | 3.52 (1.46-8.45) |
| Anastomotic leak | Injury, poisoning and procedural complications | 3 | 26.88 (8.62-83.77) |
| Iatrogenic injury | Injury, poisoning and procedural complications | 3 | 26.56 (8.52-82.77) |
| Dilatation ventricular | Cardiac disorders | 5 | 26.13 (10.83-63.03) |
| Febrile bone marrow aplasia | Blood and lymphatic system disorders | 20 | 25.82 (16.62-40.11) |
| Cardiomegaly | Cardiac disorders | 5 | 3 (1.25-7.2) |
| Subacute cutaneous lupus erythematosus | Skin and subcutaneous tissue disorders | 10 | 24.88 (13.35-46.36) |
| Coronary artery thrombosis | Cardiac disorders | 6 | 24.83 (11.12-55.47) |
| Bone marrow failure | Blood and lymphatic system disorders | 105 | 24.96 (20.58-30.27) |
| Ovarian cancer | Neoplasms benign, malignant and unspecified (incl cysts and polyps) | 5 | 2.68 (1.11-6.43) |
| Troponin T increased | Investigations | 5 | 23.76 (9.85-57.29) |
| Hyperammonaemia | Metabolism and nutrition disorders | 4 | 4.64 (1.74-12.37) |
| Hepatitis acute | Hepatobiliary disorders | 4 | 3.88 (1.46-10.35) |
| Atrial septal defect | Congenital, familial and genetic disorders | 4 | 2.77 (1.04-7.39) |
| Erythema multiforme | Skin and subcutaneous tissue disorders | 4 | 2.79 (1.05-7.43) |
| Embolism | Vascular disorders | 4 | 2.9 (1.09-7.74) |
| Neutropenic infection | Infections and infestations | 3 | 23.07 (7.41-71.84) |
| Panic reaction | Psychiatric disorders | 4 | 3.11 (1.16-8.28) |
| Rash vesicular | Skin and subcutaneous tissue disorders | 4 | 4.91 (1.84-13.09) |
| Troponin I increased | Investigations | 6 | 22.66 (10.15-50.61) |
| Lymphoedema | Vascular disorders | 4 | 3.14 (1.18-8.36) |
| Hydrothorax | Respiratory, thoracic and mediastinal disorders | 4 | 22.52 (8.42-60.23) |
| Non-Hodgkin's lymphoma | Neoplasms benign, malignant and unspecified (incl cysts and polyps) | 4 | 3.69 (1.38-9.84) |
| Sunburn | Injury, poisoning and procedural complications | 4 | 2.88 (1.08-7.67) |
| Small for dates baby | Pregnancy, puerperium and perinatal conditions | 4 | 4.54 (1.7-12.11) |
| Extravasation | General disorders and administration site conditions | 16 | 22.45 (13.73-36.73) |
| Enteritis | Gastrointestinal disorders | 4 | 3.39 (1.27-9.05) |
| Second primary malignancy | Neoplasms benign, malignant and unspecified (incl cysts and polyps) | 43 | 22.13 (16.39-29.89) |
| Ventricular hypokinesia | Cardiac disorders | 11 | 22.07 (12.19-39.95) |
| Ventricular extrasystoles | Cardiac disorders | 4 | 2.77 (1.04-7.39) |
| Visual field defect | Eye disorders | 4 | 3.36 (1.26-8.96) |
| Pulmonary toxicity | Respiratory, thoracic and mediastinal disorders | 4 | 3.4 (1.27-9.06) |
| Klebsiella infection | Infections and infestations | 4 | 4.55 (1.7-12.12) |
| N-terminal prohormone brain natriuretic peptide increased | Investigations | 6 | 21.44 (9.6-47.88) |
| Agranulocytosis | Blood and lymphatic system disorders | 63 | 21.12 (16.47-27.08) |
| Cholecystitis acute | Hepatobiliary disorders | 4 | 4.96 (1.86-13.23) |
| Cardiac death | General disorders and administration site conditions | 4 | 20.9 (7.82-55.89) |
| Ventricular dysfunction | Cardiac disorders | 5 | 20.78 (8.62-50.08) |
| Anal ulcer | Gastrointestinal disorders | 3 | 20.57 (6.61-64.05) |
| Left ventricular failure | Cardiac disorders | 3 | 5.5 (1.77-17.07) |
| Bile duct stenosis | Hepatobiliary disorders | 4 | 20.23 (7.57-54.08) |
| Acute promyelocytic leukaemia | Neoplasms benign, malignant and unspecified (incl cysts and polyps) | 3 | 20.02 (6.43-62.33) |
| Tongue ulceration | Gastrointestinal disorders | 3 | 4.99 (1.61-15.48) |
| Systemic infection | Infections and infestations | 3 | 6.16 (1.99-19.14) |
| Guillain-Barre syndrome | Nervous system disorders | 3 | 3.83 (1.23-11.88) |
| Cutaneous symptom | Skin and subcutaneous tissue disorders | 3 | 19.93 (6.4-62.05) |
| Hypertransaminasaemia | Hepatobiliary disorders | 21 | 19.83 (12.91-30.47) |
| Organising pneumonia | Respiratory, thoracic and mediastinal disorders | 3 | 3.35 (1.08-10.39) |
| Hypovolaemic shock | Vascular disorders | 3 | 3.47 (1.12-10.78) |
| Ileus paralytic | Gastrointestinal disorders | 3 | 4.65 (1.5-14.44) |
| Blood lactic acid increased | Investigations | 3 | 3.34 (1.08-10.37) |
| Hepatitis B | Infections and infestations | 19 | 19.19 (12.22-30.14) |
| Menopause | Social circumstances | 8 | 19.06 (9.51-38.2) |
| Ocular toxicity | Eye disorders | 3 | 19 (6.11-59.14) |
| Oral fungal infection | Infections and infestations | 3 | 6.16 (1.98-19.12) |
| Neutropenic colitis | Gastrointestinal disorders | 7 | 18.67 (8.88-39.26) |
| Atypical haemolytic uraemic syndrome | Blood and lymphatic system disorders | 3 | 18.57 (5.97-57.78) |
| Infusion site discolouration | General disorders and administration site conditions | 3 | 18.53 (5.95-57.66) |
| Language disorder | Nervous system disorders | 3 | 5.28 (1.7-16.39) |
| Hypertriglyceridaemia | Metabolism and nutrition disorders | 3 | 3.16 (1.02-9.8) |
| Jugular vein thrombosis | Vascular disorders | 5 | 18.37 (7.63-44.27) |
| Polydipsia | Metabolism and nutrition disorders | 3 | 4.8 (1.55-14.91) |
| Arteriosclerosis coronary artery | Cardiac disorders | 3 | 3.42 (1.1-10.62) |
| Aortic valve incompetence | Cardiac disorders | 3 | 5.68 (1.83-17.63) |
| Kidney enlargement | Renal and urinary disorders | 3 | 17.51 (5.63-54.49) |
| Left ventricular dysfunction | Cardiac disorders | 20 | 17.19 (11.07-26.69) |
| Hepatic lesion | Hepatobiliary disorders | 13 | 17.1 (9.91-29.5) |
| Liver injury | Hepatobiliary disorders | 61 | 16.65 (12.93-21.42) |
| Mental fatigue | Psychiatric disorders | 5 | 16.45 (6.83-39.62) |
| Papilloedema | Eye disorders | 3 | 3.94 (1.27-12.21) |
| Epigastric discomfort | Gastrointestinal disorders | 3 | 3.9 (1.26-12.11) |
| Axillary mass | Musculoskeletal and connective tissue disorders | 3 | 15.73 (5.06-48.92) |
| Respiratory alkalosis | Respiratory, thoracic and mediastinal disorders | 4 | 15.62 (5.85-41.73) |
| Lymphangiosis carcinomatosa | Neoplasms benign, malignant and unspecified (incl cysts and polyps) | 3 | 15.54 (5-48.33) |
| Biliary tract disorder | Hepatobiliary disorders | 3 | 15.17 (4.88-47.18) |
| Acute pulmonary oedema | Respiratory, thoracic and mediastinal disorders | 13 | 15.18 (8.8-26.19) |
| Dysentery | Infections and infestations | 6 | 14.92 (6.69-33.28) |
| Pancreatitis necrotising | Gastrointestinal disorders | 5 | 14.9 (6.19-35.89) |
| Diastolic dysfunction | Cardiac disorders | 7 | 14.82 (7.05-31.14) |
| Right ventricular dysfunction | Cardiac disorders | 3 | 14.6 (4.7-45.4) |
| Acute lymphocytic leukaemia | Neoplasms benign, malignant and unspecified (incl cysts and polyps) | 8 | 14.6 (7.29-29.26) |
| Metastases to lymph nodes | Neoplasms benign, malignant and unspecified (incl cysts and polyps) | 17 | 14.43 (8.96-23.25) |
| Adenocarcinoma gastric | Neoplasms benign, malignant and unspecified (incl cysts and polyps) | 3 | 14.18 (4.56-44.1) |
| Catheter site pain | General disorders and administration site conditions | 7 | 13.94 (6.63-29.29) |
| Cardiomyopathy | Cardiac disorders | 32 | 13.87 (9.8-19.64) |
| Metastasis | Neoplasms benign, malignant and unspecified (incl cysts and polyps) | 17 | 13.71 (8.51-22.09) |
| Biliary dilatation | Hepatobiliary disorders | 3 | 13.62 (4.38-42.35) |
| Dislocation of vertebra | Injury, poisoning and procedural complications | 3 | 13.48 (4.34-41.91) |
| Fanconi syndrome acquired | Renal and urinary disorders | 5 | 13.23 (5.49-31.84) |
| Hyperammonaemic encephalopathy | Nervous system disorders | 5 | 13.13 (5.45-31.62) |
| Menopausal symptoms | Reproductive system and breast disorders | 5 | 13.1 (5.44-31.54) |
| Ejection fraction decreased | Investigations | 36 | 13.01 (9.38-18.06) |
| Pharyngeal erythema | Respiratory, thoracic and mediastinal disorders | 5 | 12.94 (5.37-31.15) |
| Angiopathy | Vascular disorders | 11 | 12.93 (7.15-23.39) |
| Drug-induced liver injury | Hepatobiliary disorders | 75 | 12.91 (10.28-16.2) |
| Phlebitis | Vascular disorders | 10 | 12.84 (6.9-23.9) |
| Breast cancer metastatic | Neoplasms benign, malignant and unspecified (incl cysts and polyps) | 20 | 12.84 (8.27-19.93) |
| Skin hypopigmentation | Skin and subcutaneous tissue disorders | 5 | 12.68 (5.26-30.52) |
| Myocardial injury | Cardiac disorders | 4 | 12.5 (4.68-33.37) |
| Appendicitis perforated | Infections and infestations | 5 | 12.45 (5.17-29.98) |
| Skin sensitisation | Skin and subcutaneous tissue disorders | 3 | 12.36 (3.98-38.42) |
| Vena cava thrombosis | Vascular disorders | 3 | 12.28 (3.95-38.16) |
| Cardiac failure chronic | Cardiac disorders | 11 | 12.28 (6.79-22.21) |
| Adenocarcinoma | Neoplasms benign, malignant and unspecified (incl cysts and polyps) | 5 | 12.18 (5.06-29.32) |
| Hyperpyrexia | General disorders and administration site conditions | 8 | 12.07 (6.03-24.18) |
| Neutrophil count decreased | Investigations | 86 | 12.09 (9.78-14.96) |
| Multi-organ disorder | General disorders and administration site conditions | 3 | 11.95 (3.84-37.14) |
| Mucosal inflammation | General disorders and administration site conditions | 54 | 11.87 (9.08-15.51) |
| Body temperature abnormal | Investigations | 5 | 11.65 (4.84-28.03) |
| Odynophagia | Gastrointestinal disorders | 11 | 11.55 (6.39-20.89) |
| Mucosal dryness | General disorders and administration site conditions | 4 | 11.34 (4.25-30.29) |
| Catheter site erythema | General disorders and administration site conditions | 5 | 11.26 (4.68-27.1) |
| Metastases to bone | Neoplasms benign, malignant and unspecified (incl cysts and polyps) | 33 | 11.24 (7.98-15.83) |
| Mucosal disorder | General disorders and administration site conditions | 4 | 11.06 (4.14-29.53) |
| Acute myeloid leukaemia | Neoplasms benign, malignant and unspecified (incl cysts and polyps) | 29 | 11.05 (7.67-15.92) |
| Tetany | Metabolism and nutrition disorders | 3 | 10.91 (3.51-33.92) |
| Peripheral sensory neuropathy | Nervous system disorders | 11 | 10.77 (5.96-19.48) |
| Pseudomonal sepsis | Infections and infestations | 3 | 10.74 (3.46-33.39) |
| Maternal exposure timing unspecified | Injury, poisoning and procedural complications | 5 | 10.73 (4.46-25.83) |
| Cardiac failure acute | Cardiac disorders | 13 | 10.65 (6.18-18.37) |
| Nail infection | Infections and infestations | 3 | 10.58 (3.4-32.87) |
| Febrile neutropenia | Blood and lymphatic system disorders | 122 | 10.58 (8.85-12.65) |
| Neoplasm recurrence | Neoplasms benign, malignant and unspecified (incl cysts and polyps) | 6 | 10.43 (4.68-23.26) |
| Liver abscess | Infections and infestations | 6 | 10.4 (4.66-23.18) |
| Metastases to meninges | Neoplasms benign, malignant and unspecified (incl cysts and polyps) | 4 | 10.16 (3.8-27.11) |
| Malignant pleural effusion | Neoplasms benign, malignant and unspecified (incl cysts and polyps) | 3 | 10.11 (3.25-31.42) |
| Livedo reticularis | Skin and subcutaneous tissue disorders | 3 | 9.94 (3.2-30.9) |
| Mitral valve incompetence | Cardiac disorders | 14 | 9.86 (5.83-16.67) |
| Injection site hypersensitivity | General disorders and administration site conditions | 5 | 9.68 (4.02-23.3) |
| Intervertebral discitis | Infections and infestations | 3 | 9.66 (3.11-30.03) |
| Periodontitis | Infections and infestations | 3 | 9.64 (3.1-29.96) |
| Tricuspid valve incompetence | Cardiac disorders | 9 | 9.64 (5.01-18.55) |
| Polyneuropathy | Nervous system disorders | 21 | 9.64 (6.28-14.81) |
| Radiotherapy | Surgical and medical procedures | 3 | 9.45 (3.04-29.36) |
| Skin toxicity | Skin and subcutaneous tissue disorders | 9 | 9.37 (4.87-18.02) |
| Portal vein thrombosis | Hepatobiliary disorders | 5 | 9.27 (3.85-22.3) |
| Cerebral venous thrombosis | Nervous system disorders | 3 | 9.06 (2.92-28.14) |
| Venous thrombosis | Vascular disorders | 6 | 9.06 (4.06-20.19) |
| Lichenoid keratosis | Skin and subcutaneous tissue disorders | 3 | 8.98 (2.89-27.88) |
| Jaundice neonatal | Pregnancy, puerperium and perinatal conditions | 3 | 8.96 (2.88-27.83) |
| Gastrointestinal necrosis | Gastrointestinal disorders | 4 | 8.89 (3.33-23.71) |
| Lymphadenopathy mediastinal | Blood and lymphatic system disorders | 3 | 8.87 (2.86-27.55) |
| Sinus headache | Nervous system disorders | 8 | 8.87 (4.43-17.76) |
| Metastases to lung | Neoplasms benign, malignant and unspecified (incl cysts and polyps) | 19 | 8.83 (5.62-13.85) |
| Thrombocytosis | Blood and lymphatic system disorders | 6 | 8.8 (3.95-19.61) |
| Myocardial necrosis marker increased | Investigations | 3 | 8.66 (2.79-26.89) |
| Infusion site extravasation | General disorders and administration site conditions | 12 | 8.61 (4.89-15.18) |
| Muscle contracture | Musculoskeletal and connective tissue disorders | 3 | 8.55 (2.75-26.55) |
| Multiple-drug resistance | General disorders and administration site conditions | 5 | 8.4 (3.49-20.22) |
| Gingivitis | Infections and infestations | 8 | 8.37 (4.18-16.75) |
| Leukopenia | Blood and lymphatic system disorders | 70 | 8.27 (6.54-10.47) |
| Normochromic normocytic anaemia | Blood and lymphatic system disorders | 3 | 8.22 (2.65-25.53) |
| Pleuritic pain | Respiratory, thoracic and mediastinal disorders | 3 | 8.17 (2.63-25.39) |
| Neoplasm progression | Neoplasms benign, malignant and unspecified (incl cysts and polyps) | 64 | 8.07 (6.31-10.32) |
| Skin necrosis | Skin and subcutaneous tissue disorders | 7 | 7.97 (3.79-16.74) |
| Palmar-plantar erythrodysaesthesia syndrome | Skin and subcutaneous tissue disorders | 35 | 7.81 (5.6-10.89) |
| Aphthous ulcer | Gastrointestinal disorders | 14 | 7.67 (4.54-12.97) |
| White blood cell count decreased | Investigations | 158 | 7.75 (6.63-9.07) |
| Sputum discoloured | Respiratory, thoracic and mediastinal disorders | 15 | 7.64 (4.6-12.68) |
| Cardiac failure | Cardiac disorders | 109 | 7.68 (6.36-9.27) |
| Hepatitis B reactivation | Infections and infestations | 6 | 7.58 (3.4-16.89) |
| Neutropenia | Blood and lymphatic system disorders | 189 | 7.67 (6.64-8.85) |
| Intercepted product administration error | Injury, poisoning and procedural complications | 5 | 7.43 (3.09-17.86) |
| Iron deficiency | Metabolism and nutrition disorders | 6 | 7.4 (3.32-16.5) |
| Gastrointestinal toxicity | Gastrointestinal disorders | 6 | 7.28 (3.27-16.23) |
| Electrolyte imbalance | Metabolism and nutrition disorders | 14 | 7.13 (4.22-12.05) |
| Cytopenia | Blood and lymphatic system disorders | 17 | 7.11 (4.42-11.45) |
| Dermatomyositis | Skin and subcutaneous tissue disorders | 3 | 7.09 (2.28-22.02) |
| Premature labour | Pregnancy, puerperium and perinatal conditions | 6 | 7.07 (3.17-15.74) |
| Kounis syndrome | Cardiac disorders | 3 | 7.02 (2.26-21.81) |
| Spinal cord compression | Nervous system disorders | 5 | 6.87 (2.85-16.51) |
| Aplasia | Congenital, familial and genetic disorders | 3 | 6.86 (2.21-21.29) |
| Renal tubular disorder | Renal and urinary disorders | 3 | 6.83 (2.2-21.21) |
| Fibrin D dimer increased | Investigations | 4 | 6.83 (2.56-18.21) |
| Cachexia | Metabolism and nutrition disorders | 6 | 6.8 (3.05-15.16) |
| Haematotoxicity | Blood and lymphatic system disorders | 11 | 6.79 (3.76-12.28) |
| Laryngospasm | Respiratory, thoracic and mediastinal disorders | 3 | 6.79 (2.19-21.07) |
| Erythema nodosum | Skin and subcutaneous tissue disorders | 4 | 6.76 (2.53-18.04) |
| Disease recurrence | General disorders and administration site conditions | 59 | 6.76 (5.23-8.73) |
| Oligohydramnios | Pregnancy, puerperium and perinatal conditions | 4 | 6.67 (2.5-17.8) |
| Granulocytopenia | Blood and lymphatic system disorders | 5 | 6.67 (2.77-16.04) |
| Oral pain | Gastrointestinal disorders | 29 | 6.67 (4.63-9.61) |
| Atypical pneumonia | Infections and infestations | 4 | 6.55 (2.45-17.46) |
| Appetite disorder | Metabolism and nutrition disorders | 8 | 6.54 (3.27-13.1) |
| Radiation pneumonitis | Injury, poisoning and procedural complications | 3 | 6.49 (2.09-20.17) |
| Abdominal adhesions | Gastrointestinal disorders | 4 | 6.48 (2.43-17.27) |
| Faecaloma | Gastrointestinal disorders | 6 | 6.42 (2.88-14.3) |
| Mixed liver injury | Hepatobiliary disorders | 3 | 6.31 (2.03-19.6) |
| Metastases to peritoneum | Neoplasms benign, malignant and unspecified (incl cysts and polyps) | 3 | 6.27 (2.02-19.46) |
| Bundle branch block left | Cardiac disorders | 4 | 6.26 (2.35-16.7) |
| Oliguria | Renal and urinary disorders | 6 | 6.16 (2.77-13.73) |
| Transaminases increased | Investigations | 26 | 6.17 (4.19-9.06) |
| Sudden death | General disorders and administration site conditions | 9 | 6.11 (3.17-11.75) |
| Retinopathy | Eye disorders | 4 | 6.1 (2.29-16.27) |
| Metastases to liver | Neoplasms benign, malignant and unspecified (incl cysts and polyps) | 20 | 6.08 (3.92-9.44) |
| Pulmonary function test decreased | Investigations | 6 | 6.02 (2.7-13.42) |
| Lymphopenia | Blood and lymphatic system disorders | 16 | 5.98 (3.66-9.76) |
| Paranasal sinus discomfort | Respiratory, thoracic and mediastinal disorders | 5 | 5.89 (2.45-14.17) |
| Herpes simplex | Infections and infestations | 5 | 5.86 (2.44-14.1) |
| Foetal growth restriction | Pregnancy, puerperium and perinatal conditions | 7 | 5.77 (2.75-12.11) |
| Blood pressure diastolic increased | Investigations | 5 | 5.64 (2.35-13.57) |
| Vein disorder | Vascular disorders | 5 | 5.58 (2.32-13.42) |
| Nail disorder | Skin and subcutaneous tissue disorders | 8 | 5.56 (2.78-11.12) |
| Neutrophilia | Blood and lymphatic system disorders | 6 | 5.49 (2.46-12.22) |
| Interstitial lung disease | Respiratory, thoracic and mediastinal disorders | 46 | 5.49 (4.11-7.34) |
| Cystitis haemorrhagic | Renal and urinary disorders | 4 | 5.46 (2.05-14.57) |
| Premature baby | Pregnancy, puerperium and perinatal conditions | 32 | 5.42 (3.83-7.68) |
| Gamma-glutamyltransferase increased | Investigations | 17 | 5.34 (3.32-8.59) |
| Foetal death | Pregnancy, puerperium and perinatal conditions | 6 | 5.29 (2.37-11.78) |
| Hepatic failure | Hepatobiliary disorders | 25 | 5.22 (3.52-7.73) |
| Pneumocystis jirovecii pneumonia | Infections and infestations | 11 | 5.12 (2.83-9.26) |
| Pancreatitis acute | Gastrointestinal disorders | 18 | 5.11 (3.22-8.12) |
| Tumour marker increased | Investigations | 5 | 5.08 (2.11-12.21) |
| Energy increased | General disorders and administration site conditions | 6 | 5.04 (2.26-11.22) |
| Troponin increased | Investigations | 6 | 5.03 (2.26-11.21) |
| Incontinence | Renal and urinary disorders | 9 | 4.89 (2.54-9.4) |
| Gastroenteritis | Infections and infestations | 12 | 4.88 (2.77-8.59) |
| Septic shock | Infections and infestations | 35 | 4.7 (3.37-6.55) |
| Hepatocellular injury | Hepatobiliary disorders | 16 | 4.6 (2.81-7.51) |
| Neurotoxicity | Nervous system disorders | 15 | 4.56 (2.75-7.58) |
| Aspartate aminotransferase increased | Investigations | 33 | 4.56 (3.24-6.42) |
| Alanine aminotransferase increased | Investigations | 39 | 4.42 (3.23-6.06) |
| Deafness | Ear and labyrinth disorders | 21 | 4.37 (2.84-6.7) |
| Disease progression | General disorders and administration site conditions | 87 | 4.21 (3.41-5.2) |
| Pancytopenia | Blood and lymphatic system disorders | 38 | 4.19 (3.04-5.76) |
| Oral candidiasis | Infections and infestations | 9 | 4.15 (2.16-7.98) |
| Metastases to central nervous system | Neoplasms benign, malignant and unspecified (incl cysts and polyps) | 9 | 3.95 (2.05-7.6) |
| Neuropathy peripheral | Nervous system disorders | 71 | 3.89 (3.08-4.92) |
| Mouth ulceration | Gastrointestinal disorders | 14 | 3.87 (2.29-6.53) |
| Hypokalaemia | Metabolism and nutrition disorders | 30 | 3.84 (2.68-5.49) |
| Pleural effusion | Respiratory, thoracic and mediastinal disorders | 40 | 3.79 (2.78-5.17) |
| Cardiovascular disorder | Cardiac disorders | 10 | 3.78 (2.03-7.03) |
| Thrombocytopenia | Blood and lymphatic system disorders | 68 | 3.58 (2.82-4.54) |
| Pyrexia | General disorders and administration site conditions | 217 | 3.6 (3.15-4.12) |
| Full blood count decreased | Investigations | 15 | 3.49 (2.1-5.79) |
| Hyponatraemia | Metabolism and nutrition disorders | 33 | 3.35 (2.38-4.72) |
| Asthenia | General disorders and administration site conditions | 222 | 3.29 (2.88-3.75) |
| Vomiting | Gastrointestinal disorders | 251 | 3.13 (2.76-3.54) |
| Hepatic enzyme increased | Investigations | 34 | 2.95 (2.11-4.13) |
| Anaemia | Blood and lymphatic system disorders | 98 | 2.92 (2.39-3.56) |
