# Supplementary material for: Epirubicin and gait apraxia: a real-world data analysis of the FDA Adverse Event Reporting System database
Source: Front Pharmacol. 2023 Sep 14;14:1249845. doi: 10.3389/fphar.2023.1249845 (PMC10536159; doi:10.3389/fphar.2023.1249845)
Supplement: Supplementary file 3 [file Table5.docx]

Supplementary Table S5 The comprehensive detailed information of all AEs at the PTs level identified by EBGM algorithms.

| Preferred Terms | SOC | Case Number | EBGM (95% two side CI) |
| --- | --- | --- | --- |
| Vomiting | Gastrointestinal disorders | 251 | 3.08 (2.72-3.49) |
| Asthenia | General disorders and administration site conditions | 222 | 3.24 (2.84-3.7) |
| Pyrexia | General disorders and administration site conditions | 217 | 3.55 (3.1-4.06) |
| Neutropenia | Blood and lymphatic system disorders | 189 | 7.54 (6.53-8.71) |
| Hepatic function abnormal | Hepatobiliary disorders | 161 | 26.94 (23.04-31.49) |
| White blood cell count decreased | Investigations | 158 | 7.64 (6.53-8.94) |
| Febrile neutropenia | Blood and lymphatic system disorders | 122 | 10.45 (8.74-12.49) |
| Myelosuppression | Blood and lymphatic system disorders | 121 | 31.78 (26.54-38.05) |
| Cardiac failure | Cardiac disorders | 109 | 7.6 (6.29-9.18) |
| Bone marrow failure | Blood and lymphatic system disorders | 105 | 24.55 (20.24-29.77) |
| Anaemia | Blood and lymphatic system disorders | 98 | 2.9 (2.38-3.54) |
| Disease progression | General disorders and administration site conditions | 87 | 4.19 (3.39-5.17) |
| Neutrophil count decreased | Investigations | 86 | 11.96 (9.67-14.8) |
| Drug-induced liver injury | Hepatobiliary disorders | 75 | 12.78 (10.18-16.04) |
| Neuropathy peripheral | Nervous system disorders | 71 | 3.87 (3.07-4.89) |
| Leukopenia | Blood and lymphatic system disorders | 70 | 8.21 (6.49-10.39) |
| Thrombocytopenia | Blood and lymphatic system disorders | 68 | 3.56 (2.8-4.52) |
| Neoplasm progression | Neoplasms benign, malignant and unspecified (incl cysts and polyps) | 64 | 8.01 (6.27-10.25) |
| Agranulocytosis | Blood and lymphatic system disorders | 63 | 20.87 (16.28-26.76) |
| Liver injury | Hepatobiliary disorders | 61 | 16.48 (12.8-21.21) |
| Disease recurrence | General disorders and administration site conditions | 59 | 6.72 (5.2-8.68) |
| Neutropenic sepsis | Infections and infestations | 55 | 42.23 (32.34-55.14) |
| Mucosal inflammation | General disorders and administration site conditions | 54 | 11.77 (9.01-15.39) |
| Cardiotoxicity | Cardiac disorders | 53 | 31.49 (24.01-41.3) |
| Cardiac dysfunction | Cardiac disorders | 49 | 68.67 (51.7-91.21) |
| Interstitial lung disease | Respiratory, thoracic and mediastinal disorders | 46 | 5.46 (4.09-7.3) |
| Dilated cardiomyopathy | Cardiac disorders | 43 | 50.79 (37.55-68.7) |
| Second primary malignancy | Neoplasms benign, malignant and unspecified (incl cysts and polyps) | 43 | 21.9 (16.21-29.57) |
| Pleural effusion | Respiratory, thoracic and mediastinal disorders | 40 | 3.77 (2.77-5.15) |
| Alanine aminotransferase increased | Investigations | 39 | 4.41 (3.22-6.03) |
| Pancytopenia | Blood and lymphatic system disorders | 38 | 4.17 (3.03-5.74) |
| Ejection fraction decreased | Investigations | 36 | 12.93 (9.31-17.94) |
| Palmar-plantar erythrodysaesthesia syndrome | Skin and subcutaneous tissue disorders | 35 | 7.77 (5.58-10.84) |
| Septic shock | Infections and infestations | 35 | 4.69 (3.36-6.53) |
| Hepatic enzyme increased | Investigations | 34 | 2.94 (2.1-4.12) |
| Aspartate aminotransferase increased | Investigations | 33 | 4.54 (3.23-6.4) |
| Metastases to bone | Neoplasms benign, malignant and unspecified (incl cysts and polyps) | 33 | 11.17 (7.93-15.73) |
| Hyponatraemia | Metabolism and nutrition disorders | 33 | 3.34 (2.37-4.7) |
| Premature baby | Pregnancy, puerperium and perinatal conditions | 32 | 5.4 (3.82-7.65) |
| Cardiomyopathy | Cardiac disorders | 32 | 13.78 (9.73-19.51) |
| Hypokalaemia | Metabolism and nutrition disorders | 30 | 3.82 (2.67-5.47) |
| Acute myeloid leukaemia | Neoplasms benign, malignant and unspecified (incl cysts and polyps) | 29 | 10.99 (7.63-15.83) |
| Oral pain | Gastrointestinal disorders | 29 | 6.65 (4.61-9.57) |
| Transaminases increased | Investigations | 26 | 6.14 (4.18-9.03) |
| Granulocyte count decreased | Investigations | 26 | 138.27 (93.24-205.06) |
| Hepatic failure | Hepatobiliary disorders | 25 | 5.2 (3.51-7.7) |
| Breast cancer recurrent | Neoplasms benign, malignant and unspecified (incl cysts and polyps) | 22 | 41.99 (27.56-63.99) |
| Hypertransaminasaemia | Hepatobiliary disorders | 21 | 19.67 (12.8-30.23) |
| Polyneuropathy | Nervous system disorders | 21 | 9.6 (6.25-14.74) |
| Deafness | Ear and labyrinth disorders | 21 | 4.35 (2.84-6.68) |
| Left ventricular dysfunction | Cardiac disorders | 20 | 17.07 (11-26.51) |
| Breast cancer metastatic | Neoplasms benign, malignant and unspecified (incl cysts and polyps) | 20 | 12.77 (8.23-19.82) |
| Febrile bone marrow aplasia | Blood and lymphatic system disorders | 20 | 25.57 (16.46-39.72) |
| Metastases to liver | Neoplasms benign, malignant and unspecified (incl cysts and polyps) | 20 | 6.07 (3.91-9.41) |
| Metastases to lung | Neoplasms benign, malignant and unspecified (incl cysts and polyps) | 19 | 8.79 (5.6-13.8) |
| Hepatitis B | Infections and infestations | 19 | 19.04 (12.12-29.91) |
| Administration site extravasation | General disorders and administration site conditions | 19 | 86.02 (54.49-135.81) |
| Pancreatitis acute | Gastrointestinal disorders | 18 | 5.1 (3.21-8.09) |
| Gamma-glutamyltransferase increased | Investigations | 17 | 5.32 (3.31-8.57) |
| Metastases to lymph nodes | Neoplasms benign, malignant and unspecified (incl cysts and polyps) | 17 | 14.35 (8.91-23.11) |
| Metastasis | Neoplasms benign, malignant and unspecified (incl cysts and polyps) | 17 | 13.63 (8.46-21.96) |
| Cytopenia | Blood and lymphatic system disorders | 17 | 7.09 (4.4-11.42) |
| Hepatocellular injury | Hepatobiliary disorders | 16 | 4.59 (2.81-7.49) |
| Lymphopenia | Blood and lymphatic system disorders | 16 | 5.96 (3.65-9.73) |
| Extravasation | General disorders and administration site conditions | 16 | 22.27 (13.61-36.42) |
| Full blood count decreased | Investigations | 15 | 3.49 (2.1-5.78) |
| Neurotoxicity | Nervous system disorders | 15 | 4.55 (2.74-7.56) |
| Sputum discoloured | Respiratory, thoracic and mediastinal disorders | 15 | 7.61 (4.59-12.64) |
| Electrolyte imbalance | Metabolism and nutrition disorders | 14 | 7.11 (4.21-12.01) |
| Mouth ulceration | Gastrointestinal disorders | 14 | 3.86 (2.28-6.52) |
| Aphthous ulcer | Gastrointestinal disorders | 14 | 7.65 (4.52-12.93) |
| Mitral valve incompetence | Cardiac disorders | 14 | 9.82 (5.81-16.6) |
| Hepatic lesion | Hepatobiliary disorders | 13 | 16.99 (9.85-29.31) |
| Cardiac failure acute | Cardiac disorders | 13 | 10.61 (6.15-18.29) |
| Acute pulmonary oedema | Respiratory, thoracic and mediastinal disorders | 13 | 15.09 (8.75-26.04) |
| Infusion site extravasation | General disorders and administration site conditions | 12 | 8.58 (4.87-15.13) |
| Gastroenteritis | Infections and infestations | 12 | 4.87 (2.76-8.57) |
| Odynophagia | Gastrointestinal disorders | 11 | 11.5 (6.36-20.8) |
| Pneumocystis jirovecii pneumonia | Infections and infestations | 11 | 5.11 (2.83-9.24) |
| Angiopathy | Vascular disorders | 11 | 12.87 (7.12-23.28) |
| Peripheral sensory neuropathy | Nervous system disorders | 11 | 10.73 (5.93-19.4) |
| Ventricular hypokinesia | Cardiac disorders | 11 | 21.89 (12.09-39.63) |
| Haematotoxicity | Blood and lymphatic system disorders | 11 | 6.78 (3.75-12.25) |
| Cardiac failure chronic | Cardiac disorders | 11 | 12.22 (6.76-22.1) |
| Placental disorder | Pregnancy, puerperium and perinatal conditions | 10 | 76.11 (40.61-142.64) |
| Cardiovascular disorder | Cardiac disorders | 10 | 3.77 (2.03-7.02) |
| Subacute cutaneous lupus erythematosus | Skin and subcutaneous tissue disorders | 10 | 24.66 (13.23-45.96) |
| Phlebitis | Vascular disorders | 10 | 12.78 (6.86-23.79) |
| Arterial thrombosis | Vascular disorders | 10 | 32.5 (17.42-60.63) |
| Oral candidiasis | Infections and infestations | 9 | 4.14 (2.15-7.96) |
| Skin toxicity | Skin and subcutaneous tissue disorders | 9 | 9.33 (4.85-17.96) |
| Incontinence | Renal and urinary disorders | 9 | 4.88 (2.54-9.39) |
| Sudden death | General disorders and administration site conditions | 9 | 6.09 (3.17-11.72) |
| Tricuspid valve incompetence | Cardiac disorders | 9 | 9.6 (4.99-18.48) |
| Metastases to central nervous system | Neoplasms benign, malignant and unspecified (incl cysts and polyps) | 9 | 3.95 (2.05-7.59) |
| Xerophthalmia | Eye disorders | 8 | 83.16 (41.17-167.99) |
| Hepatic artery stenosis | Hepatobiliary disorders | 8 | 468.01 (219.99-995.63) |
| Subclavian vein thrombosis | Vascular disorders | 8 | 44.45 (22.11-89.37) |
| Appetite disorder | Metabolism and nutrition disorders | 8 | 6.53 (3.26-13.06) |
| Hyperpyrexia | General disorders and administration site conditions | 8 | 12.02 (6-24.07) |
| Acute lymphocytic leukaemia | Neoplasms benign, malignant and unspecified (incl cysts and polyps) | 8 | 14.53 (7.25-29.11) |
| Sinus headache | Nervous system disorders | 8 | 8.84 (4.42-17.7) |
| Nail disorder | Skin and subcutaneous tissue disorders | 8 | 5.55 (2.77-11.1) |
| Tongue coated | Gastrointestinal disorders | 8 | 31.74 (15.81-63.72) |
| Acute leukaemia | Neoplasms benign, malignant and unspecified (incl cysts and polyps) | 8 | 33.48 (16.67-67.22) |
| Menopause | Social circumstances | 8 | 18.93 (9.44-37.94) |
| Bladder irritation | Renal and urinary disorders | 8 | 62.81 (31.17-126.56) |
| Gingivitis | Infections and infestations | 8 | 8.34 (4.17-16.7) |
| Foetal growth restriction | Pregnancy, puerperium and perinatal conditions | 7 | 5.75 (2.74-12.08) |
| Neutropenic colitis | Gastrointestinal disorders | 7 | 18.55 (8.82-39.01) |
| Diastolic dysfunction | Cardiac disorders | 7 | 14.74 (7.01-30.98) |
| Psychotic behaviour | Psychiatric disorders | 7 | 42.45 (20.12-89.54) |
| Skin necrosis | Skin and subcutaneous tissue disorders | 7 | 7.95 (3.78-16.69) |
| Right atrial dilatation | Cardiac disorders | 7 | 97.59 (45.94-207.32) |
| Catheter site pain | General disorders and administration site conditions | 7 | 13.87 (6.6-29.15) |
| Oliguria | Renal and urinary disorders | 6 | 6.15 (2.76-13.7) |
| Aortic dilatation | Vascular disorders | 6 | 34.9 (15.6-78.06) |
| Neutrophilia | Blood and lymphatic system disorders | 6 | 5.48 (2.46-12.2) |
| Coronary artery thrombosis | Cardiac disorders | 6 | 24.62 (11.02-55) |
| Carbohydrate antigen 15-3 increased | Investigations | 6 | 43.03 (19.22-96.37) |
| Foetal death | Pregnancy, puerperium and perinatal conditions | 6 | 5.28 (2.37-11.76) |
| Hepatitis B reactivation | Infections and infestations | 6 | 7.56 (3.39-16.85) |
| Cachexia | Metabolism and nutrition disorders | 6 | 6.79 (3.05-15.12) |
| Energy increased | General disorders and administration site conditions | 6 | 5.03 (2.26-11.2) |
| Premature labour | Pregnancy, puerperium and perinatal conditions | 6 | 7.05 (3.16-15.71) |
| Troponin I increased | Investigations | 6 | 22.49 (10.07-50.22) |
| Dysentery | Infections and infestations | 6 | 14.84 (6.65-33.11) |
| Faecaloma | Gastrointestinal disorders | 6 | 6.4 (2.87-14.27) |
| Iron deficiency | Metabolism and nutrition disorders | 6 | 7.38 (3.31-16.46) |
| Gastrointestinal toxicity | Gastrointestinal disorders | 6 | 7.26 (3.26-16.18) |
| Neoplasm recurrence | Neoplasms benign, malignant and unspecified (incl cysts and polyps) | 6 | 10.4 (4.66-23.18) |
| Thrombocytosis | Blood and lymphatic system disorders | 6 | 8.77 (3.93-19.55) |
| Troponin increased | Investigations | 6 | 5.02 (2.25-11.19) |
| N-terminal prohormone brain natriuretic peptide increased | Investigations | 6 | 21.29 (9.53-47.53) |
| Liver abscess | Infections and infestations | 6 | 10.36 (4.65-23.1) |
| Venous thrombosis | Vascular disorders | 6 | 9.03 (4.05-20.12) |
| Pulmonary function test decreased | Investigations | 6 | 6.01 (2.7-13.39) |
| Catheter site erythema | General disorders and administration site conditions | 5 | 11.22 (4.66-27) |
| Blood pressure diastolic increased | Investigations | 5 | 5.63 (2.34-13.55) |
| Intercepted product administration error | Injury, poisoning and procedural complications | 5 | 7.41 (3.08-17.82) |
| Catheter site related reaction | General disorders and administration site conditions | 5 | 49.4 (20.41-119.58) |
| Injection site hypersensitivity | General disorders and administration site conditions | 5 | 9.65 (4.01-23.22) |
| Paranasal sinus discomfort | Respiratory, thoracic and mediastinal disorders | 5 | 5.88 (2.44-14.14) |
| Troponin T increased | Investigations | 5 | 23.57 (9.77-56.83) |
| Herpes simplex | Infections and infestations | 5 | 5.85 (2.43-14.08) |
| Skin hypopigmentation | Skin and subcutaneous tissue disorders | 5 | 12.62 (5.24-30.38) |
| Mental fatigue | Psychiatric disorders | 5 | 16.36 (6.79-39.4) |
| Hyperammonaemic encephalopathy | Nervous system disorders | 5 | 13.07 (5.43-31.48) |
| Body temperature abnormal | Investigations | 5 | 11.6 (4.82-27.92) |
| Adenocarcinoma | Neoplasms benign, malignant and unspecified (incl cysts and polyps) | 5 | 12.13 (5.04-29.2) |
| Portal vein thrombosis | Hepatobiliary disorders | 5 | 9.24 (3.84-22.23) |
| Vein disorder | Vascular disorders | 5 | 5.57 (2.32-13.4) |
| Granulocytopenia | Blood and lymphatic system disorders | 5 | 6.65 (2.77-16) |
| Spinal cord compression | Nervous system disorders | 5 | 6.85 (2.85-16.48) |
| Tumour marker increased | Investigations | 5 | 5.07 (2.11-12.19) |
| Maternal exposure timing unspecified | Injury, poisoning and procedural complications | 5 | 10.69 (4.44-25.74) |
| Pharyngeal erythema | Respiratory, thoracic and mediastinal disorders | 5 | 12.88 (5.35-31.02) |
| Jugular vein thrombosis | Vascular disorders | 5 | 18.26 (7.58-44) |
| Appendicitis perforated | Infections and infestations | 5 | 12.4 (5.15-29.85) |
| Myocardial fibrosis | Cardiac disorders | 5 | 35.35 (14.63-85.39) |
| Aortic thrombosis | Vascular disorders | 5 | 27.68 (11.47-66.78) |
| Multiple-drug resistance | General disorders and administration site conditions | 5 | 8.38 (3.48-20.16) |
| Ventricular dysfunction | Cardiac disorders | 5 | 20.63 (8.56-49.73) |
| Fanconi syndrome acquired | Renal and urinary disorders | 5 | 13.17 (5.47-31.7) |
| Pancreatitis necrotising | Gastrointestinal disorders | 5 | 14.83 (6.16-35.71) |
| Menopausal symptoms | Reproductive system and breast disorders | 5 | 13.04 (5.42-31.4) |
| Allodynia | Nervous system disorders | 5 | 37.86 (15.67-91.5) |
| Dilatation ventricular | Cardiac disorders | 5 | 25.9 (10.74-62.47) |
| Abdominal adhesions | Gastrointestinal disorders | 4 | 6.46 (2.42-17.24) |
| Cystitis haemorrhagic | Renal and urinary disorders | 4 | 5.45 (2.04-14.55) |
| Post embolisation syndrome | Injury, poisoning and procedural complications | 4 | 195.64 (70.97-539.29) |
| Cardiac death | General disorders and administration site conditions | 4 | 20.76 (7.76-55.5) |
| Respiratory alkalosis | Respiratory, thoracic and mediastinal disorders | 4 | 15.54 (5.82-41.52) |
| Metastases to the mediastinum | Neoplasms benign, malignant and unspecified (incl cysts and polyps) | 4 | 48.71 (18.13-130.87) |
| Soft tissue sarcoma | Neoplasms benign, malignant and unspecified (incl cysts and polyps) | 4 | 41.73 (15.55-111.97) |
| Oligohydramnios | Pregnancy, puerperium and perinatal conditions | 4 | 6.66 (2.49-17.76) |
| Metastases to meninges | Neoplasms benign, malignant and unspecified (incl cysts and polyps) | 4 | 10.12 (3.79-27.02) |
| Hydrothorax | Respiratory, thoracic and mediastinal disorders | 4 | 22.35 (8.36-59.78) |
| Mucosal disorder | General disorders and administration site conditions | 4 | 11.02 (4.13-29.42) |
| Bile duct stenosis | Hepatobiliary disorders | 4 | 20.09 (7.51-53.72) |
| Aortitis | Vascular disorders | 4 | 30.37 (11.34-81.33) |
| Administration site oedema | General disorders and administration site conditions | 4 | 284.15 (101.4-796.27) |
| Acute cutaneous lupus erythematosus | Skin and subcutaneous tissue disorders | 4 | 110.5 (40.7-300.02) |
| Soft tissue necrosis | Musculoskeletal and connective tissue disorders | 4 | 36.5 (13.61-97.85) |
| Neutrophil percentage decreased | Investigations | 4 | 70.2 (26.04-189.29) |
| Fibrin D dimer increased | Investigations | 4 | 6.81 (2.55-18.17) |
| Myocardial injury | Cardiac disorders | 4 | 12.44 (4.66-33.23) |
| Miller Fisher syndrome | Nervous system disorders | 4 | 102 (37.62-276.54) |
| Pseudocirrhosis | Hepatobiliary disorders | 4 | 53.52 (19.9-143.89) |
| Gastrointestinal necrosis | Gastrointestinal disorders | 4 | 8.86 (3.32-23.65) |
| Bundle branch block left | Cardiac disorders | 4 | 6.25 (2.34-16.67) |
| Abdominal sepsis | Infections and infestations | 4 | 27.31 (10.2-73.11) |
| Erythema nodosum | Skin and subcutaneous tissue disorders | 4 | 6.75 (2.53-18) |
| Atypical pneumonia | Infections and infestations | 4 | 6.53 (2.45-17.43) |
| Retinopathy | Eye disorders | 4 | 6.09 (2.28-16.24) |
| Mucosal dryness | General disorders and administration site conditions | 4 | 11.3 (4.23-30.17) |
| Malignant pleural effusion | Neoplasms benign, malignant and unspecified (incl cysts and polyps) | 3 | 10.08 (3.24-31.32) |
| Normochromic normocytic anaemia | Blood and lymphatic system disorders | 3 | 8.2 (2.64-25.46) |
| Kidney enlargement | Renal and urinary disorders | 3 | 17.41 (5.6-54.18) |
| Infusion site discolouration | General disorders and administration site conditions | 3 | 18.42 (5.92-57.31) |
| Livedo reticularis | Skin and subcutaneous tissue disorders | 3 | 9.91 (3.19-30.8) |
| Muscle contracture | Musculoskeletal and connective tissue disorders | 3 | 8.52 (2.74-26.48) |
| Skin sensitisation | Skin and subcutaneous tissue disorders | 3 | 12.31 (3.96-38.27) |
| Lichenoid keratosis | Skin and subcutaneous tissue disorders | 3 | 8.95 (2.88-27.8) |
| Periodontitis | Infections and infestations | 3 | 9.61 (3.09-29.87) |
| Hyperlipasaemia | Metabolism and nutrition disorders | 3 | 28.41 (9.11-88.6) |
| Atypical haemolytic uraemic syndrome | Blood and lymphatic system disorders | 3 | 18.45 (5.93-57.43) |
| Lymphangiosis carcinomatosa | Neoplasms benign, malignant and unspecified (incl cysts and polyps) | 3 | 15.46 (4.97-48.08) |
| Radiation pneumonitis | Injury, poisoning and procedural complications | 3 | 6.48 (2.09-20.12) |
| Tetany | Metabolism and nutrition disorders | 3 | 10.88 (3.5-33.8) |
| Adenocarcinoma gastric | Neoplasms benign, malignant and unspecified (incl cysts and polyps) | 3 | 14.12 (4.54-43.9) |
| Lymphadenopathy mediastinal | Blood and lymphatic system disorders | 3 | 8.84 (2.85-27.47) |
| Refractory cancer | Neoplasms benign, malignant and unspecified (incl cysts and polyps) | 3 | 62.59 (19.94-196.46) |
| Nail infection | Infections and infestations | 3 | 10.54 (3.39-32.76) |
| Biliary dilatation | Hepatobiliary disorders | 3 | 13.56 (4.36-42.16) |
| Vena cava thrombosis | Vascular disorders | 3 | 12.23 (3.93-38.01) |
| Laryngospasm | Respiratory, thoracic and mediastinal disorders | 3 | 6.77 (2.18-21.02) |
| Iatrogenic injury | Injury, poisoning and procedural complications | 3 | 26.33 (8.45-82.05) |
| Kounis syndrome | Cardiac disorders | 3 | 7.01 (2.26-21.76) |
| Biliary tract disorder | Hepatobiliary disorders | 3 | 15.09 (4.85-46.94) |
| Cerebral venous thrombosis | Nervous system disorders | 3 | 9.03 (2.91-28.06) |
| Aplasia | Congenital, familial and genetic disorders | 3 | 6.84 (2.2-21.25) |
| Intervertebral discitis | Infections and infestations | 3 | 9.63 (3.1-29.93) |
| Right ventricular dysfunction | Cardiac disorders | 3 | 14.53 (4.67-45.18) |
| Metastases to thorax | Neoplasms benign, malignant and unspecified (incl cysts and polyps) | 3 | 59.28 (18.9-185.93) |
| Dermatomyositis | Skin and subcutaneous tissue disorders | 3 | 7.08 (2.28-21.97) |
| Myocardial oedema | Cardiac disorders | 3 | 41.83 (13.38-130.75) |
| Acute promyelocytic leukaemia | Neoplasms benign, malignant and unspecified (incl cysts and polyps) | 3 | 19.89 (6.39-61.92) |
| Biliary fistula | Hepatobiliary disorders | 3 | 119.34 (37.6-378.82) |
| Endocardial fibrosis | Cardiac disorders | 3 | 426.22 (125.53-1447.17) |
| Gait apraxia | Nervous system disorders | 3 | 426.22 (125.53-1447.17) |
| Hyperamylasaemia | Metabolism and nutrition disorders | 3 | 31.63 (10.14-98.67) |
| Pleuritic pain | Respiratory, thoracic and mediastinal disorders | 3 | 8.15 (2.62-25.32) |
| Jaundice neonatal | Pregnancy, puerperium and perinatal conditions | 3 | 8.93 (2.88-27.75) |
| Intestinal atresia | Congenital, familial and genetic disorders | 3 | 127.87 (40.21-406.58) |
| Anal ulcer | Gastrointestinal disorders | 3 | 20.44 (6.56-63.62) |
| Myocardial necrosis marker increased | Investigations | 3 | 8.63 (2.78-26.81) |
| Cutaneous symptom | Skin and subcutaneous tissue disorders | 3 | 19.8 (6.36-61.64) |
| Menopausal disorder | Reproductive system and breast disorders | 3 | 142.07 (44.55-453.07) |
| Metastases to peritoneum | Neoplasms benign, malignant and unspecified (incl cysts and polyps) | 3 | 6.25 (2.01-19.42) |
| Multi-organ disorder | General disorders and administration site conditions | 3 | 11.9 (3.83-36.99) |
| Hepatic artery occlusion | Hepatobiliary disorders | 3 | 308.64 (93.4-1019.87) |
| Cardiac perfusion defect | Cardiac disorders | 3 | 331.5 (99.81-1101.06) |
| Hypercreatininaemia | Metabolism and nutrition disorders | 3 | 34.83 (11.16-108.72) |
| Ocular toxicity | Eye disorders | 3 | 18.88 (6.07-58.77) |
| Anastomotic leak | Injury, poisoning and procedural complications | 3 | 26.64 (8.55-83.03) |
| Pseudomonal sepsis | Infections and infestations | 3 | 10.71 (3.45-33.27) |
| Renal tubular disorder | Renal and urinary disorders | 3 | 6.82 (2.2-21.17) |
| Radiotherapy | Surgical and medical procedures | 3 | 9.42 (3.03-29.27) |
| Neutropenic infection | Infections and infestations | 3 | 22.89 (7.35-71.3) |
| Axillary mass | Musculoskeletal and connective tissue disorders | 3 | 15.65 (5.03-48.67) |
| Mixed liver injury | Hepatobiliary disorders | 3 | 6.3 (2.03-19.56) |
| Amnestic disorder | Nervous system disorders | 3 | 33.03 (10.58-103.07) |
| Merycism | Psychiatric disorders | 3 | 52.96 (16.91-165.92) |
| Dislocation of vertebra | Injury, poisoning and procedural complications | 3 | 13.42 (4.32-41.72) |
| Metastases to chest wall | Neoplasms benign, malignant and unspecified (incl cysts and polyps) | 2 | 29.25 (7.26-117.78) |
| Infusion site vesicles | General disorders and administration site conditions | 2 | 18.3 (4.56-73.51) |
| Leukocyturia | Renal and urinary disorders | 2 | 14.59 (3.64-58.54) |
| Neonatal infection | Infections and infestations | 2 | 31.24 (7.76-125.85) |
| Lividity | Skin and subcutaneous tissue disorders | 2 | 35.95 (8.91-144.97) |
| Transaminases abnormal | Investigations | 2 | 17.65 (4.4-70.89) |
| Nail dystrophy | Skin and subcutaneous tissue disorders | 2 | 21.31 (5.3-85.65) |
| Diffuse alveolar damage | Respiratory, thoracic and mediastinal disorders | 2 | 10.52 (2.63-42.19) |
| Left ventricular dilatation | Cardiac disorders | 2 | 15.5 (3.86-62.2) |
| Electrocardiogram T wave abnormal | Investigations | 2 | 10.29 (2.57-41.24) |
| Cerebral haemorrhage neonatal | Nervous system disorders | 2 | 55.77 (13.76-225.96) |
| Hypercreatinaemia | Musculoskeletal and connective tissue disorders | 2 | 33.15 (8.23-133.6) |
| Alopecia totalis | Skin and subcutaneous tissue disorders | 2 | 30.76 (7.64-123.89) |
| Metastases to ovary | Neoplasms benign, malignant and unspecified (incl cysts and polyps) | 2 | 33.15 (8.23-133.6) |
| Scedosporium infection | Infections and infestations | 2 | 16.62 (4.14-66.73) |
| Breast calcifications | Reproductive system and breast disorders | 2 | 15.62 (3.89-62.7) |
| Breast necrosis | Reproductive system and breast disorders | 2 | 84.04 (20.6-342.86) |
| Vasogenic cerebral oedema | Nervous system disorders | 2 | 17.55 (4.37-70.47) |
| Catheter site vesicles | General disorders and administration site conditions | 2 | 40.87 (10.12-165.02) |
| Administration site pain | General disorders and administration site conditions | 2 | 8.99 (2.24-36.01) |
| Blood bilirubin abnormal | Investigations | 2 | 9.35 (2.33-37.48) |
| Neutrophil percentage increased | Investigations | 2 | 10.67 (2.66-42.79) |
| Infusion site scar | General disorders and administration site conditions | 2 | 23.22 (5.77-93.35) |
| Hypertelorism | Congenital, familial and genetic disorders | 2 | 22.78 (5.66-91.56) |
| Toxic cardiomyopathy | Cardiac disorders | 2 | 49.73 (12.29-201.19) |
| Pleocytosis | Nervous system disorders | 2 | 18.47 (4.6-74.2) |
| Hepatic adenoma | Neoplasms benign, malignant and unspecified (incl cysts and polyps) | 2 | 25.61 (6.37-103.03) |
| Catheter site swelling | General disorders and administration site conditions | 2 | 10.62 (2.65-42.56) |
| Mucosal ulceration | General disorders and administration site conditions | 2 | 12.23 (3.05-49.04) |
| Acute hepatitis B | Infections and infestations | 2 | 33.52 (8.32-135.12) |
| Paraneoplastic syndrome | Neoplasms benign, malignant and unspecified (incl cysts and polyps) | 2 | 21.39 (5.32-85.96) |
| Diffuse large B-cell lymphoma stage II | Neoplasms benign, malignant and unspecified (incl cysts and polyps) | 2 | 161.27 (38.78-670.61) |
| Glomerular filtration rate increased | Investigations | 2 | 14.07 (3.51-56.46) |
| Infusion site joint movement impairment | General disorders and administration site conditions | 2 | 745.88 (150.53-3696) |
| Catheter site extravasation | General disorders and administration site conditions | 2 | 20.23 (5.03-81.27) |
| Necrotising colitis | Gastrointestinal disorders | 2 | 9.77 (2.44-39.14) |
| Haemangioma of bone | Neoplasms benign, malignant and unspecified (incl cysts and polyps) | 2 | 61.52 (15.16-249.6) |
| Bone marrow transplant rejection | Immune system disorders | 2 | 64.16 (15.8-260.5) |
| Strangury | Renal and urinary disorders | 2 | 60.89 (15.01-247.02) |
| Obliterative bronchiolitis | Respiratory, thoracic and mediastinal disorders | 2 | 8.64 (2.16-34.6) |
| Infusion site necrosis | General disorders and administration site conditions | 2 | 81.74 (20.05-333.28) |
| Systolic dysfunction | Cardiac disorders | 2 | 8.83 (2.2-35.37) |
| Leukoplakia oral | Gastrointestinal disorders | 2 | 23.77 (5.91-95.6) |
| Axillary vein thrombosis | Vascular disorders | 2 | 39.52 (9.79-159.5) |
| Chalazion | Eye disorders | 2 | 17.76 (4.42-71.31) |
| Peripheral nerve injury | Injury, poisoning and procedural complications | 2 | 12.46 (3.11-49.96) |
| Heart transplant rejection | Immune system disorders | 2 | 11.17 (2.79-44.8) |
| Arterial stenosis | Vascular disorders | 2 | 14.96 (3.73-60.01) |
| Chondrodynia | Musculoskeletal and connective tissue disorders | 2 | 663.01 (137.71-3191.96) |
| Monocyte count decreased | Investigations | 2 | 8.69 (2.17-34.8) |
| Necrotising enterocolitis neonatal | Gastrointestinal disorders | 2 | 35.73 (8.86-144.09) |
| Catheter site thrombosis | General disorders and administration site conditions | 2 | 38.75 (9.6-156.37) |
| Hypoproteinaemia | Metabolism and nutrition disorders | 2 | 8.46 (2.11-33.91) |
| IIIrd nerve paralysis | Nervous system disorders | 2 | 16.44 (4.09-65.99) |
| Epidermal necrosis | Skin and subcutaneous tissue disorders | 2 | 13.03 (3.25-52.26) |
| Mucosal toxicity | General disorders and administration site conditions | 2 | 27.37 (6.8-110.16) |
| Inner ear disorder | Ear and labyrinth disorders | 2 | 11.37 (2.83-45.57) |
| Pulmonary hilum mass | Respiratory, thoracic and mediastinal disorders | 2 | 73.67 (18.1-299.78) |
| Haemorrhagic necrotic pancreatitis | Gastrointestinal disorders | 2 | 94.72 (23.16-387.4) |
| Xerosis | General disorders and administration site conditions | 2 | 11.45 (2.86-45.92) |
| Breast angiosarcoma metastatic | Neoplasms benign, malignant and unspecified (incl cysts and polyps) | 2 | 994.51 (182.13-5430.36) |
| Nail pigmentation | Skin and subcutaneous tissue disorders | 2 | 39.78 (9.86-160.57) |
| Protrusion tongue | Gastrointestinal disorders | 2 | 21.54 (5.36-86.58) |
| Hilar lymphadenopathy | Blood and lymphatic system disorders | 2 | 14.99 (3.74-60.17) |
| Lymphocyte percentage decreased | Investigations | 2 | 8.97 (2.24-35.96) |
| Vascular pain | Vascular disorders | 2 | 9.35 (2.33-37.48) |
| Tumour rupture | Neoplasms benign, malignant and unspecified (incl cysts and polyps) | 2 | 18.25 (4.54-73.29) |
| Radiation neuropathy | Injury, poisoning and procedural complications | 2 | 284.15 (66.18-1220.06) |
| Encephalitis viral | Infections and infestations | 2 | 9.59 (2.39-38.45) |
| Infusion site ulcer | General disorders and administration site conditions | 2 | 142.07 (34.33-587.93) |
| Tracheo-oesophageal fistula | Congenital, familial and genetic disorders | 2 | 17.92 (4.46-71.96) |
| Oncologic complication | Neoplasms benign, malignant and unspecified (incl cysts and polyps) | 2 | 10.1 (2.52-40.47) |
| Twin pregnancy | Pregnancy, puerperium and perinatal conditions | 2 | 15.3 (3.81-61.4) |
| Myelodysplastic syndrome transformation | Neoplasms benign, malignant and unspecified (incl cysts and polyps) | 2 | 42.32 (10.48-170.93) |
| Metastases to pleura | Neoplasms benign, malignant and unspecified (incl cysts and polyps) | 2 | 10.67 (2.66-42.79) |
| Micrognathia | Congenital, familial and genetic disorders | 2 | 22.35 (5.56-89.84) |
| Neuromuscular pain | Nervous system disorders | 2 | 153 (36.87-634.9) |
| Adrenal mass | Endocrine disorders | 2 | 14.63 (3.64-58.69) |
| Drug monitoring procedure incorrectly performed | Injury, poisoning and procedural complications | 2 | 12.64 (3.15-50.71) |
| Application site mass | General disorders and administration site conditions | 2 | 28.41 (7.06-114.39) |
| Sweat gland tumour | Neoplasms benign, malignant and unspecified (incl cysts and polyps) | 1 | 23.87 (3.34-170.81) |
| Ischaemic enteritis | Gastrointestinal disorders | 1 | 49.73 (6.89-358.94) |
| Thymus enlargement | Blood and lymphatic system disorders | 1 | 57.38 (7.93-415.23) |
| Vaginal perforation | Reproductive system and breast disorders | 1 | 106.55 (14.48-784.24) |
| Puncture site swelling | General disorders and administration site conditions | 1 | 124.31 (16.79-920.62) |
| Pneumatosis | General disorders and administration site conditions | 1 | 14.28 (2-101.83) |
| Breast angiosarcoma | Neoplasms benign, malignant and unspecified (incl cysts and polyps) | 1 | 198.9 (26.15-1512.76) |
| Biliary ischaemia | Hepatobiliary disorders | 1 | 331.5 (41.46-2650.79) |
| Foetal cardiac disorder | Pregnancy, puerperium and perinatal conditions | 1 | 25.72 (3.59-184.17) |
| Cardiac function test abnormal | Investigations | 1 | 19.37 (2.71-138.43) |
| Delayed puberty | Endocrine disorders | 1 | 17.76 (2.49-126.83) |
| Tendon calcification | Musculoskeletal and connective tissue disorders | 1 | 36.83 (5.12-264.73) |
| Bladder fibrosis | Renal and urinary disorders | 1 | 165.75 (22.06-1245.63) |
| Neuroectodermal neoplasm | Neoplasms benign, malignant and unspecified (incl cysts and polyps) | 1 | 331.5 (41.46-2650.79) |
| Metabolic function test abnormal | Investigations | 1 | 21.46 (3-153.48) |
| Hepatitis chronic active | Hepatobiliary disorders | 1 | 63.48 (8.75-460.35) |
| Pericardial disease | Cardiac disorders | 1 | 19.63 (2.75-140.27) |
| Anastomotic stenosis | Injury, poisoning and procedural complications | 1 | 19.13 (2.68-136.65) |
| Catheter site bruise | General disorders and administration site conditions | 1 | 20.72 (2.9-148.11) |
| Congenital nose malformation | Congenital, familial and genetic disorders | 1 | 15.38 (2.16-109.75) |
| NUT midline carcinoma | Neoplasms benign, malignant and unspecified (incl cysts and polyps) | 1 | 745.88 (77.58-7171.4) |
| Electrocardiogram ST-T change | Investigations | 1 | 16.39 (2.3-117.02) |
| JC virus CSF test positive | Investigations | 1 | 72.77 (10-529.39) |
| Radiation fibrosis | Injury, poisoning and procedural complications | 1 | 90.41 (12.35-661.71) |
| Rhabdomyosarcoma | Neoplasms benign, malignant and unspecified (incl cysts and polyps) | 1 | 28.97 (4.04-207.64) |
| Left ventricular end-diastolic pressure increased | Investigations | 1 | 28.41 (3.96-203.65) |
| Injection site phlebitis | General disorders and administration site conditions | 1 | 32.43 (4.52-232.73) |
| Capillary disorder | Vascular disorders | 1 | 22.6 (3.16-161.68) |
| Synovial sarcoma | Neoplasms benign, malignant and unspecified (incl cysts and polyps) | 1 | 45.9 (6.37-330.9) |
| Reproductive toxicity | Reproductive system and breast disorders | 1 | 93.24 (12.73-683.05) |
| Nasal herpes | Infections and infestations | 1 | 21.62 (3.02-154.6) |
| Arachnoid cyst | Nervous system disorders | 1 | 18.3 (2.56-130.74) |
| Diffuse large B-cell lymphoma stage IV | Neoplasms benign, malignant and unspecified (incl cysts and polyps) | 1 | 20.58 (2.88-147.08) |
| Vessel puncture site pain | General disorders and administration site conditions | 1 | 55.25 (7.64-399.56) |
| Administration site reaction | General disorders and administration site conditions | 1 | 14.48 (2.03-103.32) |
| Metastases to breast | Neoplasms benign, malignant and unspecified (incl cysts and polyps) | 1 | 17.45 (2.44-124.59) |
| Gallbladder cholesterolosis | Hepatobiliary disorders | 1 | 58.5 (8.08-423.53) |
| Musculoskeletal deformity | Musculoskeletal and connective tissue disorders | 1 | 15.79 (2.21-112.66) |
| Intestinal adenocarcinoma | Neoplasms benign, malignant and unspecified (incl cysts and polyps) | 1 | 26.88 (3.75-192.54) |
| Failure to anastomose | Injury, poisoning and procedural complications | 1 | 57.38 (7.93-415.23) |
| Blood follicle stimulating hormone increased | Investigations | 1 | 22.27 (3.11-159.25) |
| Dandy-Walker syndrome | Congenital, familial and genetic disorders | 1 | 20.3 (2.84-145.07) |
| Hyponatraemic seizure | Nervous system disorders | 1 | 15.46 (2.17-110.32) |
| Vomiting psychogenic | Psychiatric disorders | 1 | 426.22 (51.31-3540.68) |
| Peritoneal mesothelioma malignant | Neoplasms benign, malignant and unspecified (incl cysts and polyps) | 1 | 110.5 (14.99-814.4) |
| Cystitis-like symptom | Renal and urinary disorders | 1 | 93.24 (12.73-683.05) |
| Contracted bladder | Renal and urinary disorders | 1 | 56.29 (7.78-407.24) |
| Prostatitis tuberculous | Infections and infestations | 1 | 596.71 (66.69-5339.3) |
| Vascular dissection | Vascular disorders | 1 | 175.5 (23.27-1323.53) |
| Inferior vena cava syndrome | Vascular disorders | 1 | 59.67 (8.24-432.17) |
| Hallucination, synaesthetic | Psychiatric disorders | 1 | 102.88 (14-756.23) |
| Immune-mediated hypophysitis | Endocrine disorders | 1 | 33.52 (4.67-240.67) |
| Malassezia infection | Infections and infestations | 1 | 106.55 (14.48-784.24) |
| Ventricular failure | Cardiac disorders | 1 | 18.42 (2.58-131.56) |
| Metastases to uterus | Neoplasms benign, malignant and unspecified (incl cysts and polyps) | 1 | 48.91 (6.78-352.96) |
| Chordae tendinae rupture | Cardiac disorders | 1 | 63.48 (8.75-460.35) |
| Cardiac cirrhosis | Hepatobiliary disorders | 1 | 58.5 (8.08-423.53) |
| Deficiency anaemia | Blood and lymphatic system disorders | 1 | 57.38 (7.93-415.23) |
| Reflux laryngitis | Respiratory, thoracic and mediastinal disorders | 1 | 15.54 (2.18-110.89) |
| Metastases to soft tissue | Neoplasms benign, malignant and unspecified (incl cysts and polyps) | 1 | 20.3 (2.84-145.07) |
| Lymphatic obstruction | Blood and lymphatic system disorders | 1 | 67.81 (9.34-492.46) |
| Blood stem cell harvest failure | Injury, poisoning and procedural complications | 1 | 67.81 (9.34-492.46) |
| Alveolar soft part sarcoma | Neoplasms benign, malignant and unspecified (incl cysts and polyps) | 1 | 87.75 (12-641.66) |
| Neonatal pneumonia | Infections and infestations | 1 | 21.46 (3-153.48) |
| BRAF gene mutation | Congenital, familial and genetic disorders | 1 | 42.62 (5.92-306.93) |
| Soft tissue inflammation | General disorders and administration site conditions | 1 | 21.46 (3-153.48) |
| Muscle oedema | Musculoskeletal and connective tissue disorders | 1 | 23.31 (3.26-166.77) |
| Lymphocyte percentage increased | Investigations | 1 | 19.25 (2.69-137.53) |
| Hepatobiliary procedural complication | Injury, poisoning and procedural complications | 1 | 157.03 (20.96-1176.4) |
| Laryngopharyngitis | Infections and infestations | 1 | 85.24 (11.67-622.79) |
| Liver carcinoma ruptured | Neoplasms benign, malignant and unspecified (incl cysts and polyps) | 1 | 25.72 (3.59-184.17) |
| Liposarcoma recurrent | Neoplasms benign, malignant and unspecified (incl cysts and polyps) | 1 | 331.5 (41.46-2650.79) |
| Somatic hallucination | Psychiatric disorders | 1 | 80.64 (11.05-588.2) |
| Blood pressure difference of extremities | Investigations | 1 | 90.41 (12.35-661.71) |
| Microscopic polyangiitis | Vascular disorders | 1 | 18.76 (2.63-134.05) |
| Pneumonitis chemical | Injury, poisoning and procedural complications | 1 | 16.3 (2.28-116.38) |
| Congenital foot malformation | Congenital, familial and genetic disorders | 1 | 16.04 (2.25-114.49) |
| Application site oedema | General disorders and administration site conditions | 1 | 21.94 (3.07-156.89) |
| Aspartate aminotransferase | Investigations | 1 | 22.6 (3.16-161.68) |
| Pancreatic enlargement | Gastrointestinal disorders | 1 | 18.76 (2.63-134.05) |
| Pectus excavatum | Congenital, familial and genetic disorders | 1 | 22.78 (3.18-162.92) |
| Peripancreatic fluid collection | Injury, poisoning and procedural complications | 1 | 42.62 (5.92-306.93) |
| Cerebellar ischaemia | Nervous system disorders | 1 | 26.88 (3.75-192.54) |
| Herpes simplex hepatitis | Infections and infestations | 1 | 23.49 (3.28-168.09) |
| Chromosomal mutation | Congenital, familial and genetic disorders | 1 | 93.24 (12.73-683.05) |
| Bacillus bacteraemia | Infections and infestations | 1 | 40.32 (5.6-290.11) |
| Biloma | Hepatobiliary disorders | 1 | 45.9 (6.37-330.9) |
| Aerophagia | Gastrointestinal disorders | 1 | 31.74 (4.42-227.73) |
| Infusion site hypoaesthesia | General disorders and administration site conditions | 1 | 49.73 (6.89-358.94) |
| Tricuspid valve disease | Cardiac disorders | 1 | 15.07 (2.11-107.52) |
| Small cell lung cancer metastatic | Neoplasms benign, malignant and unspecified (incl cysts and polyps) | 1 | 17.65 (2.47-126.07) |
| Anovulatory cycle | Endocrine disorders | 1 | 27.63 (3.86-197.94) |
| Ameloblastoma | Neoplasms benign, malignant and unspecified (incl cysts and polyps) | 1 | 80.64 (11.05-588.2) |
| Haemorrhagic ascites | Gastrointestinal disorders | 1 | 21.94 (3.07-156.89) |
| Electrocardiogram ST-T segment depression | Investigations | 1 | 142.07 (19.07-1058.73) |
| Lymphocytic leukaemia | Neoplasms benign, malignant and unspecified (incl cysts and polyps) | 1 | 14.34 (2.01-102.32) |
| Dermatochalasis | Eye disorders | 1 | 27.88 (3.89-199.81) |
| Factor I deficiency | Congenital, familial and genetic disorders | 1 | 331.5 (41.46-2650.79) |
| Tumour perforation | Neoplasms benign, malignant and unspecified (incl cysts and polyps) | 1 | 19 (2.66-135.77) |
| Ventricular tachyarrhythmia | Cardiac disorders | 1 | 14.48 (2.03-103.32) |
| Peripheral nerve palsy | Nervous system disorders | 1 | 54.25 (7.5-392.16) |
| Enteropathy-associated T-cell lymphoma | Neoplasms benign, malignant and unspecified (incl cysts and polyps) | 1 | 87.75 (12-641.66) |
| Aortic elongation | Vascular disorders | 1 | 76.5 (10.5-557.25) |
| Biliary cirrhosis | Hepatobiliary disorders | 1 | 32.79 (4.57-235.32) |
| Neutropenia neonatal | Blood and lymphatic system disorders | 1 | 30.14 (4.2-216.11) |
| Therapeutic embolisation | Surgical and medical procedures | 1 | 14.7 (2.06-104.86) |
| Eosinopenia | Blood and lymphatic system disorders | 1 | 40.32 (5.6-290.11) |
| Fracture reduction | Surgical and medical procedures | 1 | 186.47 (24.63-1411.82) |
| Anal erythema | Gastrointestinal disorders | 1 | 23.87 (3.34-170.81) |
| Vascular encephalopathy | Nervous system disorders | 1 | 20.16 (2.82-144.08) |
| Leiomyosarcoma recurrent | Neoplasms benign, malignant and unspecified (incl cysts and polyps) | 1 | 213.11 (27.88-1629.25) |
| Wound evisceration | Injury, poisoning and procedural complications | 1 | 102.88 (14-756.23) |
| Leukaemic infiltration | Neoplasms benign, malignant and unspecified (incl cysts and polyps) | 1 | 43.88 (6.09-316.09) |
| Cryptococcal cutaneous infection | Infections and infestations | 1 | 35.52 (4.94-255.16) |
| Metastases to heart | Neoplasms benign, malignant and unspecified (incl cysts and polyps) | 1 | 18.3 (2.56-130.74) |
| Anal sphincter atony | Gastrointestinal disorders | 1 | 15.46 (2.17-110.32) |
| Tympanic membrane disorder | Ear and labyrinth disorders | 1 | 16.3 (2.28-116.38) |
| Antineutrophil cytoplasmic antibody increased | Investigations | 1 | 31.74 (4.42-227.73) |
| Chest wall necrosis | Musculoskeletal and connective tissue disorders | 1 | 426.22 (51.31-3540.68) |
| Gastrointestinal anastomotic leak | Injury, poisoning and procedural complications | 1 | 14.28 (2-101.83) |
| Anhidrosis | Skin and subcutaneous tissue disorders | 1 | 18.88 (2.64-134.91) |
| Blood smear test abnormal | Investigations | 1 | 96.24 (13.12-705.82) |
| Pharyngotonsillitis | Infections and infestations | 1 | 22.1 (3.09-158.06) |
| Neonatal hypotension | Vascular disorders | 1 | 16.95 (2.37-121.03) |
