# Supplementary material for: Epirubicin and gait apraxia: a real-world data analysis of the FDA Adverse Event Reporting System database
Source: Front Pharmacol. 2023 Sep 14;14:1249845. doi: 10.3389/fphar.2023.1249845 (PMC10536159; doi:10.3389/fphar.2023.1249845)
Supplement: Supplementary file 4 [file Table3.docx]

Supplementary Table S3 The comprehensive detailed information of all AEs at the PTs level identified by PRR algorithms.

| Preferred Terms | SOC | Case Number | PRR (95% two side CI) |
| --- | --- | --- | --- |
| Nausea | Gastrointestinal disorders | 300 | 2.08 (1.86-2.32) |
| Fatigue | General disorders and administration site conditions | 184 | 1.21 (1.05-1.39) |
| Diarrhoea | Gastrointestinal disorders | 162 | 1.3 (1.12-1.52) |
| Hepatic artery stenosis | Hepatobiliary disorders | 8 | 554.89 (260.95-1179.95) |
| Endocardial fibrosis | Cardiac disorders | 3 | 497.09 (146.44-1687.34) |
| Gait apraxia | Nervous system disorders | 3 | 497.09 (146.44-1687.34) |
| Alopecia | Skin and subcutaneous tissue disorders | 98 | 2.23 (1.83-2.72) |
| Cardiac perfusion defect | Cardiac disorders | 3 | 372.82 (112.28-1237.93) |
| Decreased appetite | Metabolism and nutrition disorders | 90 | 2.03 (1.65-2.49) |
| Constipation | Gastrointestinal disorders | 76 | 1.9 (1.52-2.38) |
| Hepatic artery occlusion | Hepatobiliary disorders | 3 | 344.14 (104.18-1136.85) |
| Administration site oedema | General disorders and administration site conditions | 4 | 313.95 (112.07-879.51) |
| Paraesthesia | Nervous system disorders | 62 | 2.14 (1.67-2.74) |
| Post embolisation syndrome | Injury, poisoning and procedural complications | 4 | 209.3 (75.95-576.75) |
| Abdominal pain | Gastrointestinal disorders | 58 | 1.41 (1.09-1.82) |
| Chest pain | General disorders and administration site conditions | 51 | 1.66 (1.26-2.19) |
| General physical health deterioration | General disorders and administration site conditions | 49 | 2.51 (1.9-3.32) |
| Myalgia | Musculoskeletal and connective tissue disorders | 47 | 1.56 (1.18-2.08) |
| Malignant neoplasm progression | Neoplasms benign, malignant and unspecified (incl cysts and polyps) | 47 | 2.27 (1.71-3.02) |
| Menopausal disorder | Reproductive system and breast disorders | 3 | 149.13 (46.78-475.42) |
| Maternal exposure during pregnancy | Injury, poisoning and procedural complications | 42 | 2.4 (1.77-3.25) |
| Gastrointestinal disorder | Gastrointestinal disorders | 38 | 2.58 (1.88-3.55) |
| Granulocyte count decreased | Investigations | 26 | 144.95 (97.82-214.77) |
| Chest discomfort | General disorders and administration site conditions | 37 | 2.04 (1.48-2.81) |
| Oropharyngeal pain | Respiratory, thoracic and mediastinal disorders | 37 | 2.04 (1.48-2.82) |
| Intestinal atresia | Congenital, familial and genetic disorders | 3 | 133.55 (42.01-424.52) |
| Breast cancer | Neoplasms benign, malignant and unspecified (incl cysts and polyps) | 35 | 1.62 (1.17-2.26) |
| Biliary fistula | Hepatobiliary disorders | 3 | 124.27 (39.16-394.36) |
| Chills | General disorders and administration site conditions | 35 | 1.67 (1.2-2.33) |
| Sepsis | Infections and infestations | 34 | 1.7 (1.21-2.38) |
| Acute cutaneous lupus erythematosus | Skin and subcutaneous tissue disorders | 4 | 114.71 (42.26-311.35) |
| Palpitations | Cardiac disorders | 33 | 1.59 (1.13-2.23) |
| Dysphagia | Gastrointestinal disorders | 32 | 1.93 (1.36-2.73) |
| Dysgeusia | Nervous system disorders | 31 | 2.31 (1.62-3.28) |
| Platelet count decreased | Investigations | 30 | 1.53 (1.07-2.19) |
| Respiratory failure | Respiratory, thoracic and mediastinal disorders | 30 | 2.44 (1.71-3.49) |
| Miller Fisher syndrome | Nervous system disorders | 4 | 105.58 (38.96-286.13) |
| Right atrial dilatation | Cardiac disorders | 7 | 100.86 (47.5-214.16) |
| Pulmonary embolism | Respiratory, thoracic and mediastinal disorders | 29 | 1.94 (1.35-2.79) |
| Abdominal distension | Gastrointestinal disorders | 28 | 1.5 (1.04-2.17) |
| Atrial fibrillation | Cardiac disorders | 26 | 1.47 (1-2.16) |
| Hot flush | Vascular disorders | 26 | 1.93 (1.32-2.84) |
| Tachycardia | Cardiac disorders | 25 | 1.66 (1.12-2.45) |
| Productive cough | Respiratory, thoracic and mediastinal disorders | 23 | 2.5 (1.66-3.75) |
| Oedema | General disorders and administration site conditions | 23 | 2.51 (1.67-3.77) |
| Disturbance in attention | Nervous system disorders | 22 | 2.26 (1.49-3.43) |
| Discomfort | General disorders and administration site conditions | 22 | 1.93 (1.27-2.93) |
| Deep vein thrombosis | Vascular disorders | 22 | 2.17 (1.43-3.3) |
| Cognitive disorder | Nervous system disorders | 22 | 2.55 (1.68-3.87) |
| Stomatitis | Gastrointestinal disorders | 21 | 1.82 (1.18-2.78) |
| Administration site extravasation | General disorders and administration site conditions | 19 | 88.54 (56.13-139.69) |
| Xerophthalmia | Eye disorders | 8 | 85.52 (42.36-172.66) |
| Cardio-respiratory arrest | Cardiac disorders | 18 | 2.72 (1.71-4.31) |
| Dyspnoea exertional | Respiratory, thoracic and mediastinal disorders | 18 | 2.48 (1.56-3.94) |
| Inflammation | General disorders and administration site conditions | 17 | 1.82 (1.13-2.93) |
| Placental disorder | Pregnancy, puerperium and perinatal conditions | 10 | 78.08 (41.68-146.25) |
| Colitis | Gastrointestinal disorders | 17 | 2.5 (1.55-4.01) |
| Neutrophil percentage decreased | Investigations | 4 | 71.87 (26.66-193.72) |
| Haematuria | Renal and urinary disorders | 16 | 2.42 (1.48-3.94) |
| Metabolic acidosis | Metabolism and nutrition disorders | 16 | 2.86 (1.75-4.66) |
| Cardiac dysfunction | Cardiac disorders | 49 | 70.26 (52.96-93.22) |
| Lower respiratory tract infection | Infections and infestations | 15 | 1.75 (1.05-2.9) |
| Bladder irritation | Renal and urinary disorders | 8 | 64.14 (31.85-129.18) |
| Refractory cancer | Neoplasms benign, malignant and unspecified (incl cysts and polyps) | 3 | 63.91 (20.37-200.54) |
| Blood bilirubin increased | Investigations | 14 | 3.36 (1.99-5.68) |
| Metastases to thorax | Neoplasms benign, malignant and unspecified (incl cysts and polyps) | 3 | 60.46 (19.28-189.58) |
| Hepatotoxicity | Hepatobiliary disorders | 13 | 3.18 (1.85-5.48) |
| Hypophagia | Metabolism and nutrition disorders | 13 | 2.76 (1.6-4.76) |
| Ascites | Gastrointestinal disorders | 13 | 2.5 (1.45-4.3) |
| Glossodynia | Gastrointestinal disorders | 12 | 3.41 (1.94-6.01) |
| C-reactive protein increased | Investigations | 12 | 1.92 (1.09-3.38) |
| Ear pain | Ear and labyrinth disorders | 12 | 3.41 (1.94-6) |
| Liver function test abnormal | Investigations | 11 | 2.8 (1.55-5.05) |
| Clostridium difficile infection | Infections and infestations | 11 | 2.3 (1.27-4.16) |
| Acute respiratory failure | Respiratory, thoracic and mediastinal disorders | 11 | 3.09 (1.71-5.59) |
| Red blood cell count decreased | Investigations | 11 | 2.08 (1.15-3.76) |
| Acute respiratory distress syndrome | Respiratory, thoracic and mediastinal disorders | 10 | 3.5 (1.88-6.5) |
| Presyncope | Nervous system disorders | 10 | 2.22 (1.2-4.13) |
| Sensory disturbance | Nervous system disorders | 10 | 3.36 (1.81-6.25) |
| Pseudocirrhosis | Hepatobiliary disorders | 4 | 54.48 (20.27-146.42) |
| Merycism | Psychiatric disorders | 3 | 53.9 (17.21-168.81) |
| Gastritis | Gastrointestinal disorders | 10 | 2.29 (1.23-4.26) |
| Impaired work ability | Social circumstances | 10 | 2.54 (1.37-4.73) |
| Dilated cardiomyopathy | Cardiac disorders | 43 | 51.65 (38.23-69.79) |
| Acute myocardial infarction | Cardiac disorders | 10 | 1.97 (1.06-3.66) |
| Pulmonary hypertension | Respiratory, thoracic and mediastinal disorders | 9 | 2.45 (1.28-4.72) |
| Pulmonary fibrosis | Respiratory, thoracic and mediastinal disorders | 9 | 2.98 (1.55-5.73) |
| Increased appetite | Metabolism and nutrition disorders | 9 | 3.05 (1.59-5.87) |
| Catheter site related reaction | General disorders and administration site conditions | 5 | 50.21 (20.75-121.5) |
| Hepatic steatosis | Hepatobiliary disorders | 9 | 3.09 (1.61-5.94) |
| Photophobia | Eye disorders | 9 | 2.77 (1.44-5.31) |
| Circulatory collapse | Vascular disorders | 9 | 3.28 (1.71-6.31) |
| Haematemesis | Gastrointestinal disorders | 9 | 2.06 (1.07-3.97) |
| Pericardial effusion | Cardiac disorders | 9 | 2.26 (1.18-4.34) |
| Metastases to the mediastinum | Neoplasms benign, malignant and unspecified (incl cysts and polyps) | 4 | 49.5 (18.43-132.95) |
| Jaundice | Hepatobiliary disorders | 9 | 2.17 (1.13-4.16) |
| Subclavian vein thrombosis | Vascular disorders | 8 | 45.1 (22.44-90.64) |
| Carbohydrate antigen 15-3 increased | Investigations | 6 | 43.65 (19.5-97.7) |
| Cardiogenic shock | Cardiac disorders | 8 | 3.44 (1.72-6.88) |
| Joint range of motion decreased | Musculoskeletal and connective tissue disorders | 8 | 3.53 (1.76-7.06) |
| Psychotic behaviour | Psychiatric disorders | 7 | 43.05 (20.42-90.76) |
| Neutropenic sepsis | Infections and infestations | 55 | 42.82 (32.83-55.84) |
| Breast cancer recurrent | Neoplasms benign, malignant and unspecified (incl cysts and polyps) | 22 | 42.58 (27.96-64.83) |
| Myocardial oedema | Cardiac disorders | 3 | 42.41 (13.57-132.52) |
| Soft tissue sarcoma | Neoplasms benign, malignant and unspecified (incl cysts and polyps) | 4 | 42.31 (15.77-113.49) |
| Generalised tonic-clonic seizure | Nervous system disorders | 8 | 2.06 (1.03-4.12) |
| Retching | Gastrointestinal disorders | 8 | 2.08 (1.04-4.17) |
| Quality of life decreased | Investigations | 7 | 3.14 (1.5-6.6) |
| Muscle atrophy | Musculoskeletal and connective tissue disorders | 7 | 3.59 (1.71-7.53) |
| Toxic skin eruption | Skin and subcutaneous tissue disorders | 7 | 4.19 (2-8.79) |
| Paraesthesia oral | Gastrointestinal disorders | 7 | 2.78 (1.32-5.83) |
| Pericarditis | Cardiac disorders | 7 | 3 (1.43-6.29) |
| Allodynia | Nervous system disorders | 5 | 38.34 (15.87-92.61) |
| Acute hepatic failure | Hepatobiliary disorders | 7 | 2.92 (1.39-6.12) |
| Myelodysplastic syndrome | Neoplasms benign, malignant and unspecified (incl cysts and polyps) | 7 | 2.91 (1.39-6.1) |
| Soft tissue necrosis | Musculoskeletal and connective tissue disorders | 4 | 36.94 (13.78-98.99) |
| Blood magnesium decreased | Investigations | 6 | 3.7 (1.66-8.24) |
| Appendicitis | Infections and infestations | 6 | 3.75 (1.68-8.35) |
| Supraventricular tachycardia | Cardiac disorders | 6 | 4 (1.79-8.9) |
| Myocardial fibrosis | Cardiac disorders | 5 | 35.76 (14.81-86.36) |
| Aortic dilatation | Vascular disorders | 6 | 35.3 (15.79-78.92) |
| Hypercreatininaemia | Metabolism and nutrition disorders | 3 | 35.23 (11.29-109.94) |
| Acute leukaemia | Neoplasms benign, malignant and unspecified (incl cysts and polyps) | 8 | 33.84 (16.86-67.93) |
| Inappropriate antidiuretic hormone secretion | Endocrine disorders | 6 | 3.56 (1.6-7.92) |
| Infusion site pain | General disorders and administration site conditions | 6 | 2.38 (1.07-5.3) |
| Temperature intolerance | General disorders and administration site conditions | 6 | 3.21 (1.44-7.14) |
| Myocardial ischaemia | Cardiac disorders | 6 | 3.73 (1.68-8.31) |
| Amnestic disorder | Nervous system disorders | 3 | 33.39 (10.7-104.16) |
| Arterial thrombosis | Vascular disorders | 10 | 32.85 (17.62-61.24) |
| Myelosuppression | Blood and lymphatic system disorders | 121 | 32.11 (26.87-38.37) |
| Tongue coated | Gastrointestinal disorders | 8 | 32.07 (15.98-64.35) |
| Hyperbilirubinaemia | Hepatobiliary disorders | 6 | 3.27 (1.47-7.28) |
| Hyperamylasaemia | Metabolism and nutrition disorders | 3 | 31.96 (10.25-99.67) |
| Cardiotoxicity | Cardiac disorders | 53 | 31.81 (24.28-41.67) |
| Tachypnoea | Respiratory, thoracic and mediastinal disorders | 6 | 2.62 (1.17-5.82) |
| Flank pain | Musculoskeletal and connective tissue disorders | 5 | 3.04 (1.26-7.3) |
| Aortitis | Vascular disorders | 4 | 30.67 (11.45-82.11) |
| Intestinal perforation | Gastrointestinal disorders | 5 | 2.58 (1.07-6.19) |
| Tumour lysis syndrome | Metabolism and nutrition disorders | 5 | 3.11 (1.29-7.47) |
| Urosepsis | Infections and infestations | 5 | 2.96 (1.23-7.12) |
| Hyperlipasaemia | Metabolism and nutrition disorders | 3 | 28.68 (9.2-89.39) |
| Sexual dysfunction | Reproductive system and breast disorders | 5 | 2.48 (1.03-5.95) |
| Aortic thrombosis | Vascular disorders | 5 | 27.93 (11.58-67.36) |
| Disseminated intravascular coagulation | Blood and lymphatic system disorders | 5 | 2.44 (1.01-5.86) |
| Oesophagitis | Gastrointestinal disorders | 5 | 3.07 (1.28-7.37) |
| Abdominal sepsis | Infections and infestations | 4 | 27.55 (10.3-73.73) |
| Infusion site erythema | General disorders and administration site conditions | 5 | 3.25 (1.35-7.81) |
| Hepatic function abnormal | Hepatobiliary disorders | 161 | 27.17 (23.29-31.7) |
| Papule | Skin and subcutaneous tissue disorders | 5 | 3.81 (1.58-9.15) |
| Initial insomnia | Psychiatric disorders | 5 | 3.29 (1.37-7.9) |
| Acute coronary syndrome | Cardiac disorders | 5 | 3.51 (1.46-8.45) |
| Anastomotic leak | Injury, poisoning and procedural complications | 3 | 26.87 (8.62-83.73) |
| Iatrogenic injury | Injury, poisoning and procedural complications | 3 | 26.55 (8.52-82.73) |
| Dilatation ventricular | Cardiac disorders | 5 | 26.12 (10.83-62.98) |
| Febrile bone marrow aplasia | Blood and lymphatic system disorders | 20 | 25.78 (16.61-40.02) |
| Cardiomegaly | Cardiac disorders | 5 | 3 (1.25-7.2) |
| Subacute cutaneous lupus erythematosus | Skin and subcutaneous tissue disorders | 10 | 24.85 (13.34-46.3) |
| Coronary artery thrombosis | Cardiac disorders | 6 | 24.82 (11.12-55.42) |
| Bone marrow failure | Blood and lymphatic system disorders | 105 | 24.74 (20.44-29.95) |
| Ovarian cancer | Neoplasms benign, malignant and unspecified (incl cysts and polyps) | 5 | 2.67 (1.11-6.43) |
| Troponin T increased | Investigations | 5 | 23.75 (9.85-57.24) |
| Hyperammonaemia | Metabolism and nutrition disorders | 4 | 4.64 (1.74-12.36) |
| Hepatitis acute | Hepatobiliary disorders | 4 | 3.88 (1.46-10.34) |
| Atrial septal defect | Congenital, familial and genetic disorders | 4 | 2.77 (1.04-7.39) |
| Erythema multiforme | Skin and subcutaneous tissue disorders | 4 | 2.79 (1.05-7.43) |
| Embolism | Vascular disorders | 4 | 2.9 (1.09-7.74) |
| Neutropenic infection | Infections and infestations | 3 | 23.06 (7.41-71.81) |
| Panic reaction | Psychiatric disorders | 4 | 3.11 (1.17-8.28) |
| Rash vesicular | Skin and subcutaneous tissue disorders | 4 | 4.91 (1.84-13.08) |
| Troponin I increased | Investigations | 6 | 22.65 (10.15-50.56) |
| Lymphoedema | Vascular disorders | 4 | 3.14 (1.18-8.36) |
| Hydrothorax | Respiratory, thoracic and mediastinal disorders | 4 | 22.51 (8.42-60.19) |
| Non-Hodgkin's lymphoma | Neoplasms benign, malignant and unspecified (incl cysts and polyps) | 4 | 3.69 (1.38-9.83) |
| Sunburn | Injury, poisoning and procedural complications | 4 | 2.88 (1.08-7.67) |
| Small for dates baby | Pregnancy, puerperium and perinatal conditions | 4 | 4.54 (1.7-12.1) |
| Extravasation | General disorders and administration site conditions | 16 | 22.43 (13.72-36.66) |
| Enteritis | Gastrointestinal disorders | 4 | 3.39 (1.27-9.04) |
| Second primary malignancy | Neoplasms benign, malignant and unspecified (incl cysts and polyps) | 43 | 22.05 (16.34-29.75) |
| Ventricular hypokinesia | Cardiac disorders | 11 | 22.05 (12.19-39.89) |
| Ventricular extrasystoles | Cardiac disorders | 4 | 2.77 (1.04-7.39) |
| Visual field defect | Eye disorders | 4 | 3.36 (1.26-8.95) |
| Pulmonary toxicity | Respiratory, thoracic and mediastinal disorders | 4 | 3.4 (1.27-9.05) |
| Klebsiella infection | Infections and infestations | 4 | 4.54 (1.7-12.12) |
| N-terminal prohormone brain natriuretic peptide increased | Investigations | 6 | 21.43 (9.6-47.83) |
| Agranulocytosis | Blood and lymphatic system disorders | 63 | 21.01 (16.41-26.9) |
| Cholecystitis acute | Hepatobiliary disorders | 4 | 4.96 (1.86-13.23) |
| Cardiac death | General disorders and administration site conditions | 4 | 20.89 (7.82-55.85) |
| Ventricular dysfunction | Cardiac disorders | 5 | 20.77 (8.62-50.04) |
| Anal ulcer | Gastrointestinal disorders | 3 | 20.57 (6.61-64.02) |
| Left ventricular failure | Cardiac disorders | 3 | 5.5 (1.77-17.06) |
| Bile duct stenosis | Hepatobiliary disorders | 4 | 20.22 (7.57-54.05) |
| Acute promyelocytic leukaemia | Neoplasms benign, malignant and unspecified (incl cysts and polyps) | 3 | 20.02 (6.43-62.29) |
| Tongue ulceration | Gastrointestinal disorders | 3 | 4.98 (1.61-15.47) |
| Systemic infection | Infections and infestations | 3 | 6.16 (1.99-19.13) |
| Guillain-Barre syndrome | Nervous system disorders | 3 | 3.83 (1.23-11.87) |
| Cutaneous symptom | Skin and subcutaneous tissue disorders | 3 | 19.93 (6.4-62.01) |
| Hypertransaminasaemia | Hepatobiliary disorders | 21 | 19.8 (12.89-30.39) |
| Organising pneumonia | Respiratory, thoracic and mediastinal disorders | 3 | 3.35 (1.08-10.38) |
| Hypovolaemic shock | Vascular disorders | 3 | 3.47 (1.12-10.78) |
| Ileus paralytic | Gastrointestinal disorders | 3 | 4.65 (1.5-14.43) |
| Blood lactic acid increased | Investigations | 3 | 3.34 (1.08-10.36) |
| Hepatitis B | Infections and infestations | 19 | 19.16 (12.21-30.07) |
| Menopause | Social circumstances | 8 | 19.04 (9.5-38.15) |
| Ocular toxicity | Eye disorders | 3 | 19 (6.11-59.11) |
| Oral fungal infection | Infections and infestations | 3 | 6.16 (1.98-19.11) |
| Neutropenic colitis | Gastrointestinal disorders | 7 | 18.66 (8.88-39.22) |
| Atypical haemolytic uraemic syndrome | Blood and lymphatic system disorders | 3 | 18.56 (5.97-57.75) |
| Infusion site discolouration | General disorders and administration site conditions | 3 | 18.53 (5.95-57.63) |
| Language disorder | Nervous system disorders | 3 | 5.28 (1.7-16.38) |
| Hypertriglyceridaemia | Metabolism and nutrition disorders | 3 | 3.16 (1.02-9.79) |
| Jugular vein thrombosis | Vascular disorders | 5 | 18.37 (7.62-44.23) |
| Polydipsia | Metabolism and nutrition disorders | 3 | 4.8 (1.55-14.9) |
| Arteriosclerosis coronary artery | Cardiac disorders | 3 | 3.42 (1.1-10.62) |
| Aortic valve incompetence | Cardiac disorders | 3 | 5.68 (1.83-17.62) |
| Kidney enlargement | Renal and urinary disorders | 3 | 17.51 (5.63-54.46) |
| Left ventricular dysfunction | Cardiac disorders | 20 | 17.17 (11.06-26.63) |
| Hepatic lesion | Hepatobiliary disorders | 13 | 17.08 (9.91-29.45) |
| Liver injury | Hepatobiliary disorders | 61 | 16.56 (12.89-21.29) |
| Mental fatigue | Psychiatric disorders | 5 | 16.44 (6.83-39.59) |
| Papilloedema | Eye disorders | 3 | 3.93 (1.27-12.21) |
| Epigastric discomfort | Gastrointestinal disorders | 3 | 3.9 (1.26-12.11) |
| Axillary mass | Musculoskeletal and connective tissue disorders | 3 | 15.73 (5.06-48.9) |
| Respiratory alkalosis | Respiratory, thoracic and mediastinal disorders | 4 | 15.62 (5.85-41.71) |
| Lymphangiosis carcinomatosa | Neoplasms benign, malignant and unspecified (incl cysts and polyps) | 3 | 15.53 (5-48.3) |
| Biliary tract disorder | Hepatobiliary disorders | 3 | 15.17 (4.88-47.15) |
| Acute pulmonary oedema | Respiratory, thoracic and mediastinal disorders | 13 | 15.16 (8.8-26.14) |
| Dysentery | Infections and infestations | 6 | 14.91 (6.69-33.25) |
| Pancreatitis necrotising | Gastrointestinal disorders | 5 | 14.9 (6.19-35.86) |
| Diastolic dysfunction | Cardiac disorders | 7 | 14.81 (7.05-31.11) |
| Right ventricular dysfunction | Cardiac disorders | 3 | 14.6 (4.7-45.38) |
| Acute lymphocytic leukaemia | Neoplasms benign, malignant and unspecified (incl cysts and polyps) | 8 | 14.59 (7.29-29.22) |
| Metastases to lymph nodes | Neoplasms benign, malignant and unspecified (incl cysts and polyps) | 17 | 14.41 (8.95-23.2) |
| Adenocarcinoma gastric | Neoplasms benign, malignant and unspecified (incl cysts and polyps) | 3 | 14.18 (4.56-44.08) |
| Catheter site pain | General disorders and administration site conditions | 7 | 13.93 (6.63-29.26) |
| Cardiomyopathy | Cardiac disorders | 32 | 13.84 (9.78-19.57) |
| Metastasis | Neoplasms benign, malignant and unspecified (incl cysts and polyps) | 17 | 13.69 (8.51-22.04) |
| Biliary dilatation | Hepatobiliary disorders | 3 | 13.62 (4.38-42.33) |
| Dislocation of vertebra | Injury, poisoning and procedural complications | 3 | 13.48 (4.34-41.88) |
| Fanconi syndrome acquired | Renal and urinary disorders | 5 | 13.22 (5.49-31.82) |
| Hyperammonaemic encephalopathy | Nervous system disorders | 5 | 13.13 (5.45-31.59) |
| Menopausal symptoms | Reproductive system and breast disorders | 5 | 13.09 (5.44-31.51) |
| Ejection fraction decreased | Investigations | 36 | 12.98 (9.36-17.99) |
| Pharyngeal erythema | Respiratory, thoracic and mediastinal disorders | 5 | 12.93 (5.37-31.13) |
| Angiopathy | Vascular disorders | 11 | 12.92 (7.15-23.36) |
| Drug-induced liver injury | Hepatobiliary disorders | 75 | 12.83 (10.23-16.08) |
| Phlebitis | Vascular disorders | 10 | 12.83 (6.89-23.87) |
| Breast cancer metastatic | Neoplasms benign, malignant and unspecified (incl cysts and polyps) | 20 | 12.82 (8.27-19.88) |
| Skin hypopigmentation | Skin and subcutaneous tissue disorders | 5 | 12.67 (5.26-30.49) |
| Myocardial injury | Cardiac disorders | 4 | 12.49 (4.68-33.35) |
| Appendicitis perforated | Infections and infestations | 5 | 12.45 (5.17-29.96) |
| Skin sensitisation | Skin and subcutaneous tissue disorders | 3 | 12.36 (3.98-38.4) |
| Vena cava thrombosis | Vascular disorders | 3 | 12.27 (3.95-38.14) |
| Cardiac failure chronic | Cardiac disorders | 11 | 12.27 (6.79-22.18) |
| Adenocarcinoma | Neoplasms benign, malignant and unspecified (incl cysts and polyps) | 5 | 12.17 (5.06-29.29) |
| Hyperpyrexia | General disorders and administration site conditions | 8 | 12.06 (6.03-24.15) |
| Neutrophil count decreased | Investigations | 86 | 12.01 (9.72-14.83) |
| Multi-organ disorder | General disorders and administration site conditions | 3 | 11.95 (3.84-37.12) |
| Mucosal inflammation | General disorders and administration site conditions | 54 | 11.82 (9.05-15.43) |
| Body temperature abnormal | Investigations | 5 | 11.64 (4.84-28.01) |
| Odynophagia | Gastrointestinal disorders | 11 | 11.54 (6.39-20.86) |
| Mucosal dryness | General disorders and administration site conditions | 4 | 11.34 (4.25-30.27) |
| Catheter site erythema | General disorders and administration site conditions | 5 | 11.25 (4.68-27.08) |
| Metastases to bone | Neoplasms benign, malignant and unspecified (incl cysts and polyps) | 33 | 11.21 (7.97-15.77) |
| Mucosal disorder | General disorders and administration site conditions | 4 | 11.06 (4.14-29.51) |
| Acute myeloid leukaemia | Neoplasms benign, malignant and unspecified (incl cysts and polyps) | 29 | 11.02 (7.66-15.86) |
| Tetany | Metabolism and nutrition disorders | 3 | 10.91 (3.51-33.9) |
| Peripheral sensory neuropathy | Nervous system disorders | 11 | 10.76 (5.96-19.45) |
| Pseudomonal sepsis | Infections and infestations | 3 | 10.74 (3.46-33.37) |
| Maternal exposure timing unspecified | Injury, poisoning and procedural complications | 5 | 10.73 (4.46-25.81) |
| Cardiac failure acute | Cardiac disorders | 13 | 10.64 (6.17-18.34) |
| Nail infection | Infections and infestations | 3 | 10.58 (3.4-32.85) |
| Febrile neutropenia | Blood and lymphatic system disorders | 122 | 10.48 (8.78-12.51) |
| Neoplasm recurrence | Neoplasms benign, malignant and unspecified (incl cysts and polyps) | 6 | 10.43 (4.68-23.24) |
| Liver abscess | Infections and infestations | 6 | 10.39 (4.66-23.16) |
| Metastases to meninges | Neoplasms benign, malignant and unspecified (incl cysts and polyps) | 4 | 10.15 (3.8-27.09) |
| Malignant pleural effusion | Neoplasms benign, malignant and unspecified (incl cysts and polyps) | 3 | 10.11 (3.25-31.4) |
| Livedo reticularis | Skin and subcutaneous tissue disorders | 3 | 9.94 (3.2-30.88) |
| Mitral valve incompetence | Cardiac disorders | 14 | 9.85 (5.83-16.64) |
| Injection site hypersensitivity | General disorders and administration site conditions | 5 | 9.68 (4.02-23.28) |
| Intervertebral discitis | Infections and infestations | 3 | 9.66 (3.11-30.01) |
| Periodontitis | Infections and infestations | 3 | 9.64 (3.1-29.95) |
| Tricuspid valve incompetence | Cardiac disorders | 9 | 9.63 (5.01-18.53) |
| Polyneuropathy | Nervous system disorders | 21 | 9.63 (6.28-14.77) |
| Radiotherapy | Surgical and medical procedures | 3 | 9.45 (3.04-29.34) |
| Skin toxicity | Skin and subcutaneous tissue disorders | 9 | 9.36 (4.87-18) |
| Portal vein thrombosis | Hepatobiliary disorders | 5 | 9.26 (3.85-22.28) |
| Cerebral venous thrombosis | Nervous system disorders | 3 | 9.06 (2.92-28.12) |
| Venous thrombosis | Vascular disorders | 6 | 9.05 (4.06-20.17) |
| Lichenoid keratosis | Skin and subcutaneous tissue disorders | 3 | 8.97 (2.89-27.87) |
| Jaundice neonatal | Pregnancy, puerperium and perinatal conditions | 3 | 8.96 (2.88-27.81) |
| Gastrointestinal necrosis | Gastrointestinal disorders | 4 | 8.88 (3.33-23.7) |
| Lymphadenopathy mediastinal | Blood and lymphatic system disorders | 3 | 8.87 (2.86-27.54) |
| Sinus headache | Nervous system disorders | 8 | 8.86 (4.43-17.74) |
| Metastases to lung | Neoplasms benign, malignant and unspecified (incl cysts and polyps) | 19 | 8.81 (5.62-13.82) |
| Thrombocytosis | Blood and lymphatic system disorders | 6 | 8.79 (3.95-19.59) |
| Myocardial necrosis marker increased | Investigations | 3 | 8.65 (2.79-26.87) |
| Infusion site extravasation | General disorders and administration site conditions | 12 | 8.6 (4.88-15.16) |
| Muscle contracture | Musculoskeletal and connective tissue disorders | 3 | 8.55 (2.75-26.54) |
| Multiple-drug resistance | General disorders and administration site conditions | 5 | 8.4 (3.49-20.21) |
| Gingivitis | Infections and infestations | 8 | 8.36 (4.18-16.74) |
| Leukopenia | Blood and lymphatic system disorders | 70 | 8.23 (6.51-10.4) |
| Normochromic normocytic anaemia | Blood and lymphatic system disorders | 3 | 8.22 (2.65-25.51) |
| Pleuritic pain | Respiratory, thoracic and mediastinal disorders | 3 | 8.17 (2.63-25.37) |
| Neoplasm progression | Neoplasms benign, malignant and unspecified (incl cysts and polyps) | 64 | 8.03 (6.29-10.26) |
| Skin necrosis | Skin and subcutaneous tissue disorders | 7 | 7.97 (3.79-16.72) |
| Palmar-plantar erythrodysaesthesia syndrome | Skin and subcutaneous tissue disorders | 35 | 7.79 (5.59-10.85) |
| Aphthous ulcer | Gastrointestinal disorders | 14 | 7.66 (4.54-12.95) |
| White blood cell count decreased | Investigations | 158 | 7.66 (6.56-8.94) |
| Sputum discoloured | Respiratory, thoracic and mediastinal disorders | 15 | 7.63 (4.6-12.66) |
| Cardiac failure | Cardiac disorders | 109 | 7.61 (6.32-9.18) |
| Hepatitis B reactivation | Infections and infestations | 6 | 7.58 (3.4-16.88) |
| Neutropenia | Blood and lymphatic system disorders | 189 | 7.56 (6.56-8.71) |
| Intercepted product administration error | Injury, poisoning and procedural complications | 5 | 7.42 (3.09-17.85) |
| Iron deficiency | Metabolism and nutrition disorders | 6 | 7.4 (3.32-16.49) |
| Gastrointestinal toxicity | Gastrointestinal disorders | 6 | 7.28 (3.27-16.21) |
| Electrolyte imbalance | Metabolism and nutrition disorders | 14 | 7.12 (4.22-12.03) |
| Cytopenia | Blood and lymphatic system disorders | 17 | 7.11 (4.42-11.43) |
| Dermatomyositis | Skin and subcutaneous tissue disorders | 3 | 7.09 (2.28-22.01) |
| Premature labour | Pregnancy, puerperium and perinatal conditions | 6 | 7.06 (3.17-15.73) |
| Kounis syndrome | Cardiac disorders | 3 | 7.02 (2.26-21.8) |
| Spinal cord compression | Nervous system disorders | 5 | 6.86 (2.85-16.5) |
| Aplasia | Congenital, familial and genetic disorders | 3 | 6.86 (2.21-21.28) |
| Renal tubular disorder | Renal and urinary disorders | 3 | 6.83 (2.2-21.2) |
| Fibrin D dimer increased | Investigations | 4 | 6.83 (2.56-18.2) |
| Cachexia | Metabolism and nutrition disorders | 6 | 6.8 (3.05-15.14) |
| Haematotoxicity | Blood and lymphatic system disorders | 11 | 6.79 (3.76-12.26) |
| Laryngospasm | Respiratory, thoracic and mediastinal disorders | 3 | 6.78 (2.19-21.06) |
| Erythema nodosum | Skin and subcutaneous tissue disorders | 4 | 6.76 (2.53-18.03) |
| Disease recurrence | General disorders and administration site conditions | 59 | 6.73 (5.22-8.68) |
| Oligohydramnios | Pregnancy, puerperium and perinatal conditions | 4 | 6.67 (2.5-17.78) |
| Granulocytopenia | Blood and lymphatic system disorders | 5 | 6.67 (2.77-16.03) |
| Oral pain | Gastrointestinal disorders | 29 | 6.66 (4.63-9.58) |
| Atypical pneumonia | Infections and infestations | 4 | 6.54 (2.45-17.45) |
| Appetite disorder | Metabolism and nutrition disorders | 8 | 6.54 (3.27-13.08) |
| Radiation pneumonitis | Injury, poisoning and procedural complications | 3 | 6.49 (2.09-20.15) |
| Abdominal adhesions | Gastrointestinal disorders | 4 | 6.47 (2.43-17.26) |
| Faecaloma | Gastrointestinal disorders | 6 | 6.41 (2.88-14.29) |
| Mixed liver injury | Hepatobiliary disorders | 3 | 6.31 (2.03-19.59) |
| Metastases to peritoneum | Neoplasms benign, malignant and unspecified (incl cysts and polyps) | 3 | 6.27 (2.02-19.45) |
| Bundle branch block left | Cardiac disorders | 4 | 6.26 (2.35-16.69) |
| Oliguria | Renal and urinary disorders | 6 | 6.16 (2.77-13.72) |
| Transaminases increased | Investigations | 26 | 6.15 (4.19-9.04) |
| Sudden death | General disorders and administration site conditions | 9 | 6.1 (3.17-11.73) |
| Retinopathy | Eye disorders | 4 | 6.1 (2.29-16.26) |
| Metastases to liver | Neoplasms benign, malignant and unspecified (incl cysts and polyps) | 20 | 6.08 (3.92-9.42) |
| Pulmonary function test decreased | Investigations | 6 | 6.02 (2.7-13.41) |
| Lymphopenia | Blood and lymphatic system disorders | 16 | 5.97 (3.66-9.74) |
| Paranasal sinus discomfort | Respiratory, thoracic and mediastinal disorders | 5 | 5.89 (2.45-14.16) |
| Herpes simplex | Infections and infestations | 5 | 5.86 (2.44-14.09) |
| Foetal growth restriction | Pregnancy, puerperium and perinatal conditions | 7 | 5.76 (2.75-12.09) |
| Blood pressure diastolic increased | Investigations | 5 | 5.64 (2.35-13.56) |
| Vein disorder | Vascular disorders | 5 | 5.58 (2.32-13.41) |
| Nail disorder | Skin and subcutaneous tissue disorders | 8 | 5.56 (2.78-11.11) |
| Neutrophilia | Blood and lymphatic system disorders | 6 | 5.48 (2.46-12.21) |
| Interstitial lung disease | Respiratory, thoracic and mediastinal disorders | 46 | 5.47 (4.1-7.3) |
| Cystitis haemorrhagic | Renal and urinary disorders | 4 | 5.46 (2.05-14.57) |
| Premature baby | Pregnancy, puerperium and perinatal conditions | 32 | 5.41 (3.83-7.65) |
| Gamma-glutamyltransferase increased | Investigations | 17 | 5.33 (3.31-8.58) |
| Foetal death | Pregnancy, puerperium and perinatal conditions | 6 | 5.29 (2.37-11.77) |
| Hepatic failure | Hepatobiliary disorders | 25 | 5.21 (3.52-7.71) |
| Pneumocystis jirovecii pneumonia | Infections and infestations | 11 | 5.12 (2.83-9.24) |
| Pancreatitis acute | Gastrointestinal disorders | 18 | 5.1 (3.21-8.1) |
| Tumour marker increased | Investigations | 5 | 5.07 (2.11-12.2) |
| Energy increased | General disorders and administration site conditions | 6 | 5.03 (2.26-11.21) |
| Troponin increased | Investigations | 6 | 5.03 (2.26-11.2) |
| Incontinence | Renal and urinary disorders | 9 | 4.89 (2.54-9.39) |
| Gastroenteritis | Infections and infestations | 12 | 4.87 (2.77-8.58) |
| Septic shock | Infections and infestations | 35 | 4.69 (3.37-6.53) |
| Hepatocellular injury | Hepatobiliary disorders | 16 | 4.59 (2.81-7.49) |
| Neurotoxicity | Nervous system disorders | 15 | 4.56 (2.75-7.56) |
| Aspartate aminotransferase increased | Investigations | 33 | 4.55 (3.24-6.4) |
| Alanine aminotransferase increased | Investigations | 39 | 4.41 (3.22-6.03) |
| Deafness | Ear and labyrinth disorders | 21 | 4.36 (2.84-6.69) |
| Disease progression | General disorders and administration site conditions | 87 | 4.19 (3.4-5.17) |
| Pancytopenia | Blood and lymphatic system disorders | 38 | 4.18 (3.04-5.74) |
| Oral candidiasis | Infections and infestations | 9 | 4.14 (2.16-7.97) |
| Metastases to central nervous system | Neoplasms benign, malignant and unspecified (incl cysts and polyps) | 9 | 3.95 (2.05-7.59) |
| Neuropathy peripheral | Nervous system disorders | 71 | 3.88 (3.07-4.89) |
| Mouth ulceration | Gastrointestinal disorders | 14 | 3.86 (2.29-6.52) |
| Hypokalaemia | Metabolism and nutrition disorders | 30 | 3.83 (2.68-5.47) |
| Pleural effusion | Respiratory, thoracic and mediastinal disorders | 40 | 3.78 (2.77-5.15) |
| Cardiovascular disorder | Cardiac disorders | 10 | 3.78 (2.03-7.02) |
| Thrombocytopenia | Blood and lymphatic system disorders | 68 | 3.56 (2.81-4.52) |
| Pyrexia | General disorders and administration site conditions | 217 | 3.55 (3.11-4.05) |
| Full blood count decreased | Investigations | 15 | 3.49 (2.1-5.79) |
| Hyponatraemia | Metabolism and nutrition disorders | 33 | 3.34 (2.38-4.7) |
| Asthenia | General disorders and administration site conditions | 222 | 3.24 (2.85-3.69) |
| Vomiting | Gastrointestinal disorders | 251 | 3.08 (2.72-3.48) |
| Hepatic enzyme increased | Investigations | 34 | 2.94 (2.1-4.12) |
| Anaemia | Blood and lymphatic system disorders | 98 | 2.9 (2.38-3.53) |
