# Supplementary material for: Epirubicin and gait apraxia: a real-world data analysis of the FDA Adverse Event Reporting System database
Source: Front Pharmacol. 2023 Sep 14;14:1249845. doi: 10.3389/fphar.2023.1249845 (PMC10536159; doi:10.3389/fphar.2023.1249845)
Supplement: Supplementary file 5 [file Table4.docx]

Supplementary Table S4 The comprehensive detailed information of all AEs at the PTs level identified by BCPNN algorithms.

| Preferred Terms | SOC | Case Number | BCPNN (95% two side CI) |
| --- | --- | --- | --- |
| Nausea | Gastrointestinal disorders | 300 | 1.05 (0.88-1.22) |
| Fatigue | General disorders and administration site conditions | 184 | 0.27 (0.06-0.48) |
| Diarrhoea | Gastrointestinal disorders | 162 | 0.38 (0.15-0.61) |
| Hepatic artery stenosis | Hepatobiliary disorders | 8 | 8.87 (2.1-4.19) |
| Endocardial fibrosis | Cardiac disorders | 3 | 8.74 (0.42-3.56) |
| Gait apraxia | Nervous system disorders | 3 | 8.74 (0.42-3.56) |
| Alopecia | Skin and subcutaneous tissue disorders | 98 | 1.16 (0.85-1.43) |
| Cardiac perfusion defect | Cardiac disorders | 3 | 8.37 (0.44-3.53) |
| Decreased appetite | Metabolism and nutrition disorders | 90 | 1.02 (0.7-1.31) |
| Constipation | Gastrointestinal disorders | 76 | 0.92 (0.58-1.24) |
| Hepatic artery occlusion | Hepatobiliary disorders | 3 | 8.27 (0.45-3.52) |
| Administration site oedema | General disorders and administration site conditions | 4 | 8.15 (0.94-3.66) |
| Paraesthesia | Nervous system disorders | 62 | 1.1 (0.71-1.44) |
| Post embolisation syndrome | Injury, poisoning and procedural complications | 4 | 7.61 (0.95-3.63) |
| Abdominal pain | Gastrointestinal disorders | 58 | 0.5 (0.11-0.86) |
| Chest pain | General disorders and administration site conditions | 51 | 0.73 (0.31-1.12) |
| General physical health deterioration | General disorders and administration site conditions | 49 | 1.33 (0.88-1.69) |
| Myalgia | Musculoskeletal and connective tissue disorders | 47 | 0.65 (0.21-1.05) |
| Malignant neoplasm progression | Neoplasms benign, malignant and unspecified (incl cysts and polyps) | 47 | 1.18 (0.73-1.56) |
| Menopausal disorder | Reproductive system and breast disorders | 3 | 7.15 (0.48-3.46) |
| Maternal exposure during pregnancy | Injury, poisoning and procedural complications | 42 | 1.26 (0.78-1.66) |
| Gastrointestinal disorder | Gastrointestinal disorders | 38 | 1.37 (0.85-1.77) |
| Granulocyte count decreased | Investigations | 26 | 7.11 (3.94-5.07) |
| Chest discomfort | General disorders and administration site conditions | 37 | 1.03 (0.52-1.46) |
| Oropharyngeal pain | Respiratory, thoracic and mediastinal disorders | 37 | 1.03 (0.52-1.46) |
| Intestinal atresia | Congenital, familial and genetic disorders | 3 | 7 (0.48-3.45) |
| Breast cancer | Neoplasms benign, malignant and unspecified (incl cysts and polyps) | 35 | 0.7 (0.19-1.16) |
| Biliary fistula | Hepatobiliary disorders | 3 | 6.9 (0.48-3.44) |
| Chills | General disorders and administration site conditions | 35 | 0.74 (0.23-1.2) |
| Sepsis | Infections and infestations | 34 | 0.76 (0.25-1.22) |
| Acute cutaneous lupus erythematosus | Skin and subcutaneous tissue disorders | 4 | 6.79 (0.95-3.59) |
| Palpitations | Cardiac disorders | 33 | 0.67 (0.15-1.14) |
| Dysphagia | Gastrointestinal disorders | 32 | 0.95 (0.4-1.41) |
| Dysgeusia | Nervous system disorders | 31 | 1.21 (0.64-1.66) |
| Platelet count decreased | Investigations | 30 | 0.61 (0.07-1.11) |
| Respiratory failure | Respiratory, thoracic and mediastinal disorders | 30 | 1.29 (0.7-1.74) |
| Miller Fisher syndrome | Nervous system disorders | 4 | 6.67 (0.95-3.58) |
| Right atrial dilatation | Cardiac disorders | 7 | 6.61 (1.86-3.94) |
| Pulmonary embolism | Respiratory, thoracic and mediastinal disorders | 29 | 0.96 (0.38-1.44) |
| Abdominal distension | Gastrointestinal disorders | 28 | 0.58 (0.02-1.1) |
| Hot flush | Vascular disorders | 26 | 0.95 (0.35-1.46) |
| Tachycardia | Cardiac disorders | 25 | 0.73 (0.13-1.26) |
| Productive cough | Respiratory, thoracic and mediastinal disorders | 23 | 1.32 (0.64-1.82) |
| Oedema | General disorders and administration site conditions | 23 | 1.33 (0.65-1.83) |
| Disturbance in attention | Nervous system disorders | 22 | 1.18 (0.5-1.7) |
| Discomfort | General disorders and administration site conditions | 22 | 0.95 (0.29-1.5) |
| Deep vein thrombosis | Vascular disorders | 22 | 1.12 (0.44-1.65) |
| Cognitive disorder | Nervous system disorders | 22 | 1.35 (0.65-1.86) |
| Stomatitis | Gastrointestinal disorders | 21 | 0.86 (0.19-1.42) |
| Administration site extravasation | General disorders and administration site conditions | 19 | 6.43 (3.38-4.69) |
| Xerophthalmia | Eye disorders | 8 | 6.38 (2.06-4.01) |
| Cardio-respiratory arrest | Cardiac disorders | 18 | 1.44 (0.65-1.98) |
| Dyspnoea exertional | Respiratory, thoracic and mediastinal disorders | 18 | 1.31 (0.54-1.87) |
| Inflammation | General disorders and administration site conditions | 17 | 0.87 (0.12-1.48) |
| Placental disorder | Pregnancy, puerperium and perinatal conditions | 10 | 6.25 (2.4-4.16) |
| Colitis | Gastrointestinal disorders | 17 | 1.32 (0.52-1.88) |
| Neutrophil percentage decreased | Investigations | 4 | 6.13 (0.93-3.55) |
| Haematuria | Renal and urinary disorders | 16 | 1.27 (0.46-1.86) |
| Metabolic acidosis | Metabolism and nutrition disorders | 16 | 1.51 (0.66-2.06) |
| Cardiac dysfunction | Cardiac disorders | 49 | 6.1 (4.45-5.28) |
| Lower respiratory tract infection | Infections and infestations | 15 | 0.8 (0.02-1.46) |
| Bladder irritation | Renal and urinary disorders | 8 | 5.97 (2.02-3.97) |
| Refractory cancer | Neoplasms benign, malignant and unspecified (incl cysts and polyps) | 3 | 5.97 (0.47-3.39) |
| Blood bilirubin increased | Investigations | 14 | 1.75 (0.79-2.28) |
| Metastases to thorax | Neoplasms benign, malignant and unspecified (incl cysts and polyps) | 3 | 5.89 (0.47-3.39) |
| Hepatotoxicity | Hepatobiliary disorders | 13 | 1.67 (0.69-2.23) |
| Hypophagia | Metabolism and nutrition disorders | 13 | 1.47 (0.52-2.07) |
| Ascites | Gastrointestinal disorders | 13 | 1.32 (0.4-1.94) |
| Glossodynia | Gastrointestinal disorders | 12 | 1.77 (0.72-2.33) |
| C-reactive protein increased | Investigations | 12 | 0.94 (0.04-1.64) |
| Ear pain | Ear and labyrinth disorders | 12 | 1.77 (0.72-2.32) |
| Liver function test abnormal | Investigations | 11 | 1.48 (0.45-2.11) |
| Clostridium difficile infection | Infections and infestations | 11 | 1.2 (0.22-1.89) |
| Acute respiratory failure | Respiratory, thoracic and mediastinal disorders | 11 | 1.63 (0.56-2.23) |
| Red blood cell count decreased | Investigations | 11 | 1.06 (0.1-1.77) |
| Acute respiratory distress syndrome | Respiratory, thoracic and mediastinal disorders | 10 | 1.8 (0.64-2.38) |
| Presyncope | Nervous system disorders | 10 | 1.15 (0.13-1.87) |
| Sensory disturbance | Nervous system disorders | 10 | 1.75 (0.6-2.34) |
| Pseudocirrhosis | Hepatobiliary disorders | 4 | 5.74 (0.91-3.52) |
| Merycism | Psychiatric disorders | 3 | 5.73 (0.46-3.38) |
| Gastritis | Gastrointestinal disorders | 10 | 1.2 (0.17-1.91) |
| Impaired work ability | Social circumstances | 10 | 1.35 (0.29-2.03) |
| Dilated cardiomyopathy | Cardiac disorders | 43 | 5.67 (4.13-5.01) |
| Pulmonary hypertension | Respiratory, thoracic and mediastinal disorders | 9 | 1.29 (0.19-2.01) |
| Pulmonary fibrosis | Respiratory, thoracic and mediastinal disorders | 9 | 1.58 (0.4-2.23) |
| Increased appetite | Metabolism and nutrition disorders | 9 | 1.61 (0.43-2.25) |
| Catheter site related reaction | General disorders and administration site conditions | 5 | 5.63 (1.26-3.64) |
| Hepatic steatosis | Hepatobiliary disorders | 9 | 1.63 (0.44-2.27) |
| Photophobia | Eye disorders | 9 | 1.47 (0.32-2.15) |
| Circulatory collapse | Vascular disorders | 9 | 1.71 (0.5-2.33) |
| Pericardial effusion | Cardiac disorders | 9 | 1.17 (0.09-1.92) |
| Metastases to the mediastinum | Neoplasms benign, malignant and unspecified (incl cysts and polyps) | 4 | 5.61 (0.9-3.51) |
| Jaundice | Hepatobiliary disorders | 9 | 1.11 (0.04-1.87) |
| Subclavian vein thrombosis | Vascular disorders | 8 | 5.47 (1.96-3.9) |
| Carbohydrate antigen 15-3 increased | Investigations | 6 | 5.43 (1.52-3.72) |
| Cardiogenic shock | Cardiac disorders | 8 | 1.78 (0.47-2.4) |
| Joint range of motion decreased | Musculoskeletal and connective tissue disorders | 8 | 1.82 (0.5-2.42) |
| Psychotic behaviour | Psychiatric disorders | 7 | 5.41 (1.75-3.81) |
| Neutropenic sepsis | Infections and infestations | 55 | 5.4 (4.21-4.99) |
| Breast cancer recurrent | Neoplasms benign, malignant and unspecified (incl cysts and polyps) | 22 | 5.39 (3.31-4.52) |
| Myocardial oedema | Cardiac disorders | 3 | 5.39 (0.44-3.36) |
| Soft tissue sarcoma | Neoplasms benign, malignant and unspecified (incl cysts and polyps) | 4 | 5.38 (0.89-3.49) |
| Quality of life decreased | Investigations | 7 | 1.65 (0.29-2.33) |
| Muscle atrophy | Musculoskeletal and connective tissue disorders | 7 | 1.84 (0.42-2.46) |
| Toxic skin eruption | Skin and subcutaneous tissue disorders | 7 | 2.07 (0.56-2.6) |
| Paraesthesia oral | Gastrointestinal disorders | 7 | 1.47 (0.16-2.2) |
| Pericarditis | Cardiac disorders | 7 | 1.58 (0.24-2.28) |
| Allodynia | Nervous system disorders | 5 | 5.24 (1.22-3.59) |
| Acute hepatic failure | Hepatobiliary disorders | 7 | 1.54 (0.21-2.25) |
| Myelodysplastic syndrome | Neoplasms benign, malignant and unspecified (incl cysts and polyps) | 7 | 1.54 (0.21-2.25) |
| Soft tissue necrosis | Musculoskeletal and connective tissue disorders | 4 | 5.19 (0.87-3.47) |
| Blood magnesium decreased | Investigations | 6 | 1.89 (0.32-2.51) |
| Appendicitis | Infections and infestations | 6 | 1.9 (0.34-2.52) |
| Supraventricular tachycardia | Cardiac disorders | 6 | 2 (0.39-2.58) |
| Myocardial fibrosis | Cardiac disorders | 5 | 5.14 (1.21-3.58) |
| Aortic dilatation | Vascular disorders | 6 | 5.12 (1.48-3.68) |
| Hypercreatininaemia | Metabolism and nutrition disorders | 3 | 5.12 (0.43-3.33) |
| Acute leukaemia | Neoplasms benign, malignant and unspecified (incl cysts and polyps) | 8 | 5.07 (1.89-3.83) |
| Inappropriate antidiuretic hormone secretion | Endocrine disorders | 6 | 1.83 (0.29-2.47) |
| Temperature intolerance | General disorders and administration site conditions | 6 | 1.68 (0.19-2.38) |
| Myocardial ischaemia | Cardiac disorders | 6 | 1.9 (0.33-2.52) |
| Amnestic disorder | Nervous system disorders | 3 | 5.05 (0.42-3.33) |
| Arterial thrombosis | Vascular disorders | 10 | 5.02 (2.2-3.95) |
| Myelosuppression | Blood and lymphatic system disorders | 121 | 4.99 (4.4-4.93) |
| Tongue coated | Gastrointestinal disorders | 8 | 4.99 (1.88-3.81) |
| Hyperbilirubinaemia | Hepatobiliary disorders | 6 | 1.71 (0.21-2.39) |
| Hyperamylasaemia | Metabolism and nutrition disorders | 3 | 4.98 (0.42-3.32) |
| Cardiotoxicity | Cardiac disorders | 53 | 4.98 (3.93-4.73) |
| Flank pain | Musculoskeletal and connective tissue disorders | 5 | 1.6 (0-2.36) |
| Aortitis | Vascular disorders | 4 | 4.92 (0.84-3.44) |
| Tumour lysis syndrome | Metabolism and nutrition disorders | 5 | 1.64 (0.02-2.38) |
| Hyperlipasaemia | Metabolism and nutrition disorders | 3 | 4.83 (0.4-3.31) |
| Aortic thrombosis | Vascular disorders | 5 | 4.79 (1.16-3.53) |
| Oesophagitis | Gastrointestinal disorders | 5 | 1.62 (0.01-2.37) |
| Abdominal sepsis | Infections and infestations | 4 | 4.77 (0.83-3.42) |
| Infusion site erythema | General disorders and administration site conditions | 5 | 1.7 (0.06-2.42) |
| Hepatic function abnormal | Hepatobiliary disorders | 161 | 4.75 (4.31-4.77) |
| Papule | Skin and subcutaneous tissue disorders | 5 | 1.93 (0.19-2.55) |
| Initial insomnia | Psychiatric disorders | 5 | 1.72 (0.07-2.43) |
| Acute coronary syndrome | Cardiac disorders | 5 | 1.81 (0.13-2.49) |
| Anastomotic leak | Injury, poisoning and procedural complications | 3 | 4.74 (0.39-3.3) |
| Iatrogenic injury | Injury, poisoning and procedural complications | 3 | 4.72 (0.39-3.3) |
| Dilatation ventricular | Cardiac disorders | 5 | 4.69 (1.15-3.51) |
| Febrile bone marrow aplasia | Blood and lymphatic system disorders | 20 | 4.68 (2.93-4.19) |
| Subacute cutaneous lupus erythematosus | Skin and subcutaneous tissue disorders | 10 | 4.62 (2.09-3.84) |
| Coronary artery thrombosis | Cardiac disorders | 6 | 4.62 (1.4-3.59) |
| Bone marrow failure | Blood and lymphatic system disorders | 105 | 4.62 (4.05-4.61) |
| Troponin T increased | Investigations | 5 | 4.56 (1.12-3.49) |
| Hyperammonaemia | Metabolism and nutrition disorders | 4 | 2.21 (0.13-2.72) |
| Hepatitis acute | Hepatobiliary disorders | 4 | 1.95 (0.01-2.59) |
| Neutropenic infection | Infections and infestations | 3 | 4.52 (0.37-3.27) |
| Rash vesicular | Skin and subcutaneous tissue disorders | 4 | 2.29 (0.17-2.75) |
| Troponin I increased | Investigations | 6 | 4.49 (1.37-3.56) |
| Hydrothorax | Respiratory, thoracic and mediastinal disorders | 4 | 4.48 (0.79-3.38) |
| Small for dates baby | Pregnancy, puerperium and perinatal conditions | 4 | 2.18 (0.12-2.7) |
| Extravasation | General disorders and administration site conditions | 16 | 4.48 (2.6-4.01) |
| Second primary malignancy | Neoplasms benign, malignant and unspecified (incl cysts and polyps) | 43 | 4.45 (3.45-4.33) |
| Ventricular hypokinesia | Cardiac disorders | 11 | 4.45 (2.16-3.83) |
| Klebsiella infection | Infections and infestations | 4 | 2.18 (0.12-2.7) |
| N-terminal prohormone brain natriuretic peptide increased | Investigations | 6 | 4.41 (1.35-3.54) |
| Agranulocytosis | Blood and lymphatic system disorders | 63 | 4.38 (3.63-4.36) |
| Cholecystitis acute | Hepatobiliary disorders | 4 | 2.31 (0.18-2.76) |
| Cardiac death | General disorders and administration site conditions | 4 | 4.38 (0.77-3.36) |
| Ventricular dysfunction | Cardiac disorders | 5 | 4.37 (1.09-3.45) |
| Anal ulcer | Gastrointestinal disorders | 3 | 4.35 (0.35-3.25) |
| Bile duct stenosis | Hepatobiliary disorders | 4 | 4.33 (0.76-3.36) |
| Acute promyelocytic leukaemia | Neoplasms benign, malignant and unspecified (incl cysts and polyps) | 3 | 4.31 (0.35-3.25) |
| Cutaneous symptom | Skin and subcutaneous tissue disorders | 3 | 4.31 (0.35-3.25) |
| Hypertransaminasaemia | Hepatobiliary disorders | 21 | 4.3 (2.79-4.03) |
| Hepatitis B | Infections and infestations | 19 | 4.25 (2.68-3.97) |
| Menopause | Social circumstances | 8 | 4.24 (1.7-3.63) |
| Ocular toxicity | Eye disorders | 3 | 4.24 (0.34-3.24) |
| Neutropenic colitis | Gastrointestinal disorders | 7 | 4.21 (1.51-3.56) |
| Atypical haemolytic uraemic syndrome | Blood and lymphatic system disorders | 3 | 4.21 (0.33-3.23) |
| Infusion site discolouration | General disorders and administration site conditions | 3 | 4.2 (0.33-3.23) |
| Jugular vein thrombosis | Vascular disorders | 5 | 4.19 (1.05-3.42) |
| Kidney enlargement | Renal and urinary disorders | 3 | 4.12 (0.32-3.22) |
| Left ventricular dysfunction | Cardiac disorders | 20 | 4.09 (2.64-3.91) |
| Hepatic lesion | Hepatobiliary disorders | 13 | 4.09 (2.21-3.76) |
| Liver injury | Hepatobiliary disorders | 61 | 4.04 (3.35-4.09) |
| Mental fatigue | Psychiatric disorders | 5 | 4.03 (1.02-3.38) |
| Axillary mass | Musculoskeletal and connective tissue disorders | 3 | 3.97 (0.3-3.19) |
| Respiratory alkalosis | Respiratory, thoracic and mediastinal disorders | 4 | 3.96 (0.7-3.29) |
| Lymphangiosis carcinomatosa | Neoplasms benign, malignant and unspecified (incl cysts and polyps) | 3 | 3.95 (0.3-3.19) |
| Biliary tract disorder | Hepatobiliary disorders | 3 | 3.92 (0.29-3.19) |
| Acute pulmonary oedema | Respiratory, thoracic and mediastinal disorders | 13 | 3.92 (2.14-3.68) |
| Dysentery | Infections and infestations | 6 | 3.89 (1.22-3.41) |
| Pancreatitis necrotising | Gastrointestinal disorders | 5 | 3.89 (0.98-3.35) |
| Diastolic dysfunction | Cardiac disorders | 7 | 3.88 (1.42-3.46) |
| Right ventricular dysfunction | Cardiac disorders | 3 | 3.86 (0.28-3.18) |
| Acute lymphocytic leukaemia | Neoplasms benign, malignant and unspecified (incl cysts and polyps) | 8 | 3.86 (1.57-3.5) |
| Metastases to lymph nodes | Neoplasms benign, malignant and unspecified (incl cysts and polyps) | 17 | 3.84 (2.36-3.72) |
| Adenocarcinoma gastric | Neoplasms benign, malignant and unspecified (incl cysts and polyps) | 3 | 3.82 (0.27-3.17) |
| Catheter site pain | General disorders and administration site conditions | 7 | 3.79 (1.39-3.43) |
| Cardiomyopathy | Cardiac disorders | 32 | 3.78 (2.81-3.82) |
| Metastasis | Neoplasms benign, malignant and unspecified (incl cysts and polyps) | 17 | 3.77 (2.32-3.68) |
| Biliary dilatation | Hepatobiliary disorders | 3 | 3.76 (0.26-3.16) |
| Dislocation of vertebra | Injury, poisoning and procedural complications | 3 | 3.75 (0.26-3.16) |
| Fanconi syndrome acquired | Renal and urinary disorders | 5 | 3.72 (0.94-3.3) |
| Hyperammonaemic encephalopathy | Nervous system disorders | 5 | 3.71 (0.94-3.3) |
| Menopausal symptoms | Reproductive system and breast disorders | 5 | 3.7 (0.93-3.3) |
| Ejection fraction decreased | Investigations | 36 | 3.69 (2.81-3.77) |
| Pharyngeal erythema | Respiratory, thoracic and mediastinal disorders | 5 | 3.69 (0.93-3.29) |
| Angiopathy | Vascular disorders | 11 | 3.69 (1.86-3.53) |
| Drug-induced liver injury | Hepatobiliary disorders | 75 | 3.68 (3.13-3.8) |
| Phlebitis | Vascular disorders | 10 | 3.68 (1.75-3.5) |
| Breast cancer metastatic | Neoplasms benign, malignant and unspecified (incl cysts and polyps) | 20 | 3.67 (2.4-3.66) |
| Skin hypopigmentation | Skin and subcutaneous tissue disorders | 5 | 3.66 (0.92-3.28) |
| Myocardial injury | Cardiac disorders | 4 | 3.64 (0.63-3.21) |
| Appendicitis perforated | Infections and infestations | 5 | 3.63 (0.91-3.28) |
| Skin sensitisation | Skin and subcutaneous tissue disorders | 3 | 3.62 (0.24-3.13) |
| Vena cava thrombosis | Vascular disorders | 3 | 3.61 (0.24-3.13) |
| Cardiac failure chronic | Cardiac disorders | 11 | 3.61 (1.82-3.49) |
| Adenocarcinoma | Neoplasms benign, malignant and unspecified (incl cysts and polyps) | 5 | 3.6 (0.91-3.27) |
| Hyperpyrexia | General disorders and administration site conditions | 8 | 3.59 (1.47-3.4) |
| Neutrophil count decreased | Investigations | 86 | 3.58 (3.1-3.72) |
| Multi-organ disorder | General disorders and administration site conditions | 3 | 3.57 (0.23-3.12) |
| Mucosal inflammation | General disorders and administration site conditions | 54 | 3.56 (2.91-3.69) |
| Body temperature abnormal | Investigations | 5 | 3.54 (0.89-3.25) |
| Odynophagia | Gastrointestinal disorders | 11 | 3.52 (1.78-3.45) |
| Mucosal dryness | General disorders and administration site conditions | 4 | 3.5 (0.59-3.18) |
| Catheter site erythema | General disorders and administration site conditions | 5 | 3.49 (0.87-3.23) |
| Metastases to bone | Neoplasms benign, malignant and unspecified (incl cysts and polyps) | 33 | 3.48 (2.61-3.6) |
| Mucosal disorder | General disorders and administration site conditions | 4 | 3.46 (0.58-3.17) |
| Acute myeloid leukaemia | Neoplasms benign, malignant and unspecified (incl cysts and polyps) | 29 | 3.46 (2.51-3.57) |
| Tetany | Metabolism and nutrition disorders | 3 | 3.44 (0.2-3.09) |
| Peripheral sensory neuropathy | Nervous system disorders | 11 | 3.42 (1.73-3.4) |
| Pseudomonal sepsis | Infections and infestations | 3 | 3.42 (0.2-3.09) |
| Maternal exposure timing unspecified | Injury, poisoning and procedural complications | 5 | 3.42 (0.85-3.21) |
| Cardiac failure acute | Cardiac disorders | 13 | 3.41 (1.88-3.43) |
| Nail infection | Infections and infestations | 3 | 3.4 (0.19-3.08) |
| Febrile neutropenia | Blood and lymphatic system disorders | 122 | 3.38 (3.02-3.54) |
| Neoplasm recurrence | Neoplasms benign, malignant and unspecified (incl cysts and polyps) | 6 | 3.38 (1.06-3.24) |
| Liver abscess | Infections and infestations | 6 | 3.37 (1.05-3.24) |
| Metastases to meninges | Neoplasms benign, malignant and unspecified (incl cysts and polyps) | 4 | 3.34 (0.55-3.13) |
| Malignant pleural effusion | Neoplasms benign, malignant and unspecified (incl cysts and polyps) | 3 | 3.33 (0.18-3.07) |
| Livedo reticularis | Skin and subcutaneous tissue disorders | 3 | 3.31 (0.17-3.06) |
| Mitral valve incompetence | Cardiac disorders | 14 | 3.3 (1.88-3.38) |
| Injection site hypersensitivity | General disorders and administration site conditions | 5 | 3.27 (0.8-3.16) |
| Intervertebral discitis | Infections and infestations | 3 | 3.27 (0.16-3.05) |
| Periodontitis | Infections and infestations | 3 | 3.27 (0.16-3.05) |
| Tricuspid valve incompetence | Cardiac disorders | 9 | 3.26 (1.45-3.28) |
| Polyneuropathy | Nervous system disorders | 21 | 3.26 (2.17-3.4) |
| Radiotherapy | Surgical and medical procedures | 3 | 3.24 (0.15-3.05) |
| Skin toxicity | Skin and subcutaneous tissue disorders | 9 | 3.22 (1.43-3.26) |
| Portal vein thrombosis | Hepatobiliary disorders | 5 | 3.21 (0.78-3.14) |
| Cerebral venous thrombosis | Nervous system disorders | 3 | 3.18 (0.14-3.03) |
| Venous thrombosis | Vascular disorders | 6 | 3.17 (0.98-3.16) |
| Lichenoid keratosis | Skin and subcutaneous tissue disorders | 3 | 3.16 (0.14-3.03) |
| Jaundice neonatal | Pregnancy, puerperium and perinatal conditions | 3 | 3.16 (0.14-3.03) |
| Gastrointestinal necrosis | Gastrointestinal disorders | 4 | 3.15 (0.49-3.08) |
| Lymphadenopathy mediastinal | Blood and lymphatic system disorders | 3 | 3.14 (0.13-3.02) |
| Sinus headache | Nervous system disorders | 8 | 3.14 (1.28-3.2) |
| Metastases to lung | Neoplasms benign, malignant and unspecified (incl cysts and polyps) | 19 | 3.14 (2.01-3.31) |
| Thrombocytosis | Blood and lymphatic system disorders | 6 | 3.13 (0.96-3.15) |
| Myocardial necrosis marker increased | Investigations | 3 | 3.11 (0.12-3.01) |
| Infusion site extravasation | General disorders and administration site conditions | 12 | 3.1 (1.64-3.24) |
| Muscle contracture | Musculoskeletal and connective tissue disorders | 3 | 3.09 (0.12-3.01) |
| Multiple-drug resistance | General disorders and administration site conditions | 5 | 3.07 (0.73-3.09) |
| Gingivitis | Infections and infestations | 8 | 3.06 (1.24-3.16) |
| Leukopenia | Blood and lymphatic system disorders | 70 | 3.04 (2.55-3.24) |
| Normochromic normocytic anaemia | Blood and lymphatic system disorders | 3 | 3.04 (0.1-3) |
| Pleuritic pain | Respiratory, thoracic and mediastinal disorders | 3 | 3.03 (0.1-2.99) |
| Neoplasm progression | Neoplasms benign, malignant and unspecified (incl cysts and polyps) | 64 | 3 (2.49-3.21) |
| Skin necrosis | Skin and subcutaneous tissue disorders | 7 | 2.99 (1.07-3.11) |
| Palmar-plantar erythrodysaesthesia syndrome | Skin and subcutaneous tissue disorders | 35 | 2.96 (2.23-3.19) |
| Aphthous ulcer | Gastrointestinal disorders | 14 | 2.93 (1.66-3.15) |
| White blood cell count decreased | Investigations | 158 | 2.93 (2.64-3.11) |
| Sputum discoloured | Respiratory, thoracic and mediastinal disorders | 15 | 2.93 (1.71-3.15) |
| Cardiac failure | Cardiac disorders | 109 | 2.93 (2.56-3.12) |
| Hepatitis B reactivation | Infections and infestations | 6 | 2.92 (0.87-3.06) |
| Neutropenia | Blood and lymphatic system disorders | 189 | 2.91 (2.65-3.08) |
| Intercepted product administration error | Injury, poisoning and procedural complications | 5 | 2.89 (0.66-3.02) |
| Iron deficiency | Metabolism and nutrition disorders | 6 | 2.88 (0.86-3.04) |
| Gastrointestinal toxicity | Gastrointestinal disorders | 6 | 2.86 (0.85-3.03) |
| Electrolyte imbalance | Metabolism and nutrition disorders | 14 | 2.83 (1.59-3.08) |
| Cytopenia | Blood and lymphatic system disorders | 17 | 2.83 (1.72-3.09) |
| Dermatomyositis | Skin and subcutaneous tissue disorders | 3 | 2.82 (0.04-2.93) |
| Premature labour | Pregnancy, puerperium and perinatal conditions | 6 | 2.82 (0.83-3.01) |
| Kounis syndrome | Cardiac disorders | 3 | 2.81 (0.04-2.93) |
| Spinal cord compression | Nervous system disorders | 5 | 2.78 (0.61-2.97) |
| Aplasia | Congenital, familial and genetic disorders | 3 | 2.77 (0.03-2.92) |
| Renal tubular disorder | Renal and urinary disorders | 3 | 2.77 (0.03-2.92) |
| Fibrin D dimer increased | Investigations | 4 | 2.77 (0.36-2.95) |
| Cachexia | Metabolism and nutrition disorders | 6 | 2.76 (0.8-2.99) |
| Haematotoxicity | Blood and lymphatic system disorders | 11 | 2.76 (1.36-3.03) |
| Laryngospasm | Respiratory, thoracic and mediastinal disorders | 3 | 2.76 (0.03-2.92) |
| Erythema nodosum | Skin and subcutaneous tissue disorders | 4 | 2.75 (0.36-2.94) |
| Disease recurrence | General disorders and administration site conditions | 59 | 2.75 (2.24-2.99) |
| Oligohydramnios | Pregnancy, puerperium and perinatal conditions | 4 | 2.73 (0.35-2.94) |
| Granulocytopenia | Blood and lymphatic system disorders | 5 | 2.73 (0.6-2.96) |
| Oral pain | Gastrointestinal disorders | 29 | 2.73 (1.96-3.01) |
| Atypical pneumonia | Infections and infestations | 4 | 2.71 (0.34-2.92) |
| Appetite disorder | Metabolism and nutrition disorders | 8 | 2.71 (1.05-2.98) |
| Radiation pneumonitis | Injury, poisoning and procedural complications | 3 | 2.7 (0.01-2.9) |
| Abdominal adhesions | Gastrointestinal disorders | 4 | 2.69 (0.33-2.92) |
| Faecaloma | Gastrointestinal disorders | 6 | 2.68 (0.76-2.95) |
| Bundle branch block left | Cardiac disorders | 4 | 2.64 (0.32-2.9) |
| Oliguria | Renal and urinary disorders | 6 | 2.62 (0.73-2.92) |
| Transaminases increased | Investigations | 26 | 2.62 (1.81-2.92) |
| Sudden death | General disorders and administration site conditions | 9 | 2.61 (1.1-2.93) |
| Retinopathy | Eye disorders | 4 | 2.61 (0.3-2.89) |
| Metastases to liver | Neoplasms benign, malignant and unspecified (incl cysts and polyps) | 20 | 2.6 (1.66-2.92) |
| Pulmonary function test decreased | Investigations | 6 | 2.59 (0.72-2.9) |
| Lymphopenia | Blood and lymphatic system disorders | 16 | 2.58 (1.5-2.91) |
| Paranasal sinus discomfort | Respiratory, thoracic and mediastinal disorders | 5 | 2.56 (0.52-2.88) |
| Herpes simplex | Infections and infestations | 5 | 2.55 (0.51-2.87) |
| Foetal growth restriction | Pregnancy, puerperium and perinatal conditions | 7 | 2.52 (0.83-2.87) |
| Blood pressure diastolic increased | Investigations | 5 | 2.49 (0.49-2.85) |
| Vein disorder | Vascular disorders | 5 | 2.48 (0.48-2.84) |
| Nail disorder | Skin and subcutaneous tissue disorders | 8 | 2.47 (0.92-2.84) |
| Neutrophilia | Blood and lymphatic system disorders | 6 | 2.45 (0.65-2.83) |
| Interstitial lung disease | Respiratory, thoracic and mediastinal disorders | 46 | 2.45 (1.9-2.74) |
| Cystitis haemorrhagic | Renal and urinary disorders | 4 | 2.45 (0.24-2.82) |
| Premature baby | Pregnancy, puerperium and perinatal conditions | 32 | 2.43 (1.75-2.76) |
| Gamma-glutamyltransferase increased | Investigations | 17 | 2.41 (1.42-2.78) |
| Foetal death | Pregnancy, puerperium and perinatal conditions | 6 | 2.4 (0.62-2.8) |
| Hepatic failure | Hepatobiliary disorders | 25 | 2.38 (1.6-2.73) |
| Pneumocystis jirovecii pneumonia | Infections and infestations | 11 | 2.35 (1.09-2.76) |
| Pancreatitis acute | Gastrointestinal disorders | 18 | 2.35 (1.4-2.73) |
| Tumour marker increased | Investigations | 5 | 2.34 (0.41-2.77) |
| Energy increased | General disorders and administration site conditions | 6 | 2.33 (0.58-2.77) |
| Troponin increased | Investigations | 6 | 2.33 (0.58-2.77) |
| Incontinence | Renal and urinary disorders | 9 | 2.29 (0.9-2.73) |
| Gastroenteritis | Infections and infestations | 12 | 2.28 (1.11-2.71) |
| Septic shock | Infections and infestations | 35 | 2.23 (1.61-2.57) |
| Hepatocellular injury | Hepatobiliary disorders | 16 | 2.2 (1.22-2.62) |
| Neurotoxicity | Nervous system disorders | 15 | 2.19 (1.18-2.62) |
| Aspartate aminotransferase increased | Investigations | 33 | 2.18 (1.55-2.54) |
| Alanine aminotransferase increased | Investigations | 39 | 2.14 (1.56-2.48) |
| Deafness | Ear and labyrinth disorders | 21 | 2.12 (1.3-2.53) |
| Disease progression | General disorders and administration site conditions | 87 | 2.07 (1.7-2.32) |
| Pancytopenia | Blood and lymphatic system disorders | 38 | 2.06 (1.48-2.41) |
| Oral candidiasis | Infections and infestations | 9 | 2.05 (0.74-2.57) |
| Metastases to central nervous system | Neoplasms benign, malignant and unspecified (incl cysts and polyps) | 9 | 1.98 (0.69-2.52) |
| Neuropathy peripheral | Nervous system disorders | 71 | 1.95 (1.56-2.24) |
| Mouth ulceration | Gastrointestinal disorders | 14 | 1.95 (0.95-2.44) |
| Hypokalaemia | Metabolism and nutrition disorders | 30 | 1.94 (1.29-2.33) |
| Pleural effusion | Respiratory, thoracic and mediastinal disorders | 40 | 1.92 (1.37-2.27) |
| Cardiovascular disorder | Cardiac disorders | 10 | 1.92 (0.72-2.46) |
| Thrombocytopenia | Blood and lymphatic system disorders | 68 | 1.83 (1.43-2.13) |
| Pyrexia | General disorders and administration site conditions | 217 | 1.83 (1.61-2.01) |
| Full blood count decreased | Investigations | 15 | 1.8 (0.87-2.32) |
| Hyponatraemia | Metabolism and nutrition disorders | 33 | 1.74 (1.15-2.14) |
| Asthenia | General disorders and administration site conditions | 222 | 1.7 (1.49-1.88) |
| Vomiting | Gastrointestinal disorders | 251 | 1.62 (1.43-1.79) |
| Hepatic enzyme increased | Investigations | 34 | 1.56 (0.99-1.97) |
| Anaemia | Blood and lymphatic system disorders | 98 | 1.54 (1.22-1.8) |
