# Supplementary material for: Epirubicin and gait apraxia: a real-world data analysis of the FDA Adverse Event Reporting System database
Source: Front Pharmacol. 2023 Sep 14;14:1249845. doi: 10.3389/fphar.2023.1249845 (PMC10536159; doi:10.3389/fphar.2023.1249845)
Supplement: Supplementary file 6 [file Table6.docx]

**Supplementary Table S6: Other drugs that may cause gait apraxia**

| Pharmaceutical ingredient | Case number |
| --- | --- |
| Epirubicin | 3 |
| Gabapentin | 3 |
| Imatinib | 2 |
| Brexpiprazole | 1 |
| Cyclophosphamide | 1 |
| Insulin lispro | 1 |
| Interferon beta-1a | 1 |
| Memantine hydrochloride | 1 |
| Pimavanserin tartrate | 1 |
| Ranibizumab | 1 |
| Regorafenib | 1 |
| Rivastigmine | 1 |
| Sodium oxybate | 1 |
| Tacrolimus | 1 |
| Trastuzumab | 1 |
| Valproate sodium | 1 |
| Carbidopa\levodopa | 1 |
